# Acknowledgment to Reviewers of *Materials* in 2021

**DOI:** 10.3390/ma15031131

**Published:** 2022-02-01

**Authors:** Materials Editorial Office

**Affiliations:** MDPI AG, St. Alban-Anlage 66, 4052 Basel, Switzerland

Rigorous peer-reviews are the basis of high-quality academic publishing. Thanks to the great efforts of our reviewers, *Materials* was able to maintain its standards for the high quality of its published papers. Thanks to the contribution of our reviewers, in 2021, the median time to first decision was 15 days and the median time to publication was 38 days. The editors would like to extend their gratitude and recognition to the following reviewers for their precious time and dedication, regardless of whether the papers they reviewed were finally published:
Aamir, MuhammadLi, Tomi T.Abad, BegoñaLi, WeiAbali, Bilen EmekLi, WeixinAbashev, George G.Li, XinweiAbate, AndreaLi, ZhongAbbas, NaseemLian, Ian Y.Z.Abbas, QamarLiang, DaweiAbbasali, Taghavi GhalesariLiang, Goh GuoAbbass, WaseemLiang, JunAbbassi, FethiLiang, RuijingAbbaszadeh, ShivaLiang, ShuaiAbd El-Mageed, TaiaLiang, Shun-XingAbdallah, ImanLiang, WeixiongAbdelgader, HakimLiao, Chao-YaugAbdelhafeez, Ali M.Liao, Chien-SenAbdelhamid, Hani NasserLiao, JiaxuanAbdelkader, HamdyLiao, Jiunn-DerAbdelmohsen, Usama RamadanLiao, MeiyongAbdel-Mohti, AhmedLiauzun, CedricAbd-Elrahim, Ahmed GalalLiaw, Peter K.Abdelrahman, MeramLibante, VirginieAbdo, PeterLiber-Kneć, AnetaAbdulgader, MohamedLichý, PetrAbduljabbar, TariqLicini, CaterinaAbdullah, DaliaLigabue-Braun, RodrigoAbdullah, Johari YapLignola, Gian PieroAbdullah, Mohd Mustafa Al BakriLiguori, FrancescoAbdullah, Nur AzamLikodimos, VlassiosAbdullah, Oday IbraheemLillepärg, JelenaAbdulwahid, RebarLim, DanielAbduo, JaafarLim, Ho-KyungAbdur, RahimLim, Jae HyukAbed, FaridLim, JeeyoungAbedin, FarhanaLIM, Jun LongAbedini, ArminLim, SeokbinAbedini-Nassab, RoozbehLim, Siew YeeAbellan-Nebot, Jose VicenteLima, Maria J.Abid, MushriqLima, RuiAbidi, NoureddineLima, SofiaAbiev, RufatLimiti, ErnestoAbojassim, AliLimongi, TaniaAbondio, PaoloLin, Chang-ShengAboraia, Abdelaziz M.Lin, Che-YuAbotaleb, IbrahimLin, Chia-HerAbou Elmaaty, Tarek M.Lin, Chia-MinAbouelmagd, Elbaz I.Lin, Chien-hongAbouelregal, Ahmed E.Lin, Chung-KweiAbouhussien, AhmedLin, Chun-HoAbououalid, HichamLin, Chun-PinAbramczyk, JacekLin, Chun-RongAbramenko, NataliaLin, Gong-RuAbramov, Pavel A.Lin, GuanAbramovich, HaimLin, HonghongÁbrányi-Balogh, PéterLin, Jhe-YuAbrashov, AlekseyLin, JiaAbrosimova, GalinaLin, Kae-LongAbu Sohel, KaziLin, Meng-FangAbubakr, Neamat HassanLin, Muh-ShiAbu-Dief, Ahmed M.Lin, PengAbundo, RobertoLin, ShaoyangAburaia, MohamedLin, Tz-FengAbu-Reziq, RaedLin, Wei-ChunAbu-Zeid, NasserLin, YangAccardo, GraziaLindberg, GabriellaAcedos, Miguel G.Lindner, ThomasAcevedo, RogelioLindström, TomAcharya, SwagataLinkevičius, EdgarasAchchaq, FouziaLio, Giuseppe EmanueleAchelle, SylvainLionetti, VincenzoAchilias, Dimitris S.Lionetto, FrancescaAcierno, DomenicoLiparoti, SaraAciu, ClaudiuLipatov, AlexeyAckermann, MichalLipinski, DariuszAdachi, ShinichiroLipiński, TomaszAdak, DibyenduLipovský, PavolAdamczyk, GretaLipsanen, HarriAdamek, GrzegorzLipski, AdamAdamiec, JanuszLiptáková, TatianaAdamu, MusaLira, MadalenaAdawy, AlaaLisa, GabrielaAddiego, FredericLitina, ChrysoulaAdeagbo, Waheed A.Litowczenko, JagodaAdeel, ShahidLiu, BiluAdeel, UmerLiu, Chain T.Adekunle, Timothy O.Liu, ChaoAdemović, NaidaLiu, ChaoxingAdera, SolomonLiu, FupinAdesina, Akeem Y.Liu, HanqiAdhikari, RajdeepLiu, HaojieAdil, MohdLiu, HeliAdilson De Castro, JoséLiu, JunyiAdlhart, ChristianLiu, KaimiaoAdmal, NikhilLiu, MinliangAdriana, SacconeLiu, QianhuiAfdzaluddin, AtiqahLiu, QuanAfify, AhmedLiu, ShichaoAfify, Ahmed SabryLiu, Shou-HengAfiqah Mohd Radzuan, NabilahLiu, TianAfsari, MortezaLizarazo-Marriaga, JuanAftab, AdnanLlanos, IñigoAgarwal, PraveenLlorens, José ManuelAgarwala, ShwetaLo Conte, AntoniettaAgathe, RobissonLo Giudice, RobertoAgbabiaka, OkikiolaLobiuc, AndreiAgbaje, OluwatoosinLocarno, Silvia AliceAgeev, EduardLocci, Antonio MarioAggas, JohnLofaj, FrantišekAghakhani, AmirrezaLofrano, EgidioAgnieszka, MiskiewiczLoghman, AbbasAgnoli, StefanoLoginova, IrinaAgossa, KevimyLogozzo, SilviaAgostiano, AngelaLohmus, RynnoAgrela, FranciscoLoja, Maria Amélia RamosAguado, RobertoLojen, GorazdAguiar, AndréLoka, ChadrasekharAguila, BrianaLokteva, Ekaterina S.Aguilar Romero, FernandoLokuge, WeenaAguilar, MariaLomakin, IvanAguilar-Meléndez, ArmandoLoncaric, IvorAgureev, LeonidLongo, AngelaAgustin, DominiqueLonkwic, PawełAhlfeld, TilmanLoontjens, Ton J. A.Ahmad, AfaqLopes, Adelino V.Ahmad, AkilLopes, Elsa BAhmad, JamlehLopes, NunoAhmad, TaufiqLopes, Sergio M.R.Ahmadi, NimaLópez Garcia, José ManuelAhmadkhaniha, DonyaLopez Gayarre, FernandoAhmed, AsifLopez Quintas, IgnacioAhmed, EssamLopez, Gabriel AlejandroAhmed, FaizLópez, JulioAhmed, Hawreen HasanLópez-Carreño, Rubén-DanielAhmed, MizanLopez-Cuesta, José-MarieAhmed, Mohamed Mohamed ZakyLópez-Ferreño, IñakiAhmed, Mohammad BoshirLopez-Garzon, RafaelAhmed, TousifLopez-Linares, FranciscoAhn, Chang WonLopez-Maldonado, Eduardo A.Ahn, SeokHwanLopot, FrantišekAhn, Yeong HwanLops, DiegoAhola, AnttiLorenz, MichaelAhsan Saeed, MuhammadLorenzo, JuliaAhsani, VahidLorenzo, MiguelAhuir-Torres, Juan IgnacioLorusso, FeliceAhumada, GuillermoLorusso, MassimoAiello, FedericaLosada-Perez, PatriciaAiroudj, AissamLosanno, DanieleAissa, BrahimLöser, WolfgangAǐzikovich, Sergey M.Losio, SimonaAjejas, FernandoLota, KatarzynaAjmal, Hafiz Muhammad SalmanLott, DieterAkay, GalipLoukopoulos, EdwardAkazawa, MasamichiLounsbury, NicoleAkbari, AliLoutas, Theodoros H.Akhlaghi, OmidLouzguine, Dmitri V.Akhmadullina, Nailya S.Lowndes, RobertAkil, SuzannaLozano, AdriánAkilbekova, DanaLozano-Lunar, AngélicaAkimov, Sergey A.Lu, HaoAkinwamide, Olukayode SamuelLu, HuiAkinyemi, BanjoLu, QingAkkapol, Suea-NgamLu, WenchaoAkkouch, AdilLu, YubingAkram, NadiaLü, YupengAkram, ShakeelLubatsch, AndreasAksakal, ResatŁubiński, JacekAksenov, SergeyLuca, DorinAkyurtlu, AlkimLucaciu, Patricia OndineAl Aukidy, MustafaLucchetta, GiovanniAl Bakri Abdullah, Mohd MustafaLucenti, ElenaAl Zoubi, WailLuchian, IonutAlaboodi, Abdulaziz S.Luciano, GiorgioAl-Ahmad, AliLúcio, MarleneAlam, Arif UlLucyshyn, ThomasAlam, M. ShafiulŁuczak, JustynaAlam, Md NajibLugo, Jesus EduardoAlam, MehebubLuhar, SalmabanuAlam, MohammadLui, EricAlamatian, JavadLukac, PavelAlamri, SabriLukacs, JanosAlapati, Satish B.Lukács, JánosAlar, VesnaŁukowski, PawełAl-Asbahi, BandarLuna, DiegoAl-Attar, NebrasLund, HenrikAlava, MikkoLunt, AlexanderAlay-E-Abbas, Syed MuhammadLunt, Alexander J.G.Alazzam, AnasLuo, HongxiAlbano, GianluigiLuo, KunAlbero, JosepLuo, QuanshunAlberto Della Rovere, CarlosLuo, TianyiAlberto Gallardo-Vega, CarlosLuo, WenAlbizuri, JosebaLupi, Saturnino MarcoAlbonetti, CristianoLupo, DonaldAlborés, SilvanaLuque-Agudo, VerónicaAlbrecht, RobertLuso, EduardaAlbrektsson, TomasLutter, Jacob C.Albu, MihaelaLuty-Błocho, MagdalenaAlcaraz, LorenaLuzi, FrancescaAlcock, BenLyanguzova, IrinaAldazabal, JavierLyer, StefanAldewachi, HasanLynch, WillAlducin-Ochoa, Juan ManuelŁyszczek, RenataAlegre, CinthiaŁyszkowski, RadosławR.Alegret, NuriaLyubenova, Teodora StoyanovaAlejandra, Mazo M.Ma, BinhuiAlejandro-Martín, SergueiMa, ChiAleksi, IvanMa, GuangyiAl-Enizi, Abdullah M.Ma, JunAleshin, VladislavMa, RanAlexa, ErsiliaMa, ShuhuaAlexander Yurievich, IvannikovMaalouf, ChadiAlexandre, JonasMaas, JürgenAlexandropoulos, DimitrisMaccagno, MassimoAlexandrovna, Skotnikova MargaritaMaccato, ChiaraAl-Fakih, AminMacdougall, ColinAlfè, MichelaMacek, WojciechAlfei, SilvanaMaciejewska, MałgorzataAlgadi, HassanMacKenzie, Kenneth J.D.AlHumaid, JehanMackie, AllisonAli, BabarMackiewicz, PiotrAli, BaghMacutkevič, JanAli, Basant A.Madadi, AmirhosseinAli, GomaaMadbouly, SamyAli, HaiderMadej, DominikaAli, KhatibiMadej, MarcinAli, Md. HazratMadheswaran, ThiagarajanAli, SamimMadi, MarwaAliasghari, SepidehMadruga Saavedra, Francisco JavierAlikin, DenisMadruga, SantiagoAlim, Mohammad AbdulMaeda, HatsuhikoAlimirzaloo, ValiMaestá Bezerra, FabricioAlix, SébastienMaestre Pérez, Salvador E.Alizadeh, AliakbarMaffei, NicolaAlkahtani, MasferMagagnosc, Daniel J.Alkhatib, IsmailMagalhães, Fernão D.Al-Kheetan, MazenMagalhães, JoanaAllahverdiyeva, YagutMagdalena, Musuc AdinaAllahyari, HamedMagkoev, Tamerlan T.Allam, TarekMagnaudeix, AmandineAllen, Norman S.Magrinho, João P.Allen, TanuMahankali, KiranAlmaev, Aleksei V.Mahapatra, ChinmayaAlMangour, BandarMahapatra, Manoj K.Al-Mansoori, TariqMahata, ChandreswarAlmaral-Sanchez, Jorge L.Mahdavi, MaziarAl-Marzouqi, Ali H.Mahmood, HaroonAlmas, KhalidMahmoud, Alaa El DinAlmásy, LászlóMahmoud, KaremAl-Mdallal, Qasem M.Mahovic Poljaček, SanjaAlmeida, ArmindaMahy, Julien G.Almeida, Bernardo GonçalvesMaiborodin, IgorAlmeida, HumbertoMaidaniuc, AndreeaAlmeida, JoanaMaier, CatalinaAlmendro-Candel, Maria BelénMaimistov, Andrei I.Almeraya-Calderón, FacundoMaior, IoanaAlmohammed, SawsanMaisch, TimAlmonti, DanieleMaitarad, PhornphimonAlmpanis, E.Maiti, DebtanuAlnedawi, AliMaity, ParthaAloisio, AngeloMaj, MichałAlosmanov, RasimMajd, Alireza EslamiAlpatov, VadimMajerič, PeterAl-Qarni, Faisal D.Majerik, JozefAlrwashdeh, SaadMajerski, KrzysztofAl-Saadi, SaadMajewski, TomaszAlshahrani, Hassan A.Majhi, Rajib KumarAlshoaibi, Abdulnaser M.Majidi, RoyaAltaf, KhurramMajkowska-Marzec, BeataAltamash, TausifMajor, IzabelaAltan, M. CengizMajor, MaciejAltarawneh, Mohammednoor KhalilMajtan, JurajAltman, IgorMajumder, PoulamiÁlvarez, AnaMajumder, SutriptoAlvarez, GonzaloMakarian, KamranAlvarez, JoséMakarov, DmitriyÁlvarez, José AlbertoMakarova, Anna S.Álvarez, María S.Makaryan, TaronAlvarez, PedroMakhmudov, Khairullo FaizullaevichÁlvarez-Alonso, PabloMakomaski, GrzegorzÁlvarez-Antolín, Jose FlorentinoMakoś, PatrycjaÁlvarez-Ayuso, EstherMaksimović, AleksandarAlves De Sousa, Ricardo J.Maksimovic, VesnaAlves Galico, DiogoMakuch, EdytaAlves, FilipeMakul, NattAly, KarimMałachowska, AleksandraAlyousif, Osama M.Malachowski, JerzyAl-Zand, Ahmed W.Malaiškienė, JurgitaAlzangana, Shakhawan Hassan AliMalara, AngelaAmador, UlisesMalatesti, NelaAmaechi, Chiemela VictorMalça, CândidaAmaral, Rui L.Malchik, FyodorAmaro, PedroMalecha, KarolAmbrosi, AlanMalekan, MohammadAmbroziak, AndrzejMaleki, ErfanAmemiya, KentaMalekmohammadi, HamedAmendola, AdaMaleknia, Simin DAmer, MohammedMaletti, SebastianAmiandamhen, StephenMałgorzata, Skucha-NowakAmin, Muhammad NasirMali, GregorAmin, WaseemMalik, SharaliAmine, AzizMalikan, MohammadAmir, AbdiMalinowski, SzymonAmiri, OualiMaliszewski, MaciejAmmar, HanyMaljaee, HamidAmmarullah, Muhammad ImamMalka, DrorAmos, P.G. KubendranMalkin, AlexanderAmza, CatalinMalladi, SriramAn, GyubaekMallick, Md MofasserAn, QiMallis, PanagiotisAn, SangminMałolepsza-Jarmołowska, KatarzynaAn, Sang-MinMalomo, DanieleAn, XuehuiMalvè, MauroAna Maria Cristina, TancuMalvic, TomislovAna, CuestaMałysz-Cymborska, IzabelaAnanth, AntonyMamba, GcinaAnanthakrishnan, Soundaram JeevarathinamManaenkov, OlegAnastasiadis, AnthimosManara, DarioAnastasiou, EleftheriosMancini, SimoneAnatskaya, Olga V.Mandal, ParanjayeeAnawati, AnawatiMandal, SoumenAnaya, Nelson M.Mandelli, DavideAnbalagan, Aswin KumarMandić, VilkoAnbazhagan, SathiyaseelanMandrino, DjordjeAnca, FilimonManecki, MaciejAnceschi, AnastasiaManfredi, GiovanniAnders, DenisMangiafico, SalvatoreAndersen, Sigmund JarleMani, GovindasamyAndrade, JairoMani, RajaboopathiAndrade, JorgeManian, Avinash P.Andreev, Vladimir V.Manickam, RamachandranAndreev-Andrievsky, AlexanderMánik, TomášAndrei, GabrielManika, GeorgiaAndrei, Radu DorinManini, PaolaAndreiadis, EugenManjunatha, Krishna NamaAndreoni, AlessioMankovits, TamásAndrew, JeffersonManlapig, EmmanuelAndriolo, Jessica M.Mannari, VijayAndriotis, Eleftherios G.Manoach, EmilAndronic, LuminitaManolea, HoriaAndrukhov, OlehManos, George C.Andryushin, Konstantin P.Mansi, AntonellaAndrzejewska, AngelaManso, Maria ConceiçãoAnes, VitorMantell, CasimiroAnesi, AlexandreManthos, EvangelosAnfinogentov, VladimirMantyjarvi, KariAngeletti, MauroManuel, Pascual GuillamónAngelopoulou, AthinaManzano-Moreno, Francisco JavierAngelov, BorislavManzhos, SergeiAngiolilli, MicheleMao, AngeloAngizi, ShayanMao, ChuanbinAnglani, GiovanniMao, GaojunAngurel, Luis A.Mao, JiajiaAnikeev, Sergey G.Mapelli, CarloAnil, SukumaranMarani, AfshinAnita Ioana, VisanMarascu, ValentinaAnitas, Eugen MirceaMaraveas, ChrysanthosAnjani, Qonita KurniaMarceau, SandrineAnn, Ki YongMarcellini, MorenoAnnalisa, Natali MurriMarchelli, FilippoAnraku, MakotoMarchenko, EkaterinaAnsari, Mohammad JavedMarchenkov, ArtemAnsell, Troy Y.Marchese, GiulioAnsón Casaos, AlejandroMarciniec, BogdanAntico, FedericoMarcinkowska, AgnieszkaAnton, DavydokMarcisz, JaroslawAntón, María NatividadMarconcini, PaoloAntonatos, NikolasMarconi, Guya DilettaAntonelli, AlessandroMarcos, CeliaAntoni, AntoniMarcu, AurelianAntoniac, IulianMardare, Andrei IonutAntoniak-Jurak, KatarzynaMare, AncaAntonino, FamulariMarega, CarlaAntonoglou, Georgios N.Marenzi, GaetanoAntonyshyn, IrynaMargaritis, AlexandrosAntosik, AdrianMargaritis, NikolaosAntošová, NaďaMaria Del Pilar, Villar CastroAntosz, KatarzynaMaria, BidikoudiAntoszewski, BogdanMariazzi, SebastianoAntretter, ThomasMarichev, KostiantynAntunes, JoanaMariconda, AnnaluisaAntunes, VanessaMariello, MassimoAntunes, VítorMarimuthu, UthayakumarAnvari, BahmanMarin, MarianaAnyszka, RafałMarinova, IlianaAnzueto-Sánchez, GilbertoMarinova, VeraAoh, Jong-NingMariotti, DavideAoyagi, KentaMarkiewicz, AnnaAoyagi, ShinobuMarkiewicz, RoksanaAparicio, Francisco J.Markin, AndreyApel, DanielMarkou, AnastasiosAperador, WilliamMarkovsky, PavloApetrei, ConstantinMarkowski, Piotr MarekApicella, AntonioMarmiroli, BenedettaApollo, NicholasMarocco, AntonelloApostol, MarianMarônek, MilanApostolopoulos, CharisMarousek, JosefAppino, CarloMarquardt, AxelAprile, AlessandraMarques De Oliveira Ferraz, Eduardo JorgeAprilia, ApriliaMarques Dos Santos, João MiguelAquilano, DinoMarques, Ana ClaraAquino, Rita PatriziaMarques, DuarteAraci, SerkanMarques, RosaArafa, AhmedMarrocchino, ElenaAragay, GemmaMarrocco, ValeriaArahira, TakaakiMarshal, AmalrajArain, SalmanMarsili, EnricoArakawa, YukiMartău, Adrian GheorgheAranda-Ruiz, JosueMartín Martínez, José MiguelAraujo, JeffersonMartín, Antonio J.Araujo, PatriciaMartín, CristinaAraujo-Moreira, Fernando M.Martín, DomingoAraya, GerardoMartín, Domingo AlfonsoAraya-Hermosilla, Esteban AlejandroMartin, NicolasArchibong, Archibong EsoMartin, PascalArchMiller, Althea A.Martín-de León, JudithArdelean, Ioan I.Martin-Del-Rio, Juan JesusArea, María CristinaMartinelli, DanieleArede, AntonioMartinelli, EnzoArefi, MohammadMartínez Barrera, GonzaloArena, MaurizioMartinez, AlfonsoArgiz, CristinaMartínez, Félix QuinteroArias-Pardilla, JoaquínMartínez-Camarena, ÁlvaroAriga, KatsuhikoMartínez-García, RebecaArima, TakahisaMartínez-González, Jose AArith, FaizMartínez-Lillo, JoséArjmandi, RezaMartinez-Marquez, DanielArjunan, ArunMartínez-Martínez, DiegoArmaghani, Danial JahedMartínez-Martínez, VirginiaArnaud, RecoquillayMartinez-Pañeda, EmilioArnold, Donna C.Martínez-Periñán, EmilianoArnusch, ChristopherMartínez-Tong, Daniel E.Aromaa, JariMartín-García, Juan ManuelArora, HimanshuMartinho, Fernando C. G.Arrabito, Giuseppe DomenicoMartino, Nicola AntonioArrigo, RossellaMartins Amaro, Ana Paula BetencourtArroyo Martínez, BorjaMartins Morgado, Teresa LeonorArruda, Gilberto JMartins, FranciscoArruebo, ManuelMartins, Jorge N.R.Arshad, MuhammadMartins, MarcosArshad, Muhammad AzeemMartins, Marcos SilvaArsiccio, AndreaMartins, Ramiro J.E.Arteagoitia, IciarMartín-Sánchez, JavierArtemev, AndreiMarto, Carlos MiguelArtola, GarikoitzMartoïa, FlorianArul, Narayanasamy SabariMárton, KivovicsArumugam, VeerakumarMartucciello, NadiaArunachalam, ShivaramMartynenko, Natalia S.Arya, SandeepMaruda, Radosław W.Asa′ad, FarahMaruthamuthu, MuraliAsadi, MojtabaMaruyama, IppeiAsadpour, SaeidMarvi-Mashhadi, MohammadAsaji, TetsuoMarzec, IreneuszAsakura, TakumiMarzouk, OsamaAsala, GbengaMasai, HirokazuAsano, AtsushiMasato, DavideAsaro, FiorettaMashaan, NuhaAscensão, GuilhermeMashentseva, AnastassiaAschilean, IoanMasi, GiuliaAscione, FrancescoMasi, SofiaAseev, Vladimir AnatolevichMaslak, MariuszAsenath-Smith, EmilyMašláni, AlanAshida, FumihiroMasłoń, AdamAshkenazi, DanaMaslov, MikhailAshraf, WardaMasood Chaudry, UmerAshrafi, NarimanMaspero, CinziaAshuri, MaziarMasruchin, NanangAsimakopoulos, Ioannis A.Massa, AntonioAslfattahi, NavidMassad-Ivanir, NaamaAšmontas, SteponasMassányi, PeterAssaad, JosephMassella, DanieleAssadi, AymenMassenio, Paolo R.Assaf, BadihMastalska-Popławska, JoannaAssender, HazelMastino, Costantino CarloAstakhov, Viktor P.Mastrotto, FrancescaAsubar, Joel T.Masud, JahangirAsuri, PrashanthMasuda, KenichiAta, SeisukeMasui, ToshiyukiAtabaev, Timur Sh.Masuno, AtsunobuAtaei, AbdolrezaMatadi Boumbimba, RodrigueAtamas, NataliaMateescu, Andreea LorenaAtanasov, VasilMatei, AndreeaAtanasov, VladimirMatejíček, JiříAtaollahi, NargesMatějka, VlastimilAtaya, SabbahMateo, AntonioAtchudan, RajiMateo, DiegoAthanasiou, Christos-EdwardMatešan, DomagojAtilgan, AhmetMathieu, MarchivieAtkinson, IrinaMatić, MirelaAtlaskin, Artem A.Matko, VojkoAtrens, AndrejMatos, Juan-CarlosAttanasio, CarmineMatras, AndrzejAtta-Obeng, EmmanuelMatsidik, RukiyaAttari, FarnooshMatsubara, HirokiAttoui, MichelMatsubara, KoukiAtuchin, VictorMatsugaki, AiraAtzori, MatteoMatsumoto, ShojiAubert, EmmanuelMatsuno, TomonoriAuf Der Maur, MatthiasMatsuo, HirokiAuffermann, GudrunMatta, RagaiAuger, ThierryMattsson, CeciliaAurelio, BifulcoMatula, GrzegorzAutelitano, FedericoMatuła, IzabelaÁvalos-Ovando, OscarMaturavongsadit, PanitaAvarvari, NarcisMatúš, MilošAvdonin, Pavel V.Matusiak, JakubAverbukh, MosheMatuszewska, AgnieszkaAverina, Elena B.Matuszewska, DominikaAvetissov, IgorMatykiewicz, DanutaAvgouropoulos, GeorgeMatys, SabineAvilés, María-DoloresMauceri, RodolfoAviziotis, Ioannis G.Mauder, TomášAvolio, RobertoMauriz, ElbaAvramescu, Sorin MariusMaurizio, SabbatiniAvramopoulos, AggelosMauro, MatteoAwad, AtheerMaurotti, SamanthaAwd, MustafaMaury-Ramírez, AníbalAwoyera, Paul OluwaseunMautner, AndreasAxelevitch, AlexanderMavila, SudheendranAyadi, AbderrahmaneMaximenko, AndreyAyankojo, Akinrinade GeorgeMaximov, JordanAyar, PooyanMaxineasa, Sebastian GeorgeAyaso, Francisco-JavierMay, MichaelAydin, GokhanMayeli, PeymanAydinyan, SofiyaMayer, ChristianAydoğdu, AyçaMayer, HerwigAygörmez, YurdakulMayer-Gall, ThomasAyuso Sebastian, Miguel AythamiMayordomo, RaquelAyyoob, MuhammadMayuet, Pedro F.Azam, MohammadMayumi, KoichiAzarsa, PejmanMayyahi, Ahmed AlAzimi, BaharehMaza, DiegoAziz, FaissalMazalova, Victoria L.Aziz, TariqMazare, AncaBaba, KoumeiMaździarz, MarcinBabaei, HashemMazeika, KestutisBabafemi, Adewumi JohnMazer, WellingtonBabatunde Alayande, AbayomiMazumde, Mohammad Abu JafarBabu, DineshMazurek, GrzegorzBacalum, MihaelaMazurek, KrzysztofBacci, ChristianMazzanti, PaolaBachagha, TarekMazzaracchio, VincenzoBack, Jong-HoMazzi, AlbertoBadanoiu, Alina IoanaMazzone, GiuseppeBadilescu, SimonaMazzone, GloriaBadogiannis, EfstratiosMazzucco, GianlucaBadshah, Mohsin AliMbey, Jean AimeBae, DongsuMbuya, Thomas O.Bae, Hyung D.McCann, FinianBae, Jae WungMcCann, RonánBae, Jae YoungMcCoy, Thomas M.Baek, Jong-SuepMcDevitt, Sylvia FrankeBaek, UnbongMcIntyre, GarryBaena, Francisco ManuelMckay, TinaBagdziunas, GintautasMcLellan, IainBaghdadi, Amir HosseinMcManamon, PaulBagheri, AliMd Nor, NoorsuhadaBagheri, RezaMebarek Oudina, FatehBagryanskaya, ElenaMech, RafałBah, MickaMechrez, GuyBahador, AbdollahMedarevic, DjordjeBahat-Treidel, EldadMedina, AriostoBahiuddin, IrfanMedina, CésarBahr, DavidMedina, DavidBahuleyan, Bijal K.Medina-Cruz, DavidBai, ChengyingMedunić, GordanaBai, HongweiMedvedev, AndreyBai, MingwenMedyński, DanielBai, RuixiangMeesorn, WorarinBai, YuchaoMeghil, MohamedBaig, Khurram ShahzadMegna, BartolomeoBaig, UmairMehboob, HassanBailkov, Daniel AMehdi, EbrahimiBaitimerov, RustamMehdizade, MohammadBajnóczi, BernadettMehmeti, AndiBajor, TeresaMehrizi, Abbasali AboueiBajorek, AnnaMehta, Yusuf A.Bajpai, AnkurMei, BastianBajwa, DilpreetMei, DiBakarčić, DankoMei, HanfeiBakator, MihaljMei, LeiBaker, IanMeisenheimer, PeterBaker, PeterMeister, JörgBakonyi, ImreMejía-Ruíz, Claudio HumbertoBakr, MahmoudMelcer, JozefBaktheer, AbedulgaderMelikhov, Vladimir I.Balabanov, VladimirMelin, FredericBalachandran, VinithaMelin, TomasBalamurugan, RathinamMelinte, VioletaBalan, AdrianaMelnikov, Alexander S.Balar, NrupMelnikov, StanislavBalashova, Elena V.Melo, MirianBalasoiu, MariaMelro, António RuiBalasubramani, NagasivamuniMemisoglu, GorkemBalcar, HynekMemmolo, VittorioBalcoş, CarinaMena-Morcillo, EmmanuelBaldassari, SaraMenazea, AbdelrhmanBaldelli, AlbertoMencik, Jean-MathieuBaldo, NicolaMendes, AnibalBaldovino, JairMendes, Joana CatarinaBalduzzi, GiuseppeMéndez, José AlbertoBaliello, AndreaMendoza Reales, Oscar AurelioBálint, ErikaMendoza, AbrahamBaliti, JamalMendoza, DanielBallantyne, AndrewMendoza, Rose Marie O.Ballesteros-Garrido, RafaelMenezes, PradeepBalliana, EleonoraMeng, DongBalmori Roiz, Jose AntonioMeng, FanqiangBalobanov, ViacheslavMeng, SiqiBalogh-Weiser, DianaMenga, NicolaBalos, SebastianMenni, YounesBaltakys, KęstutisMensah, Enoch A.Baltatu, Madalina SimonaMensik, MiroslavBaltazar-Zamora, Miguel AngelMentrasti, LandoBalusu, RaoMenzler, NorbertBalzer, FrankMeocci, MonicaBambach, MikeMerazzo, KarlaBanciu, Cristina AntonelaMercader-Moyano, PilarBanciu, Marian GabrielMercado-Colmenero, Jorge ManuelBandelt, Matthew J.Merchán, María DoloresBandyopadhyay, SupriyoMercorelli, PaoloBanea, Mariana DoinaMere, ArvoBanerjee, AnilMerhar, MiranBanerjee, DisharyMerie, Violeta ValentinaBanerji, SourangsuMerkulov, Daniela ŠojićBani, DanieleMerrett, Craig G.Baniotopoulos, CharalamposMerrin, JackBannov, AlexanderMerson, EvgeniyBano, GregorMertens, AnneBañón, FermínMertens, ChristianBansal, KuldeepMertens, StijnBao, WeizhaiMertzimekis, TheoBao, WenzhongMesa, JaimeBao, YongmingMesguich, DavidBao, YuhaiMessias, AnaBao, ZhenghongMeto, AidaBar, MahadevMetz, RenaudBar, SukantaMetzker, GustavoBaraba, AnjaMewes, JanBarabanova, Anna I.Meyer, FlorentBarabás, RékaMezhuev, Ya. O.Barabas, SorinMežibrický, RolandBarabash, RozaliyaMezzetta, AndreaBarabaszová, Karla ČechMezzi, AlessioBarai, Hasi RaniMhadhbi, MohsenBaraldi, DanieleMi Lee, BoBaranek, PhilippeMi, Fwu-LongBaranović, GoranMiądlicki, KarolBarbalexis, PanagiotisMiah, Md JihadBarbaro, KatiaMiao, BingrongBarberis, AntonioMiao, GuoxingBarberis, FabrizioMiao, YinghaoBarbi, SilviaMiao, YuBarbieri, GiuseppeMiarka, PetrBarbinta-Patrascu, Marcela ElisabetaMicaelo, RuiBarbon, SilviaMican, SeverBarbosa De Lima, Antonio GilsonMicciulla, FedericoBarbosa, JoaquimMiccoli, StefanoBarbosa, Jorge Luís VictóriaMicek-Ilnicka, AnnaBarbot, Jean FrancoisMichał, BartmańskiBarboutis, Ioannis A.Michal, MalčekBarbudo, AuxiMichał, PreisnerBarcellos, DiegoMichalcová, AlenaBarcikowski, MichałMichalczewski, RemigiuszBarea, RafaelMichalczyk, JacekBarek, JiriMichalczyk, RafałBarettin, DanieleMichałek, JarosławBargan, AlexandraMichalek, KarelBari, EliaMichalik, JanBarik, SabyasachiMichalowska, JoannaBarisic, IvanaMichalski, JerzyBarisic, KarmelaMichaud, Jean-FrancoisBarkauskas, JurgisMichaux, SimonBarkov, RuslanMichele, BacciocchiBarmpatza, Alexandra C.Micheletto, GiancarloBarnes, EftihiaMichna, AnetaBarona Dorado, CristinaMicnas, RomanBarootchi, ShayanMicutz, MarinBarouni, AntigoniMidya, RivuBarragán, V. MaríaMieczkowski, GrzegorzBarres, ClaireMiedzińska, DanutaBarrino, FedericoMielewczyk-Gryn, AleksandraBarriobero-Vila, PereMiernik, KrzysztofBarron, Ana Laura LunaMierzejewska, Żaneta AnnaBarroso, AlbertoMigita, SatoshiBarry, CarolMihai, CruceruBarshtein, GregoryMihai, PetruBarski, MarekMihali, CristinaBarszczewska-Rybarek, IzabelaMihály, JudithBartczak, PrzemyslawMija, AliceBartha, CristinaMijo, NikolicBartha, KristínaMijovic, BudimirBartkowiak, GrażynaMikhailov, EvgenyBartkowiak, TomaszMikhailov, Gennadii GeorgievichBartkowska, AnetaMikhaylov, AlexeyBartkowski, DariuszMiki, HajimeBartmański, MichałMikkelsen, OveBartnicki, JarosławMiklaszewski, AndrzejBartocha, DariuszMikolajczyk, TadeuszBartoli, MattiaMikołajczyk, TomaszBartolome, JoseMikolasek, DavidBarton, MichaelMikšová, RomanaBartuli, CeciliaMikucioniene, DaivaBartzas, GeorgiosMikusova, MiroslavaBarylski, AdrianMilad, AbdalrhmanBasak, Animesh KumarMilani, PaoloBasan, RobertMilasius, RimvydasBasarab, MikhailMilčius, DariusBasaran, CemalMilcovich, GesmiBasarić, NikolaMileiko, Sergei T.Baselga Llidó, JuanMiletić, Goran I.Bashir, Muhammad AhsanMiletic, IvanaBasiak, EwelinaMilia, EgleBasile, VitoMilioto, StefanaBasma, HusseinMiljevic, BojanBassetti, MauroMilkovič, OndrejBassini, EmilioMilla, JoseBäßler, RalphMiller, WolframBastola, Anil K.Millo, OdedBastos-Arrieta, JulioMills, Amanda C.Batchelor, PaulMills, KenBatin, GabrielMilovic, LjubicaBatista, André Ulisses DantasMilović, Miloš D.Batista, GrazielaMin Kyoon, ShinBatool, FarnazMin, Jung-hongBator, GrażynaMin, Kyung-SanBatory, DamianMin, RuiBatra, PuneetMinakov, Alexander A.Battiato, AlfioMinakshi, ManickamBattocchio, ChiaraMindaugas, GedvilasBattu, Anil KrishnaMindur, BartoszBauer, A. J.Minea, Alina AdrianaBauer, Jose RobertoMingareev, IlyaBaumann, Jean-SébastienMinho, O.Baumeister, JoachimMinouei, HosseinBaumgarten, MartinMintz, BarrieBaumli, PeterMiraglia, SalvatoreBauser-Heaton, HollyMiranda, Edson Jansen, Jr.Bavaro, TeodoraMiranda, RodolfoBavasso, IreneMiras, Haralampos N.Bayer, IlkerMirela, GalićBaykara, HaciMiri, AminBaykov, SergeyMirković, MiljanaBayón, RocíoMironeasa, SilviaBayraktar, EminMironov, SergeyBazylińska, UrszulaMiruszewski, TadeuszBazzaz, MohammadMirwald, JohannesBeatty, Mark W.Mirza, JuliaBeben, DamianMirzababaie Mostofi, TohidBecerra, JavierMirzadeh, HamedBechelany, MikhaelMirzaei, AliBechir, AnamariaMishigdorzhiyn, UndrakhBeck, Faith R.Mishra, AvanishBedekar, VikramMishra, Geetesh K.Bednarski, HenrykMishra, NigamBednarz, ArkadiuszMishra, RajeshBedo, TiborMishra, VibhorBedolla-Jacuinde, ArnoldoMisiunaite, IevaBeghetto, ValentinaMiskiewicz, MikolajBegić Hadžipašić, AnitaMisra, AmitBegic-Hajdarevic, DerzijaMissell, Frank PatrickBehbahani, Amir HosseinMistewicz, KrystianBehera, KartikMiszczyk, AndrzejBehnamfar, FarhadMitchell, GeoffreyBehnood, AliMitoraj-Królikowska, MarzenaBehrens, Bernd-ArnoMitrev, RosenBehzad Farahani, BehzadMitrica, DumitruBehzadinasab, MasoudMitrofanov, AnatolyBeitz, ToralfMitrokhin, Sergey V.Bejan, DanaMitropoulos, Alexander N.Bejjani, RolandMitsea, AnastasiaBejo, LaszloMittelman, BrigitBejtka, KatarzynaMittova, Irina YaBeke, DavidMitu, BogdanaBeke, DezsoMitukiewicz, GrzegorzBekiari, VlasoulaMityushev, VladimirBel′skaya, LyudmilaMiu, DanaBelan, JurajMiura-Fujiwara, EriBelardi, ValerioMiyazaki, ToshikiBelavič, DarkoMiyazato, ItsukiBelhocine, AliMizera, AlesBelhout, Samir A.Mizerska, UrszulaBell, Nelson S.Mizoshiri, MizueBellantone, VincenzoMizsei, JanosBellini, CostanzoMlonka-Mȩdrala, AgataBellisario, DeniseMlynarczyk, DariuszBellot, Jean-PierreMo, JiliangBellucci, StefanoMo, JinhanBelluzzi, ElisaMoaca, Alina ElenaBelmonte, Miguel Ángel ReyesMobedi, MoghtadaBelo, DulceMobed-Miremadi, MaryamBelogolovskii, MikhailMobili, AlessandraBelousov, Yu. M.Mocanu, StefanBelskaya, Olga B.Mochane, Mokgaotsa JonasBeltrami, RiccardoMochocki, WojciechBeltran, Ana M.Mochugovskiy, AndreyBeltsios, KonstantinosMocioiu, Oana CătălinaBelviso, ClaudiaMoczko, PrzemyslawBelyaev, IvanModigell, MichaelBelyakov, AndreyMoeini, ArashBembenek, MichałMoeini, GazhalBen Ishai, PaulMofidfar, MohammadBenasciutti, DenisMogeritsch, JohannBende, AttilaMoghaddam, Ahmad OstovariBenedek, CsillaMohabeer, ChetnaBenedetti, StefaniaMohaček-Grošev, VlastaBenedic, FabienMohajernia, ShivaBenesperi, IacopoMohamad, DasmawatiBenetatos, PanayotisMohamad, Norzilawati BintiBenić, Ljerka SlokarMohamad-Hussein, AssefBenito, AdriánMohamadian, NimaBenjeddou, OmraneMohamed Kamal Ahmed, AliBenke, MártonMohamed, Hamdy F. M.Bennardo, FrancescoMohammad, AkbarBensalah, NasrMohammad, FaruqBentley, PhillipMohammadi, Mohammad KazemBenyoucef, AbdelghaniMohammed, Ahmed SalihBenzarti, KarimMohammed, Mona S.Bera, SambhunathMohan, MoodBeran, PřemyslMohanavel, VinayagamBerardo, AliceMohapatra, DebanandaBeregoi, MihaelaMohebbi, AbolfazlBerent, KatarzynaMohebbi, Mohammad SadeghBergamo, Edmara T.P.Moheet, Imran AlamBerger, Sandrine BittencourtMohles, VolkerBergfeldt, BrittaMohsen, Marani BarzaniBergqvist, AndersMohyuddin, WahabBergs, ThomasMoiceanu, GeorgianaBeris, AntonyMoiseev, NikitaBerisha, AvniMojaver, KavehBerisha, BekimMojiri, AminBernad, Sandor I.Mokrejš, PavelBernardes, NunoMoldovan, HoraţiuBernardi, SaraMolimard, JérômeBernasconi, RobertoMolina Alcaide, DesireBernasowski, MikołajMolina, Antolino GallegoBernède, Jean ChristianMoliner, CristinaBerretti, EnricoMollenhauer, KonradBert, NikolayMoller, JamesBerta, Gómez-IorMollica, FrancescoBertacco, RiccardoMolnar, OleksandrBertana, ValentinaMolnár, VieroslavBertani, RobertaMolnár, ViktorBerthod, PatriceMombelli, DavideBertocchi, MichaelMomčilović, Milan D.Bertocci, FrancescoMonaco, MichelinaBertoldi, CarloMondal, Amit KumarBertoldo, MonicaMondal, SubhadipBertoncello, PaoloMondal, SudipBertran, Esteve AmatMondeshki, MihailBeskopylny, AlexeyMonetta, TullioBesnard, AurélienMonkova, KatarinaBespalko, Yulia N.Monnier, John R.Bessa, Iuri S.Monobe, HirosatoBessaies-Bey, HelaMonroe, Mary Beth BrowningBeszédes, SándorMonsarrat, PaulBetegón, CovadongaMonserrat, BartomeuBetke, UlfMontalvão, DiogoBetül, ÇalişkanMontanari, MarcoBevitt, JosephMontanari, RobertoBey, AfshanMontanino, AndreaBeyabanaki, ElahehMontans, Francisco JavierBeyabanaki, S. Amir RezaMontaruli, GrazianoBezantakos, SpirosMontasser, Mona A.Bhadra, Biswa NathMonte, María ConcepcionBhalothia, DineshMonteil-Rivera, FannyBhandari, ChurnaMonteiro, Jorge H.Bhandari, ShivaMonteiro, SandraBharath, Kurki NagarajaMontemurro, DomenicoBhargav, AishwaryaMontero, IreneBhatt, PriyankaMontes García, VerónicaBhatti, Muhammad MubashirMontes, J. M.Bhaumik, AsimMontes, RaquelBhosale, Rajesh S.Montes, VicenteBhowmik, PalashMontes-Moran, Miguel A.Bhuckory, ShashiMonticelli, CeciliaBhudolia, Somen K.Montoncello, FedericoBhusal, RamMontuori, RosarioBhuvanesh, SrinivasanMonzó-Cabrera, JuanBi, TiangeMoon, Geon DaeBiagi, MarcoMoon, HokyuBiałobrzeska, BeataMoon, Hong SeokBianchetti, CharlesMoon, JongunBianchi, ArnaudMoon, Joo HyunBianchi, SerenaMoon, Kyoung-SeokBianco, FrancescoMoon, Min SeokBianco, VincenzoMoon, SeonginBianconi, AntonioMoon, Seung KiBica, IoanMoon, SookyoungBickermann, MatthiasMoon, Sung-WooBidarra, Sílvia J.Moraczewski, KrzysztofBidulská, JanaMoradi, Mohammad JavadBidulský, RobertMorais, MauricioBiedunkiewicz, AnnaMorales Conde, María JesúsBieniaś, JaroslawMorales, JuanBiernat, KrzysztofMorales, MiguelBiessikirski, AndrzejMorales, Rodolfo D.Bigerelle, MaxenceMorales-Conde, Maria JesúsBigham, AshkanMorales-Rodríguez, AnaBikbaev, Rashid G.Moramarco, VincenzoBilal, MuhammadMoravčík, RomanBildirir, HakanMoravcikova-Gouvea, LarissaBilgin, AylaMoravec, ZdeněkBilican, DogaMorawiec, MateuszBilisik, KadirMorea, FranciscoBiller, Joshua R.Moreira Jorge, Alberto, Jr.Bilyachenko, Alexey N.Morelli, GiancarloBin Tamrin, Khairul FikriMoreno, DanielBin, YaoMoreno, InésBindra, Jasleen KaurMoreno-Díaz, CristinaBinetruy, ChristopheMorigi, RitaBinh, Nguyen Thi ThanhMorikawa, AtsushiBiris, CristinaMorikawa, SatoruBîrjega, RuxandraMoriyama, YasukoBirke, Kai PeterMorla, ShravanBîrleanu, Corina J.Morlet-Savary, FabriceBirowosuto, Muhammad DanangMorozova, Sofia M.Bischof, SandraMorrell, JeffBishop, Greg W.Morrow, RyanBisio, FrancescoMorse, James R.Bisker, GiliMosa, Ahmed MancyBiswas, ChandanMosaberpanah, Mohammad AliBiswas, Manik ChandraMosavi, AmirBiswas, SantidanMoschopoulou, GeorgiaBiswas, ShantonuMościcki, TomaszBiswas, SoumavaMoscone, DanilaBita, BogdanMoshtaghi, MasoudBitencourt, SandroMoskvichev, EvgenyBittner, FlorianMosleh, AraliyaBituh, TomislavMoslem, Ali IbrahimBizarro Sordo, MonserratMosnackova, KatarinaBizzarri, Bruno MattiaMostafa, AhmadBlacklock, MatthewMostafa, Ayman M.Blais, Jean-FrancoisMostafazadeh, Ali KhosravanipourBlanc, ChristopheMostazo-López, María JoséBlanco, DavidMostoni, SilviaBlanco, IgnazioMotoyanagi, JinBlanco, MatíasMotrescu, IulianaBlandin, RémiMotyka, MaciejBłaszczyński, TomaszMotz, GünterBlatnický, MiroslavMotzki, PaulBlay, VincentMoulick, AmitavaBlazek, JosefMouralova, KaterinaBlázquez, ManuelMourão, Carlos FernandoBleck, WolfgangMourão, PauloBleiner, DavideMousavi Anijdan, HashemBlekhman, DavidMousavi, Seyed SinaBleotu, CoraliaMoussa, SherifBliard, ChristopheMoustafa, EssamBlock, KarinMouzakis, Dionysios E.Blom, JohanMovahedi, NimaBłyskun, PiotrMoya, Antonio A.Błyszko, JarosławMoya, José SerafínBo, ArixinMoysan, JosephBobb, JulianMozaffari, SaeedBober, MariuszMozo Llamazares, Juan DanielBober, PatrycjaMozumder, Mohammad SayemBobrinetskiy, IvanMrňa, LiborBobrowicz, JanMroczyński, RobertBoccardo, GianlucaMróz, KatarzynaBocchetta, PatriziaMroz, RadoslawBocchi, SaraMróz, SebastianBocchieri, SalvatoreMrózek, MariuszBocci, EdoardoMrukiewicz, MateuszBoccia, Antonella CaterinaMrzljak, VedranBocharov, DmitryMuccillo, ReginaldoBochen, JerzyMucha, JacekBochenek, DariuszMück, MichaelBochnia, JerzyMueller, DonaldBochnowski, WojciechMuhaffel, FaizBocian, SzymonMuhammad, RazBociong, KingaMuhammad, RiazBoda, Sunil KumarMukherjee, SantanuBoddupalli, AnuraagMukherjee, ShayantiBoden, StephanMukherjee, SoumyaBodnárová, LenkaMukherjee, SudipBoeffel, ChristineMukhin, NikolayBoffano, MicheleMukhopadhyay, TanmoyBogado, André L.Mukundan, VineethaBogatov, AndreiMulas, GabrieleBogdanov, Alexey M.Müller, AndreiBogdanovich, Valeriy I.Müller, ChristianBogdanovski, DimitriMuller, GerhardBogdanowicz, Krzysztof ArturMüller, KerstinBognár, GabriellaMüller, MiroslavBogojević, NebojšaMun, Sang DonBoguta, PatrycjaMun, Sung CikBohinc, KlemenMuncan, JelenaBöhm, MichałMuneshwar, TriratnaBohm, SivasambuMunir, KhurramBoisse, PhilippeMunir, Muhammad JunaidBoissonnet, GermainMunjiza, AnteBokach, NadezhdaMuñoz, AlfonsoBokan-Bosiljkov, VioletaMuñoz, ÁngelBokias, GeorgiosMuñoz, Daniel PinoBoko, IvicaMuñoz, Jairo AlbertoBolander, JohnMunoz-Pinto, DanyBolbasov, EvgeniyMuñoz-Santiuste, Juan E.Boldin, MaksimMuntean (Koncsag), Claudia IrinaBoldura, Oana-MariaMuntean, AlexandrinaBolibruchová, DanaMuntean, RaduBolshakov, AndreyMuntean, RoxanaBombac, DavidMuntean, SorinBompa, DanMunteanu, CorneliuBonadies, IreneMunteanu, Florentina-DanielaBončina, TonicaMur, JakaBondar, DaliMurab, SumitBondar, OleksandrMuraishi, ShinjiBondarev, Andrey V.Muralha, José DelgadoBondok, DoaaMurali, GunasekaranBonek, Mirosław S.Muramatsu, AtsushiBongale, ArunkumarMuramatsu, HiroyukiBonifacio, Maria A.Murata, KojiBonilla, Clara Eugenia PlazasMurata, MasaruBonilla-Cruz, JoséMuráth, SzabolcsBöning, KlausMurciano, AngelBonnekoh, CarstenMuro, MaiderBonomo, MatteoMurphy, AnthonyBonopera, MarcoMurray, Peter E.Bontempi, ElzaMurthy, Tammana Sree Rama ChandraBonyár, AttilaMurugan, SudharsonBoothello, RioMuruganandan, ShanmugamBopp, Fritz W.Murugasen, PriyaBorawski, AndrzejMurugesan, MohanrajBorca, CameliaMurzin, Serguei PetrovichBorchers, ChristineMusarrat, MuhammadBordeaşu, IlareMusgrave, Christopher Stephen AndrewBorderon, CarolineMusiał, JanuszBorhan, Adrian IulianMusic, DenisBorie, EduardoMussa, Mohamed H.Boris, BasokMussi, ValentinaBoris, RenataMustafa, EhtashamBormashenko, EdwardMustafaoglu, NurBorodi, GheorgheMustoe, George E.Borodianskiy, KonstantinMuszka, KrzysztofBorodin, ElijahMutalik, SrinivasBorodinas, SergejusMuthukrishnan, ShravanBorota, AnaMuthuramalingam, ThangarajBorowiec, JoannaMuthurasu, AlaganBorrás, AntoniMutlu, HaticeBortolotto, TissianaMutoh, YoshiharuBoruah, DibakorMutuma, BridgetBorugadda, Venu BabuMuzika, LukášBorysiuk, PiotrMuzzio, NicolásBorza, FirutaMyakalwar, Ashwin KumarBorzabadi-Farahani, AliMyalska, HannaBosacka, MonikaMyasoedova, Tatiana N.Boscornea, Aurelian CristianMyong Jae, YooBose, ArneshMyszograj, SylwiaBose, BipashaNa, JongbeomBose, SaurabhNabeel, MuhammadBoshkov, NikolaiNabiałek, MarcinBoskovic, IvanaNabil, SyedBosnar, SanjaNabochenko, OlgaBossu, MaurizioNachtane, MouradBostelmann, FriederikeNadimi, MohammadBôta, AttilaNadja, RohrBotelho, Joao T. S.Naeem, MuhammadBotha, IulianaNaffakh-Moosavy, HomamBotiz, IoanNagai, HirokiBotta, ChiaraNagalingam, Arun PrasanthBottaini, CarloNagaoka, ShojiBotti, SabinaNagaoka, ToruBotto, DanieleNagasawa, KazunoriBou, Santiago FerrandizNageswaran, ShubhaBouazaoui, MohamedNaghilou, AidaBoukhili, WalidNagira, TomoyaBoukhris, ImedNagit, GheorgheBoulaaras, SalahNagode, AlešBoulesbaa, AbdelazizNagórski, RomanBoulouz, AbdellahNagy, Janos B.Boumakis, IoannisNagy, MiklósBourauel, Christoph PNagy, TiborBourbon, GillesNaidu Raman, SudharsanBouropoulos, NikolaosNaik, Keerti M.Bousquet, ÉricNaik, PranjaliBouyanfif, HoussnyNair, Praful R.Bowman, MichaelNair, Sithara S.Boyatzis, StamatisNair, Sriramya D.Boytsova, OlgaNair, VaishakhBozkaya, OmerNaito, HidekiBr Babafemi lih, TomažNajem, JosephBrabec, LiborNaji, Abdul MuntaqimBrábek, JanNajman, StevoBracciali, AndreaNakamura, HiroyukiBracco, PierangiolaNakamura, NaokoBraconi, DanielaNakamura, YuzoBraeuninger, PhilippNakas, EnitaBraga, Maria-HelenaNakatani, AkihiroBragheri, FrancescaNakazawa, HidekiBragoni, AlbertoNakhaie, DavoodBrake, Nicholas A.Nalepka, KingaBrancato, VincenzaNalivaiko, Anton Yu.Branco, Fernando G.Nam, Il-WooBranco, JorgeNam, InhoBranco, RicardoNam, JeongsooBrand, Alexander S.Nam, Sang YongBrandi, FernandoNam, Seung YunBrando, GiuseppeNam, SeunghoonBrasiello, AntonioNam, Sung-chanBrauch, UweNambo, MasakazuBraun, MoritzNamdee, KatawutBraveenth, RamanaskandaNanduri, RavikanthBravo, TeresaNankya, RosalynnBrcic, MarinoNanot, SébastienBreakah, TamerNanukuttan, SreejithBreaz, Radu-EugenNäpänkangas, RitvaBrebu, MihaiNaphon, PaisarnBrechtl, JamiesonNapiórkowski, JerzyBredikhin, VladimirNaplocha, KrzysztofBreijo, Eduardo GarciaNapoli, ChristianBrekalo, IvanaNapolskii, KirillBreton, FrancisNaqash, Javed AhmedBrezinová, JanetteNarayanan, Remya P.Briatico-Vangosa, FrancescoNarciso, JavierBriciu-Burghina, CiprianNardecchia, FabioBrieger, JürgenNardo, Dario DiBrighi, MatteoNarita, FumioBrinkmann, Jorge Cortés-BretónNarkar, AmeyaBrintakis, KonstantinosNarkiewicz, UrszulaBriois, PascalNartova, Anna V.Brnić, JosipNaseri, EmadBroda, JanNasiłowska, BarbaraBroda, MagdalenaNasr, Mohammed SalahBrokmeier, Heinz-GünterNassar, HaniBrooks, Benjamin D.Nassef, AhmedBrostow, WitoldNastasa, ViorelBrotzu, AndreaNasti, GiuseppeBrouwers, JosNastulyavichus, AlenaBrouzgou, AngelikiNastuta, Andrei VasileBrown, Tim W. C.Natali Sora, IsabellaBrožová, SilvieNauber, RichardBrožovský, JiříNaushad, MuBru, DavidNautiyal, AmitBruna, PereNavakas, RobertasBruncko, JaroslavNavarro, Francisco JavierBrunčko, MihaelNavarro, RodrigoBrundage, Cord M.Navarro-Quezada, AndreaBrunel, DamienNaveed, MuhammadBruni, CarloNavlani, MiriamBrunklaus, GuntherNawani, PranavBruno, ElenaNawar, MahmoudBruno, GiovanniNawaz, AdnanBrus, ViktorNayak, SumeruBrutti, SergioNaydenkin, EugenyBrycki, BogumilNazarchuk, EvgenyBrylewski, TomaszNazarian-Samani, MasoudBrzeska, JoannaNazarov, Andrej P.Brzyski, PrzemysławNazarov, DenisBubnienė, UrtėNazir, AamerBuchinskaya, Irina I.Ndieyira, JosephBuchmeiser, Michael R.Neacsu, Ionela AndreeaBück, AndreasNeagoe, MirceaBuckner, Steven W.Neagu, AdrianBuczyński, PrzemysławNeamtu, Bogdan ViorelBudzik, GrzegorzNebreda, Jaime LazaroBuema, GabrielaNečas, DavidBueno, SalvadorNechifor, Aurelia CristinaBuffolo, MatteoNechifor, GheorgheBuganski, IreneuszNęcka, KrzysztofBugby, SarahNedelcu, DumitruBuhl, JohannesNedić, BogdanBui, Ngoc-TamNeduzha, LarysaBui, Thanh-TuânNeelakantan, PrasannaBuiu, OctavianNegrea, PetruBuj-Corral, IreneNegrutiu, CameliaBukiet, FrédéricNegrutiu, Meda-LaviniaBukovec, PeterNegut, IrinaBukreev, Dmitriy A.Neighbour, GarethBula, KarolNejad, Ali FarokhiBulai, GeorgianaNejman, MirosławBull, CraigNekouee, KhanBulushev, DmitriNekvindova, PavlaBūmanis, ĢirtsNelyubova, Viktoriya V.Bunaziv, IvanNemade, Pravin DinkarBunge, AlexanderNemati, Kamran M.Buntov, EvgenyNemes, Norbert M.Burak Irez, AlaeddinNemes, OvidiuBuravlev, IgorNémeth, PéterBurczyński, TadeuszNémeth, RobertBurduhos Nergis, Dumitru DoruNemeth, ZoltanBurduhos-Nergis, Diana-PetronelaNemova, DaryaBureš, RadovanNenadovic, SnezanaBurg, ArielaNeo, Dennis Wee KeongBurgaz, EnginNeplokh, VladimirBurinskienė, AurelijaNepomuceno, MiguelBürklein, SebastianNergis, Dumitru Doru BurduhosBurlacu, AdrianNerilli, FrancescaBurlakovs, JurisNesic, DobrilaBurlea-Schiopoiu, AdrianaNesmelov, DmitriyBurmistrov, KonstantinNesterov, DmytroBurrascano, PietroNesterova, Oksana V.Burwell, GregoryNestler, AndreasBusby, YanNethaji, DharmarasuBuskens, PascalNetskina, OlgaBusnaina, AhmedNeubauer, JürgenBustos, AlejandroNeugebauer, DorotaBusuioc, CristinaNeuhäuserová, MichaelaBut, Dmytro B.Neuman, Eric W.Butcher, CliffordNeuner, MatthiasButkute, RenataNeupane, DipeshButoi, BogdanNeves, Cristina Sofia Dos SantosButruk-Raszeja, BeataNeves, JoséButt, M. AliNeves, Marcia C.Butu, AlinaNevřivová, LenkaBuyle-Bodin, FrançoisNevshupa, RomanBüyük, Süleyman KutalmışNevskii, SergeiBuzolin, Ricardo H.Ng, Pui-LamByliński, HubertNg, Wei LongBylya, OlgaNgai, ToByun, Soo-HwanNge, Thi ThiBywalski, CzesławNgene, PeterC. Castro, Ana MeriaNgeow, Wei CheongCabaleiro, DavidNgo, Chi-VinhCabalova, IvetaNgo, Tri-DungCaban, JacekNguyen Hoang, VietCabanelas Valcárcel, Juan CarlosNguyen Quang, ChinhCabboi, AlessandroNguyen, Col ChiCabeza Simo, MartaNguyen, Hanh ThuyCabeza, AurelioNguyen, KhanhCabezas, M. GuadalupeNguyen, NhuĆabov, TomislavNguyen, Son ThanhCabrera-Barjas, GustavoNguyen, Sy-NgocCabrera-Sierra, Román A.Nguyen, Thanh TrungCābulis, UģisNguyen, Van-ChucCada, MartinNguyen-Manh, DucCadar, OanaNguyen-Thanh, NhonCafaro, FrancescoNi, HongyangCaggiano, AntonioNi, PingheCai, JizheNi, QingCai, ShuoweiNi, RongCaiazzo, FabriziaNi, XinchenCaillol, SylvainNica, CristinaCajka, RadimNicetin, MilicaCakmak, UmutNicoara, AdrianCalabrò, Paolo S.Nicoara, MirceaCalafel, ItxasoNicolai, JulienCaldas, PauloNicolas, LebonCalero-Castillo, Ana IsabelNicolau, Dan V.Calì, MicheleNicolaus, MartinCaliebe, WolfgangNicolescu, Cristina MihaelaCalisi, NicolaNicolicescu, ClaudiuCalixto, SergioNiculae, DanaCałka, AndrzejNiculescu, VioletaCalniceanu, HoriaNie, BinjianCaltagirone, ClaudiaNie, ChuanxiongCalvio, CinziaNie, JunCalzada, Mónica PreciadoNieckarz, DamianCalzaferri, GionNiedoba, TomaszCamacho, JoaquinNiedostatkiewicz, MaciejCamara Serrano, JuanNiemczak, MichalCamelia, GaborNiemczewska-Wójcik, MagdalenaCameron, DavidNiemczyk, AgataCameron, JosephNieminen, KaarloCamin, BettinaNiessen, FrankCammarata, AntonioNiewczas, Agata M.Campagnolo, AlbertoNiewiadomski, PawełCampana, FrancescaNiitsu Campo, KaioCampbell, JohnNikam, SagarCampbell, Kris A.Nikishin, SergeyCampello-Vicente, HectorNikitas, NikolaosCampian, Cristina MihaelaNikitin, DaniilCampilho, Raul D. S. G.Nikodem, AnnaCampillo, JoxemiNikolaev, AlexanderCamposo Pereira, ArturNikolaevskii, Stanislav A.Can, AhmetNikolaidis, Alexandros K.Canale, ClaudioNikoleli, Dimitrios P.Canale, LaurentNikolett, KállaiCañamares, Maria VegaNikolić, NebojšaCañavate, JavierNikolić, Ružica R.Candamano, SebastianoNikolić, Vladimir B.Candelario, Victor ManuelNikolopoulos, ChristosCandiani, GabrieleNikoloudaki, GeorgiaCandolfi, ChristopheNikolova, MariaCanela-Garayoa, RamonNikravech, MehrdadCanetta, ElisabettaNilges, TomCaniato, MarcoNina, DiakonovaCanivell, JacintoNinan, NeethuCannone Falchetto, AugustoNingrum, EvaCano-Crespo, RafaelNirschl, HermannCantera, M. AsunNishibori, EijiCantergiani, ElisaNishiguchi, NorihikoCantisani, GiuseppeNishii, YasushiCao, FaheNishimoto, YoshioCao, HongdaNishinaga, TohruCao, Hua-TangNishitsuji, ShotaroCao, ShoufanNishiwaki, TomoyaCao, ShuaiNishizawa, TatsuoCao, XiaoshanNitti, GiuseppeCao, YingNitulescu, GeorgianaCapasso, IlariaNitulescu, MihaiCapasso, RaffaeleNiu, JinboCapezza, AntonioNiyas, HakeemCapitão, Silvino DiasNocca, GiuseppinaCaponio, FrancescoNocchetti, MorenaCaporali, StefanoNocivin, AnnaCapparè, PaoloNocke, AndreasCăprărescu, SimonaNoda, RyuCaputo, PaolinoNoei, HeshmatCara, EleonoraNogueira, Paulo JorgeCarabineiro, SóniaNoh, HeesoCaramês, JoãoNoirot, JeanCarames, João Manuel MendesNomoto, SukeharuCaratozzolo, MarianoNoor E Khuda, SarkarCarazeanu Popovici, IonelaNoor, NuruzzamanCárcel-Carrrasco, Francisco JavierNooraiepour, MohammadCarda Castelló, Juan B.Noorani, Rafiqul I.Cardea, StefanoNoori, Md TabishCardenes, VictorNordblad, PerCardwell, DavidNorek, MałgorzataCarla, QueirósNorkus, EugenijusCarleer, RobertNorrish, JohnCarli, StefanoNorton, Amie E.Carlino, ElvioNorval, GraemeCarlone, PierpaoloNoskov, Roman E.Carlucci, ClaudiaNotario-Pérez, FernandoCarmali, SheilizaNotthoff, ChristianCarmen, MateescuNovak, BalthasarCarmen-Cătălina, RusuNovák, JosefCarmona Marques, PedroNovak, NejcCarneiro, Olga SousaNovák, PavelCarneiro, VitorNovakovic, Jasmina GrbovicCaron, ArnaudNovakovic, RadaCarone, Benjamin R.Novikov, ValentinCarossa, MassimoNovitskii, A. P.Carou, DiegoNowacka, MariaCarrado, AdeleNowacki, KrzysztofCarragher, JohnNowaczyk, JacekCarrasco-Marin, FranciscoNowak, Agnieszka J.Carré, VincentNowak, KrzysztofCarrilho, EuniceNowak, PawelCarrilho, Rui M. B.Nowak, RadosławCarter, Richard M.Nowak, WojciechCarucci, CristinaNowicki, WaldemarCarullo, GabrieleNowrot, AndrzejCaruso, SerafinoNozaki, KosukeCarvalho, IsabelNtintakis, IoannisCarvalho, PaulaNuić, IvonaCarvalho, SilviaNunes, Cristiana LaraCarvelli, ValterNuñez, NeftaliCasa, MarcelloNunna, Bharath BabuCasagrande, AngeloNuţǎ, Diana CameliaCasagrande, CézarNygårds, MikaelCasalotti, ArnaldoNykyforchyn, HryhoriyCasapulla, ClaudiaNys, IngeCasarejos, EnriqueO′Duill, SeanCasarin, RobertoOancea, FlorinCascardi, AlessioOancea, GheorgheCaschera, DanielaObande, WinifredCasellas, DanielObeid, MuhannadCaseri, WalterObeid, ObeidCasey, Philip S.Obhođaš, JasminaCasiello, MicheleOblak, ČedomirCassagnau, PhilippeObradovic, Nina N.Cassano, DomenicoObraztsov, Alexander N.Cassone, GiuseppeObreja, Alexandru CosminCastaldi, GiuseppeObreja, Serban GeorgicaCastaldo, PaoloOda, TakujiCastaneda, HomeroOdintsova, GalinaCastañón, Ana MaríaOdrobiňák, JaroslavCastedo, RicardoOehlschlaeger, MattCastellano, Nuria NovasOezkan, SeldaCastellari, MassimoOfuyatan, Olatokunbo M.Castillo, GermanOgale, Amod A.Castillo-Rodríguez, G. A.Ogam, ErickCastro Alves, RicardoOgawa, KazuyoshiCastro Cerda, Felipe M.Ogawa, ToshioCastro, AndreOggeri, ClaudioCastro, FernandoOgino, YoichiroCastro, Ítalo B.Oh, DaekyunCastro-Colin, MiguelOh, DanielCastrodeza, Enrique MarianoOh, Ji-HyeonCataldi, AmeliaOh, JonghyunCatapano, AnitaOh, Jong-MinCatapano, SantoOh, JuwonCatarina Mendes, JoanaOh, Sang-KeunCatizzone, EnricoOh, SeunghanCatledge, Shane AaronOh, SoramCatros, SylvainOh, TaekeunCavagnetto, DavideOh, Tae-SikCavalcanti, Marcos Fernando Xisto BragaOh, TeresaCavallaro, GiuseppeOh, Won ChunCavalli, MatthewOh, Won-SuckCavalli, Matthew Matthew N.Ohba, SeigoCavallo, CarmenOhtsuka, TakashiCavillon, MaximeOikonomou, ArtemiosCazacu, AnaOikonomou, Evdokia K.Cazorla-Luna, RaúlOjha, Gunendra PrasadCazzanelli, MassimoOjwang, DicksonCea Mingueza, PilarOk, Kang MinCebríán-Carretero, José LuisOka, KengoCedervall, JohanOkada, ShujiCedillo-González, Erika IvethOkada, TakaoCegan, TomasOkamoto, MasamiĆelepirović, NevenkaOkamoto, MotokiĆelić, RobertOkamoto, RobertaÇelikten, SerhatOkano, ShigetakaCepeda, JavierOkazaki, JojiCerbu, CameliaOkazaki, ToshiyaCereceda, DavidOkhay, Olena I.Ceretti, MonicaOkiji, TakashiČermák, JanOkoro, Oseweuba ValentineCerrato, ErikOkoya, Samuel SegunCerrato, Giuseppina PinucciaOkoye, Ugochukwu PatrickCerro-Prada, ElenaOkpin, NaCeruti, AlessandroOkulov, IlyaCervelló, Gemma GutiérrezOkunkova, Anna A.Cervenka, JanOladapo, BankoleČervenka, MilanOlariu, Marius-AndreiCervino, GabrieleOlaru, DumitruCesano, FedericoOlayiwola, SaheedCésar Pereira, ArnaldoOlcum, MelisCesarz-Andraczke, KatarzynaOlejnik, EwaÇetin, Arif EnginOlejnik, KonradCha, ChaenyungOleksik, MihaelaCha, Sang-HoOleksik, ValentinCha, Sung WoonOlenin, Andrei YuCha, WonsukOleszak, DariuszChabi, SakinehOlivares-Robles, Miguel AngelChaboussant, GregoryOliveira, FernandoChacko, Jenu VargheseOliveira, João PedroChae, MichaelOliveira, Karla Mychellyne CostaChae, SoosangOliveira, MonicaChai, Wai SiongOliveira, Serafim M.Chai, Wen LinOliveira, VítorChaitanya, SaurabhOliver, Jeremie D.Chaiyasarn, KrisadaOliver, ZemanChakrabarti, AmartyaOliviero Rossi, CesareChakrabarty, RohanOlivito, Renato SanteChakraborty, JoyrajOller, EvaChakraborty, Shitanshu ShekharOlmos, DaniaChalas, RenataOlsson, RickardChaldyshev, VladimirOman, SimonChalifoux, BrandonOmar, AhmedChalioris, ConstantinOmar, Hend AliChalkias, Dimitris A.Omenat, AnaChałupnik, StanisławOmine, KiyoshiChampagne, BenoîtOmiya, MasakiChan, AlexOnbaşlı, Mehmet CengizChan, Chi WaiOndřej, KováříkChan, Yau KeiOnesto, ValentinaChanda, AvishekOng, Dennis C.Chandel, Arvind SinghOng, Dominic E.L.Chandran Suja, VineethOnisor, FlorinChandrasekaran, Srinivas NiranjOnoda, MasaruChang, Cheng-Hsun-TonyOnuaguluchi, ObinnaChang, Chia MingOnyelowe, Kennedy ChibuzorChang, Chia-ChenOnys′ko, PetroChang, Der-WenOpálek, AndrejChang, DingranOpel, OliverChang, Hai-ChouOpěla, PetrChang, Han-WeiOpoka-Winiarska, ViolettaChang, Hao-HuengOpra, DenisChang, HyejinOprea, OvidiuChang, Jen-RayOptasanu, VirgilChang, Jui-ChengOpydo-Szymaczek, JustynaChang, MinwooOralová, VeronikaChang, Po-HsiangOrazbayev, BakhtiyarChang, SeChinOrbulov, ImreChang, Sheng-PoOrbulov, Imre NorbertChang, Shih-YingOreshonkov, AleksandrChang, WenlongOrešković, MarkoChang, Yuan-JenOrinak, AndrejChantarak, SirinyaOriňáková, RenátaChao, Chih-YehOrion, ItzhakChapala, PavelOrlowsky, JeanetteChapelle, FrédéricOrnek, MetinChappel, EricOrosz, TamasCharalampakos, VassiliosOrsini, AndreaCharalampous, PaschalisOrsini, GiovannaCharbonnier, BaptisteOrtega, José MarcosCharfi, AmineOrtega, JosuCharisiou, NikolaosOrtega, Miguel ACharvatova, HanaOrtigosa, RogelioChatterjee, AtanuOrtiz Miranda, Annette SuleikaChatterjee, SabornieOrtyl, JoannaChattopadhyaya, SomnathOsajima, Josy AnteveliChaturvedi, MayankOsakoo, NattawutChau, Kwok-wingOscurato, Stefano LuigiChaudhari, RakeshOsella, SilvioChaudhuri, Sandeep K.Oshikiri, TomoyaChauhan, AnkurOshman, ChristopherChavali, MurthyOsial, MagdalenaChe Halin, Dewi SuriyaniOsinski, PiotrCheah, Chee BanOsipov, AndreyChecchi, VittorioOskay, Rıza GörkemChee, Pei SongOsman, NafisahChee, See WeeOsonga, Francis JCheepu, Murali MohanOsório, Wislei R.Chehroudi, BabakOsswald, TimCheikh, Khadija EIØstergaard, Martin B.Chekhonin, PaulOstos Marcos, Francisco JoséChelariu, RomeuOstovan, FarhadChen, Bing-HungOsuch, PiotrChen, BumingOsuch-Słomka, EdytaChen, ChanghaiOtegui, Jose LuisChen, Cheng-ChenOtero, VanessaChen, Cheng-HoOthman, Nur HidayatiChen, ChiachungOtsuki, AkiraChen, Chia-YunOtto, Daniel P.Chen, Chien-ChangOu, ZihaoChen, ChunlinOuasri, AliChen, DazhengOuellet-Plamondon, ClaudianeChen, EnguoOulego, PaulaChen, FengOuvrard, AimericChen, GuoOuyang, JianChen, HailanOwari, MasakiChen, HaoOwen, JoshuaChen, Hone-ZernOwolabi, GbadeboChen, HsiangOwsiński, RobertChen, HuixiangOzaltin, KadirChen, Hung-ShingOzawa, YoshikiChen, I-Wen PeterÖzer, GökhanChen, Jer-MingOzerov, MaximChen, JiahaoOzerova, Anna M.Chen, Jian-ZhangOzga, AndrewChen, JihuaOzgurluk, YasinChen, JingOzherelkov, DmitriyChen, Kuan-RenOzkan, GokcekayaChen, KuanyuOzlu, EkremChen, LeiOzmaian, MasoumehChen, Lung-ChienPabon, Juan J.G.Chen, MengyuanPabst, AndreasChen, Ming-songPachamuthu, Muthusamy PoomalaiChen, MingzhouPacheco, JoãoChen, Pang-ShiuPacheco, NeithChen, PeiPacheco, Rafael RochaChen, PengPacifici, AndreaChen, Po-HsunPacifici, LucianoChen, Po-WenPaciorek-Sadowska, JoannaChen, RongchuangPacios, MerceChen, ShangzhiPacławski, AdamChen, Shao-HsienPacurar, Razvan IoanChen, Shia-ChungPaczesny, JanChen, ShikunPączkowski, PrzemysławChen, Shong-LoongPaczos, PiotrChen, ShujunPadilla, JavierChen, ShuoPadmanabhan, Sanosh KunjalukkalChen, ShutingPadnya, PavelChen, SiPadrão, JorgeChen, Tai-ChengPagacz, JoannaChen, Tao-HsingPagano, CinziaChen, Tei-ChenPagano, RosannaChen, Tony Lin-WeiPagano, StefanoChen, WeiPagliarulo, VitoChen, WeijianPagnotta, StefanoChen, Wen-ChengPahuja, RishiChen, Wen-JauhPaiè, PetraChen, Yi-ChunPaik, TaejongChen, Ying-LiangPaiva, Ana PaulaChen, YuPaiva, DianaChenet, TatianaPak, A. YaCheng, Chia-ChiPak, ChanhoCheng, HaiziPakieła, WojciechCheng, Hsu-HuiPal, JitendraCheng, HuanyuPal, NabamitaCheng, HuiPal, SnehashisCheng, JiePalacio Gómez, Carlos AndrésCheng, JipingPalacios-Santander, José MaríaCheng, JunyePaladini, GiuseppeCheng, Kuo-BingPalamà, Ilaria Elena LenaCheng, XinPalazzatti, FedericoChenrayan, SenthilPalchoudhury, SoubantikaCheon, JasonPalella, AlessandraChepel, VitalyPalenzuela, José AntonioChepurnenko, AntonPaleu, ViorelCheraghian, GoshtaspPalevicius, ArvydasCherevan, AlexeyPalin, DamianCherng, Juin-HongPałka, ŁukaszCherng, Jyh-ShiarnPallandre, AntoineChernyak, SergeiPalma, Paulo J.Chernyshov, MikhailPalma-Dibb, Regina GuenkaCherpakov, AlexanderPalmieri, Frank L.Chersich, SilviaPaltanea, GheorgheCherunova, IrinaPaluch, Andrew S.Chethan, K. N.Palumbo, DavideCheung, Gary Shun PanPalumbo, GaetanoChew, Khian-HooiPalumbo, OrieleChi, FengPamies, RamonChi, TengPamin, JerzyChiang, Chih-ShengPampaloni, FrancescoChiang, Chin LungPan, AnlianChiang, Tzu HsuanPan, Chi-LingChiang, WeihungPan, FeiChiappim, WilliamPan, Guan-TingChiarella, EmanuelaPan, HouwenChiarini, MarcoPan, MatthewChibac-Scutaru, Andreea L.Pan, Po-shenChicardi, ErnestoPan, QuanwenChicet, DanielaPan, RuijunChien, Hsiu-WenPana, IulianChiessi, EsterPanaccione, GiancarloChilimoniuk, PaulinaPanagiotopoulou, AngelikiChilkoor, GovindPanayiotou, CostasChillura Martino, DeliaPanchal, SatyamChinakhov, DmitryPanchenko, OlegChinh, Nguyen DucPańcikiewicz, KrzysztofChiniforush, Alireza A.Panda, BiranchiChinnathambi, ShanmugavelPandele, MadalinaChioibasu, DianaPandey, Alok KumarChiou, JiachiPandey, PuranChiozzi, AndreaPandey, RaviChirayath, Varghese AntoPandíțh, AnúpChircov, CristinaPanek, JaroslawChiriu, DanielePánek, MilošChistyakov, EvgeniyPang, ShengyongChiu, Justin N.W.Panin, SergeyChiu, Sheng-YiPanjan, PeterChiulan, IoanaPankratov, DenisChladek, GrzegorzPankratov, VladimirChladil, JosefPanoutsakopoulos, VassiliosChlanda, AdrianPanowicz, RobertChmelik, FrantisekPant, BishweshwarChmelko, VladimirPantazopoulos, GeorgeChmielarz, PawełPantelic, IvanaChmielewski, RyszardPantić, MilicaChmielewski, TomaszPanunzi, BarbaraCho, Byung JinPanzarasa, GuidoCho, ChiajungPaolantonio, MicheleCho, Dae-HyunPaolini, ValerioCho, Hak DongPaolino, MarcoCho, Hea-YoungPaolone, AnnalisaCho, JaehyukPaolone, GaetanoCho, Jong-RaePapacharalampopoulos, AlexiosCho, Jung SangPapadaki, AikateriniCho, KihoPapadaki, EugeniaCho, Nak-KyunPapadakis, RaffaelloCho, NamchulPapadogiannis, NektariosCho, Sang-ChoonPapadopoulos, Antonios N.Cho, So-HyePapadopoulou, AlexandraCho, Sung OhPapagiannopoulos, George A.Cho, Sung WoonPapakonstantinou, ChristosChodon, RafalPapalou, AngelikiChodun, RafalPapanikolaou, StefanosChoe, Han CheolPapanikos, ParaskevasChoh, In-ChulPapathanasiou, Thanasis D.Choi, DongukPapatzani, StylianiChoi, Jae HoonPapavasileiou, Georgios S.Choi, Jae-HongPapazi, AikateriniChoi, Jae-YoungPapazoglou, Emmanouil LazarosChoi, Joon-SungPape, FlorianChoi, KihwanPapenberg, NikolausChoi, Kwang-SeongPapi, PieroChoi, Kwang-YongPapia, EvaggeliaChoi, Kyeong-KeunPapoulis, DimitriosChoi, Kyu YongPappalettera, GiovanniChoi, MingiPappas, ChristosChoi, Nag JungPapuga, JanChoi, Seung-bokPapurello, DavideChoi, Seung-hoPar, MatejChokkalingam, AnandParajo, Juan JoséCholakova, DianaPáramo-García, UlisesChong, Parskson Lee GauParande, GururajChong, Song-HunParanjayee, MandalChong, Woon ChanParaschiv, GigelChoo, Thye FooParastoo, JamshidiChotkowski, MaciejPardanaud, CédricChou, Hung-LungParedes, José PabloChoudhary, HemantParedes-Madrid, LeonelChoudhuri, IndraniPareja, Rafael BravoChougan, MehdiPargar, FarhadChoung, JoonmoParia, DebadritaChow, James C LParia, SarbaranjanChowdhury, A. S. M. RaufurPariente, Cristóbal GarcíaChrcanovic, BrunoParikh, PriteshChristensen, Jørn BolstadParinov, Ivan A.Christoforidis, KonstantinosPark, ByoungsunChristopoulos, ThomasPark, Chan-WooChristova, DarinkaPark, Dae-HwanChrobak, ArturPark, DaewookChronakis, Ioannis S.Park, Deok-YongChronopoulos, DimitriosPark, Eun DuckChu, Seok JaePark, Eun-JinChuah, Chong YangPark, IlhwanChuchala, DanielPark, Jeong WonChudoba, DorotaPark, Jeong YoungChudzikiewicz, AndrzejPark, JihoonChuirazzi, WilliamPark, JinsungChukhlanov, Vladimir YurievichPark, Ji-SangChumaevskii, AndreyPark, Ji-WonChung, Hyun-JoongPark, Jong-SeokChung, Koo-HyunPark, Jong-WonChung, Roy Byung KyuPark, Joon SikChung, SeungjunPark, Jun-BeomChung, Sheng-HengPark, JungChurakova, AnnaPark, Keum HwanChurilov, Grigory N.Park, KwangsukChuryumov, Alexander YuPark, Min SooChuvil’deev, Vladimir NikolaevichPark, Min-SikChwastek, KrzysztofPark, SangkiChwojnowski, AndrzejPark, SangmoonChworos, ArkadiuszPark, SangwonChylińska, MartaPark, Seong-JikCiampi, SimonePark, Seung KooCiardelli, FrancescoPark, SolmoiCicala, GianlucaPark, Sung-JuCiccarese, FrancescoPark, SungjunCicchi, StefanoPark, Sung-SooCicero, SergioPark, Young-TaeCicone, TraianParker, JonathanCieśla, JolantaParlatescu, IoaninaCieslar, MiroslavParmar, VinodCieślik, BartłomiejParmeggiani, MatteoCieślik, IwonaParr, ChristopherCifrian, EvaParra Arévalo, María IsabelCiftja, OrionParravicini, JacopoCiftyürek, EnginParry, AndrewCihangir, FerdiParth, PanchmatiaČikoš, ŠtefanParthiban, KathirvelCimalla, VolkerPartovi-Azar, PouyaCimpean, AnisoaraPartovi-Nia, RachelCimpoesu, FanicaPartyka, JanuszCimpoesu, NicanorPašák, MatejCimpoeşu, RamonaPasayat, Shubhra S.Cinelli, PatriziaPashchenko, DmitryCiobanu, GabrielaPasini, DarioCiobanu, MadalinaPaslawski, JerzyCiocan, Lucian TomaPasquale, StefaniaCiocoiu, Robert CatalinPasseri, DanieleCiolacu, DianaPasta, GianluigiCiolacu, FlorinPasuk, IulianaCiorap, RaduPaszkiewicz, ReginaCippo, Enrico PerelliPaszkowski, RobertCiria, MiguelPatachia, SilviaĆirić, AleksandarPatanè, SalvatoreCirillo, GiuseppePatcharapit, PromoppatumCirisano, FrancescaPatel, Dinesh KumarCirtoaje, CristinaPatel, DipakCirule, DacePatel, JigneshkumarCiszewski, MateuszPatel, Kapil D.Ciszewski, Wojciech MichałPatel, RaviCiuffi, KatiaPatel, VivekClark, CasparPatella, BernardoClassen, EdithPaternò, Giuseppe MariaClausen, BrigittePaternoster, CarloClegg, John R.Paterson, ThomasClematis, DavidePatil, ArunClemens, FrankPatil, DeepakClement, YuenPatil, SandeepClevers, SimonPatil, Swati J.Cobianu, CornelPatmonoaji, AnindityoCobo, CarlosPatra, JagabandhuCoclite, Anna MariaPatrício, Sónia G.Coco, SilverioPatroi, DeliaCodrean, CosminPatsios, SotirisCoduri, MauroPattappa, GirishCoelho, AnaPatterson, Albert E.Coelho, JoãoPatterson, Eric ACoffetti, DennyPatterson, JenniferČohodar Husić, MaidaPatti, AntonellaCojocaru, AncaPatyk, RadosławCojocaru, CorneliuPauk, JolantaCojocaru, VasilePaul, BereColangelo, FrancescoPaul, GeoColangelo, GianpieroPaul, Partha P.Colella, AbnerPaul, RajibColeman, NicholaPaul, SanjoyColin, JeromePaul, Suvash ChandraColla, ValentinaPaul, TanmoyCollar, Emilia P.Paulish, Andrey GeorgievichCollart-Dutilleul, Pierre-YvesPaulo, SiriCollings, Peter J.Paunoiu, ViorelCollins, MauricePavel, Ileana AlexandraColmati, FlávioPavic, KristinaColombo, JohnPavic, ValentinaČolović, Mirjana BPavlík, ZbyšekColusso, ElenaPavlin, MaticComan, PaulPavliňáková, VeronikaComanescu, CezarPavlov, Igor S.Comesaña, RafaelPaw, MilenaComor, MirjanaPawar, NitinConan, FrancoisePawbake, AmitConcilio, AntonioPaweł, KłosowskiConnolly, James P.Paweł, WiśniewskiConserva, EnricoPawlik, JanConsolo, UgoPawłowska, AgnieszkaConstantin, Gabriel-AlexandruPawłowska, BarbaraConstantin, GeorgePawluczuk, EdytaConstantin, Oana EmiliaPawlus, SebastianConstantinescu, Catalin-DanielPawyta, MiroslawaConstantinescu, Dan MihaiPayne, DanielConstantinescu, HoriaPayne, KarlConstantinescu-Aruxandei, DianaPaz Hernandez, RubénConstantinides, ChristosPazderka, JiříConte, RominaPazniak, HannaConway, BarbaraPearce, JoshuaCooke, MeganPecci Lloret, María PilarCoppola, BartolomeoPecci-Lloret, Miguel RamónCoppola, NunziaPecharromán, CarlosCorbu, Ofelia CorneliaPecherski, RyszardĆorić, DankoPech-May, Nelson WilburCorigliano, PasqualinoPeciuliene, VytauteCoronado, JohnPecqueur, SébastienCoronado, Juan M.Pedarla, AravindCorrea, Cinthia AntunesPeddapuram, AdithyaCorrea, DavidPedersen, HenrikCorrêa, EdmilsonPedone, DeborahCorregidor, VictoriaPedretti, DanieleCorreia, Victor FrancoPedullà, EugenioCorrente, Giuseppina AnnaPeer, PetraCorsaro, CarmeloPeera, Shaik GouseCortaberria, GalderPeeters, DaniëlCortese, BarbaraPeiffer, HermanCortese, MartaPeiris, DinithiCoseri, SergiuPeixinho, Nuno Ricardo MaiaCosma, PinalysaPejčić, Ana S.Cosoveanu, AndreeaPejić, Biljana M.Costa, HugoPelin, CristinaCosta, Josiel MartinsPellegrino, GerardoCosta, Rui S.Pelletier, J.M.Costafreda, Jorge LuisPelliccioni, Gian AndreaCosta-Kramer, José LuisPellicer, José AntonioCostan, Victor-VladPeluso, AndreaCostantino, FerdinandoPeña, Jose IgnacioCostanza, GirolamoPeña-Garcia, R.Costa-Pinto, Ana RitaPeñalver, RosaCosta-Vera, CesarPeña-Pitarch, EstebanCostica, BejinariuPencea, IonCotas, CarlaPenchal Reddy, MatliCoteata, MargaretaPenchev, HristoCowan, Ellen A.Penchev, PavelCraciun, Eduard-MariusPeng, DiCraciunescu, IzabellPeng, GuangjianCraeye, BartPeng, Tzu-YuCraft, Aaron EPennisi, RosamariaCraft, AndrewPenttilä, Paavo A.Cretu, SpiridonPepelnjak, TomazCreylman, VeerlePeral, AngelCriado-Gonzalez, MiryamPerale, GiuseppeCrisan, AndreiPerec, AndrzejCrisan, Gloria CeraselaPereira, A.M.Crisp, Ryan W.Pereira, AlejandroCristache, Corina MarilenaPereira, AndréCristea, DanPereira, DoloresCristian, PetcuPereira, Fernando J.Cristóbal, JuliaPereira, Manuel Francisco CostaCritchley, KevinPereira, OctavioCrivellaro, AlanPereira, RubenCroce, PietroPereira, RuiCroitor, LiliaPérez Puyana, Víctor ManuelCrotti, ElenaPerez Taborda, Jaime AndresCrucho, JoãoPerez, CarmenCrupi, VincenzaPérez, GloriaCruz, SandraPerez-Alfaro, IreneCruz-Delgado, Víctor JavierPerez-Alvarez, JonatanCsaba, TothPérez-Soriano, Eva M.Csaki, IoanaPergher, Sibele B.C.Csaki, StefanPeric, TamaraCsapo, EditPérigo, Elio AlbertoCsete, MáriaPerini, PaoloCsík, AttilaPeriwal, PriyankaCsoka, LeventePeriyasamy, Aravin PrinceCtibor, PavelPerkowski, DariuszCuan-Urquizo, EnriquePerna, Alessia SerenaCuberes, TeresaPernas, JesusCuesta, RafaelPerniu, DanaCuevas, FranciscoPeroš, KristinaCuevas-Suárez, CarlosPerov, NikolaiCui, MinmingPerrella, MicheleCui, QingyuPerrin, JonathanCui, WeiPerrot, ArnaudCui, WenyuanPersson, AxelĆuković, SašaPerteghella, SaraCulic, BogdanPerumal, SugunaCunha, EunicePervaiz, SalmanCunha, EvaPerveen, AsmaCurély, JacquesPessoa, Rodrigo SávioCurry, NicholasPestov, AlexanderCurto, AdrianPetcu, CristianCurulli, AntonellaPetean, IoanCustídio, JoãoPetelin, AndrejCutright, Teresa J.Peter Amalathas, AmalrajCvek, MartinPéter, BarkóczyCwalina, BeataPeterka, JozefĆwikła, GrzegorzPetit, ChristopheCwudziński, AdamPetkovšek, RokCygan, TomaszPetošić, AntonioCyprych, KonradPetr, MynarcikCyran, KatarzynaPetrak, SlavenkaCysewska, KarolinaPetrak, VaclavCzaja, PawełPetrakis, EvangelosCzapik, AgnieszkaPetrakli, FotiniCzapik, PrzemysławPetráková, VladimíraCzaplewski, CezaryPetrarca, CarloCzarnecka-Komorowska, DorotaPetrescu, EmilCzarnecki, SlawomirPetrescu, Florian Ion TiberiuCzechowska, JoannaPetrescu, ValentinCzekanski, AleksanderPetridis, CostasCzeppe, TomaszPetriev, IliyaCzernek, JiříPetrik, PeterCzerwinski, FrankPetrila, IulianCzerwiński, MichalPetrini, MorenaCzinege, ImrePetritoli, EnricoCzujko, TomaszPetrone, GiuseppeCzupryński, ArturPetrou, MichaelD’Amario, MaurizioPetrounias, PetrosD’Angela, DaniloPetrov, RoumenD’Orazio, FrancoPetrov, Vladimir G.D’orsi, RosaritaPetrov, Vladislav A.D′Addazio, GianmariaPetrović, JelenaD′Agostino, CarminePetrovich, Andryushin KonstantinD′amore, MatteoPetru, JanaD′Andrea, AntonioPetru, MichalD′Elia, AlessandroPetruccelli, RaffaellaDa Costa, AndréPetrucci, RobertoDa Silva Candal, AndrésPetrus, JosefDa Silva, Eduardo MoreiraPetrus, MateuszDa Silva, João PiresPetryshynets, IvanDa Silva, Marcio BacciPettarin, ValeriaDa Silva, Pedro RaposeiroPetuhov, OlegDa Venda Oliveira, Paulo JoséPetukhov, Andrei V.Dabos-Seignon, SylviePetunin, Aleksandr A.Dąbrowski, MariuszPetykiewicz, JanDąbrowski, WojciechPeveler, WilliamDadgar, ArminPezda, JacekDaghbouj, NabilPfaendner, RudolfDahal, JibaPfnür, HerbertDahash, AbdulrahmanPfriem, AlexanderDahl, Jon EinarPhadtare, VarshaDahle, SebastianPham Truong, Thuan NguyenDai, BaominPham, Ngoc DuyDai, ShengPhan, Thi Tuong VyDai, ZhengfeiPhanthong, PatchiyaDal Palù, DorianaPhilipsen, HaroldDal Piva, AmandaPhillippa, BronsonDalal, JasvirPhillips, ChrisDalaq, Ahmed S.Phua, Eric J.R.Dalena, FrancescoPhung, Quoc TriDalessandri, DomenicoPi, TeresaDalibón, Eugenia L.Piao, JinhuaDalkiranis, Gustavo GonçalvesPiasecki, AdamDalverny, OlivierPiattelli, AdrianoDama, MuraliPiatti, ErikDÁmato, RobertoPicazo, ÁlvaroDambrauskas, TadasPicca, Rosaria AnnaDamjanović, DarkoPiccardo, PaoloDammaschke, TillPiccininni, AntonioDana Adriana, Ilutiu-VarvaraPichandi, SubramaniDancette, SylvainPicollo, FedericoDaneshazarian, RezaPicoux, BenoitDaniel-da-Silva, Ana LuísaPieczyrak, BarbaraDaniele, StephanePiedade, Ana PaulaDanihelová, AnnaPiekarczuk, ArturDanko, MartinPiekarczyk, AdamDante, SilviaPielichowski, KrzysztofDao, Van-DuongPiepiórka-Stepuk, JoannaDaoush, Walid M.Piergies, NataliaDarabantu, MirceaPierini, FilippoDarapaneni, PragathiPiesowicz, ElżbietaDarder, MargaritaPietras, DanielDarnowski, PiotrPietrikova, AlenaDaroonparvar, MohammadrezaPietrowicz, SlawomirDas, AbhishekPietrusiak, DamianDas, Gour MohanPietrusiewicz, PawelDas, HrishikeshPietruszka, BarbaraDas, Proloy TaranPietrzak, Tomasz KarolDas, SudhansuPietrzyk, BozenaDas, Tapan KumarPietrzyk, StanislawDasari, AravindPietrzyk, StanisławDash, BirajaPietschnig, RudolfDassios, Konstantinos G.Pigliacelli, ClaudiaDastgeer, GhulamPilarczyk, KacperDaszykowski, MichałPilarczyk, WirginiaDatt, RamPilch-Pitera, BarbaraDauth, StephaniePilic, BrankaDavidova, Galina A.Pilicheva, BisseraDavidová, MariePilipović, AnaDavies, ChrisPillai, Rajalekshmi C.Davim, J. PauloPilo, RaphaelDavis, FredPiloto, PauloDavis, PaulPiluso, SusannaDavletshin, ArturPimenov, DanilDawes, JudithPimentel, CarlosDawidowski, WojciechPin, ChristopheDawoud, BelalPiña Ramirez, CarolinaDe Aguiar, José BarrosoPina, JoãoDe Almeida, Jose Carlos MartinsPinakoulaki, EftychiaDe Almeida, José Manuel Marques MartinsPinargote Nestor Washington, SolisDe Angelis, FrancescoPinca-Bretotean, CameliaDe Angelis, NicolaPinčák, RichardDe Aza, Piedad N.Pindelska, EdytaDe Azevedo, Afonso Rangel GarcezPineda, EloiDe Azevedo, Antonio Augusto C.Piñero Charlo, José CarlosDe Barros, AnerisePingitore, NicholasDe Biase, AlbertoPinho, Ana CatarinaDe Bilbao, EmmanuelPinilla, JavierDe Bona, FrancescoPinnaratip, RattapolDe Bonis, AngelaPinomaa, TatuDe Camargo, Felipe VannucchiPintaude, GiuseppeDe Castro, Carlos A. NietoPinto, GustavoDe Castro, Paulo M. S. T.Pinto, Joana VazDe Coninck, VincentPinzaru, IuliaDe Cózar, AbelPiotr, KruszewskiDe Feudis, MauroPiotrowski, TomaszDe Finis, RosaPiozzi, Antonellade Freitas Santos Júnior, AnibalPipintakos, GeorgiosDe Geest, BartPipiska, MartinDe Gonzalo, GonzaloPires, Ana FilipaDe Graeve, IrisPirola, SeleneDe Hoyos Martínez, Pedro LuisPirzada, TahiraDe La Fuente, Germán F.Pisano, GabrieleDe La Rosa, ÁngelPísařík, PetrDe La Rubia, Miguel AngelPisarski, WojciechDe La Torre, Angeles G.Pisciotta, AlessandraDe La Torre, UnaiPiscopo, GabrieleDe Lacalle, Luis N. LópezPisk, JanaDe León, ArxelPiskunov, Alexandrde Lourdes Pereira, MariaPislaru-Danescu, LucianDe Luca, OrestePismenskaya, NataliaDe Luca, StefaniaPisupati, AnuragDe Matos, PauloPiszczek, PiotrDe Matteis, ValeriaPitarch Roig, Angel MiguelDe Mey, GilbertPitarresi, GiuseppeDe Morais, Alfredo Manuel BalacóPiticescu, Radu RobertDe Morais, Paulo CesarPitsikalis, MarinosDe Mori, AriannaPitti, Rostand MoutouDe Moura, MarceloPitto-Barry, AnaïsDe Nalda, RebecaPivkin, PetrDe Oliveira Junior, MarcosPiwakowski, BogdanDe Oliveira, Ariel ColaçoPiz, MateuszDe Oliveira, Carolina CamargoPizoń, JanDe Oliveira, Helinando PequenoPizzo, BenedettoDe Oliveira, Tiago EspinosaPlachetka, UlrichDe Rességuier, ThibautPlachý, TomášDe Riccardis, M. FedericaPlaczkiewicz, JagodaDe Sousa, AnaPlakatouras, John C.De Sousa, José MoreiraPlank, HaraldDe Souza, Eder Carlos FerreiraPlantard, GaëlDe Souza, SivoneyPlassiard, Jean-PatrickDe Waele, WimPlatil, AntoninDe, ChandanPlatonenko, AleksandrsDe, ShuvodeepPleskunov, PavelDeac, CristianPleşu, NicoletaDeak, GyorgyPlevris, VagelisDeambrosis, Silvia MariaPlewa, JulianDebbarma, SolomonPlichta, AndrzejDebéda, HélènePłodzień, MarcinDeboucha, WalidPlokhikh, IgorDębska, BernardetaPlotniece, AivaDec, JanPlsek, JanDeck, PaulPlugin, Andrii A.Deepak, DoreswamyPluhařová, EvaDeev, RomanPluvinage, Guy A.Degmová, JarmilaPobelov, IlyaDehestani, MehdiPoccia, NicolaDehghan, AmirPodagatlapalli, G. KrishnaDehghanghadikolaei, AmirPoddelsky, AndreyDeifalla, Ahmed F.Podgurski, VitaliDeivanayagam, RamasubramonianPodkoscielna, BeataDeja, MariuszPodlasek, AnnaDekhtyar, JurijsPodrabinnik, Pavel A.Del Castillo-Santaella, TeresaPodraza, NikolasDel Hougne, PhilippPodulka, PrzemysławDel Prete, AntonioPogatscher, StefanDel Real-Romero, Juan CarlosPogrebnjak, AlexanderDel Río Merino, MercedesPoh, Yap SoonDel Sol Illana, IrenePohl, MichaelDel Toro, Eva M. GarcíaPokorný, JaroslavDelaunois, FabiennePokutnyǐ, Sergey I.Delcroix, DamienPolanski, MarekDeleporte, EmmanuellePolasek, OzrenDelgado, João M. P. Q.Poletaev, Gennady M.Delgado-Notario, Juan A.Policarpo, HugoDelgado-Sánchez, ClaraPolimeni, AntonioDelgado-Sanchez, Jose-MariaPolino, GiuseppinaDeligiannakis, YiannisPolisadova, ElenaDelimitis, AndreasPolitakos, NikolaosDeliverska, ElitsaPolitano, Grazia GiuseppinaDelivopoulos, EvangelosPoljak, MirkoDella Bella, ElenaPollicino, AntoninoDella Corte, GaetanoPolunin, Anton V.Delorme, NicolasPolyutov, SergeyDema, RomanPomastowski, Paweł P.Dematteis, ErikaPomoni, MariaDembinski, RomanPons, JaumeDement′ev, KonstantinPons, JosefinaDemeter, PeterPons, OriolDemetrescu, IoanaPont, UlrichDemirskyi, DmytroPonticelli, GennaroDemontis, ValeriaPonticelli, Gennaro SalvatoreDeng, FengxiaPop, NicolaeDeng, JiePop, ViorelDeng, PuPopa, FlorinDeniaud, AurélienPopescu, AurelianDenis, VivienPopescu, Ileana NicoletaDenisiewicz, ArkadiuszPopescu, RadianDeonikar, VirendrakumarPopescu, Sanda MihaelaDepan, DilipPopescu, VasilicaDepciuch, JoannaPopielarski, PawelDerazkola, Hamed AghajaniPopov, AnatoliDes Ligneris, ElisePopov, CyrilDesai, Prathamesh S.Popov, MikhailDesideri, DanielePopov, SergeiDespotovic, InesPopov, VladimirDesta, Kebede TayePopovic-Neuber, JelenaDestefanis, EnricoPorat, Ze′evDeterman, JohnPorojan, LilianaDettlaff, AnnaPorotnikova, NataliaDeuermeier, JonasPortan, DianaDeuszkiewicz, PiotrPortelli, MarcoDevaraj, RajamaniPortillo-Lara, RobertoDey, Debasish KumarPorto, Chiara LoDey, VikramPorubov, AlexeyDhakshinamoorthy, AmarajothiPosadas, PilarDhaliwal, Gurjot S.Poshtkohi, AlirezaDhamdhere, AjitPossan, EdnaDhanjai, DhanjaiPostawa, PrzemysławDhiman, InduPostek, EligiuszDhirajlal, Koyani RinaPot, GuillaumeDi Bartolomeo, AntonioPotapov, AndreiDi Benedetto, HervéPotapov, Vadim V.Di Caprio, FrancescoPotenza, FrancescoDi Cosola, MichelePotgieter, HermanDi Fiore, AdolfoPotocny, Andrea M.Di Franco, CinziaPotts, Sarah-JaneDi Gianfilippo, RiccardoPou, JuanDi Liberto, GiovanniPouchly, VaclavDi Maggio, RosaPoumellec, BertrandDi Michele, AlessandroPouraliakbar, HesamDi Nardo, FabioPouranian, M. RezaDi Nicola, CorradoPourbaghi, MiladDi Salle, AnnaPovolotskiy, Alexey V.Di Sarno, LuigiPow, Edmond Ho NangDi Spirito, FedericaPoyato, RosalíaDi Turo, FrancescaPozdniakov, AndreyDiaconu, Bogdan M.Prabhakar, Vattikuti S. V.Diamantini, Maria CristinaPrabowo, Aditya RioDiana, ManukovskayaPradhan, Dhiren K.Diana, VitoPradhan, ShantanuDiaz Blanco, Manuel JesúsPrado-Gonjal, JesúsDíaz De Tuesta, Jose LuisPrado-Velasco, ManuelDíaz García, PamelaPrakash, ChanderDíaz Lopez, OliverPrakash, NaveenDiaz, Camilo Arturo RodriguezPrakash, T.Díaz, EsperanzaPramanik, ArindamDíaz, IsabelPramanik, AtinDíaz, JosePramanik, BrahmanandaDíaz-García, DianaPramanik, TanmoyDìaz-Guerra, CarlosPramudita, Jonas A.Diddens, DiddoPranitha, SankarDiep, Hung T.Pranno, AndreaDieringa, HajoPrasad, AlishaDíez-Pascual, Ana MaríaPrasad, KarthikaDikova, TsankaPrasadh, SomasundaramDilip, J. J. S.Prashant, KumarDilonardo, ElenaPrashanth, Konda GokuldossDima, Stefan-OvidiuPraticò, Filippo GiammariaDimaggio, ElisabettaPravarthana, DhanapalDimitratos, NikolaosPredeanu, GeorgetaDimitrievska, MirjanaPredoi, Mihai ValentinDimitriou, VasilisPreez, S.P. DuDimitrova, RositzaPreinstorfer, PhilippDimos, KonstantinosPreisner, MichałDimter, SanjaPrelipceanu, MariusDinca, ValentinaPresa Soto, M. AlejandroDinelli, FrancoPresto, SabrinaDing, DongyanPreti, GiulioDing, JiaPribyl Ham, JuliaDing, JunfengPribyl, JanDing, Shinn-JyhPrida, Victor M.Ding, Wei DanPriego, Eva-MariaDinh, ToanPriel, EladDiniz, AnselmoPrieler, ReneDintcheva, Nadka TzankovaPrieto, Jose EmilioDinte, ElenaPrikhodko, IgorDINU, MihaelaPriola, EmanueleDiogo, PatriciaPrior, Timothy J.Dippong, ThomasPriyadarshi, RuchirDispinar, DeryaProcter, PhilipDistaso, MonicaProdan, GabrielDivić, VladimirProkhorov, Mikhail Evgen′evichDixon, David A.Promentilla, Michael AngeloDizaji, Hossein BeidaghyProriol Serre, IngridDjemia, PhilippeProrok, RyszardDjokić, JelenaProšek, TomášDjourelov, NikolayProskurnin, MikhailDługosz, OlgaProskurnina, Elena V.Dmitruk, AnnaProsolov, Konstantin A.Do Carmo, RicardoProstomolotov, Anatoly I.Do Nascimento, João Paulo CostaProsviryakov, AlexeyDo, Quoc CuongProto, AndreaDoan, DatProux, OlivierDoan, HoanProverbio, EdoardoDobkowska, AnnaPrpic, JelenaDobo-Nagy, CsabaPrugovečki, BiserkaDobrota, DanPruncu, CatalinDobrowolski, PiotrPrusov, EvgenyDobrzańska, JoannaPrysiazhnyi, VadymDobrzański, Lech B.Prytz, ØysteinDodun, OanaPrzekop, RobertDogossy, GáborPrzestacki, DamianDoh, JaehyeokPrzeździecka, EwaDoherty, BrendaPtáček, PetrDoi, KentaroPtiček Siročić, AnitaDojka, R.Pu, LumeiDojka, RafałPucci, AndreaDokšanović, TihomirPuertas, FranciscaDolata, Anna JaninaPugin, RaphaëlDolenec, RokPuglia, DeboraDolgoborodov, Aleksander YuPuigdollers, JoaquimĐolić, MajaPuiggalí, JordiDolinar, DragoPujadas Álvarez, PabloDolmatov, Dmitry O.Pukasiewicz, AndersonDolnik, BystrikPulatsu, BoraDomalik-Pyzik, PatrycjaPung, Aaron J.Dombek, GrzegorzPunset, MiquelDomek, GrzegorzPuosi, FrancescoDoménech-Carbó, AntonioPuri, SumantDomenici, ValentinaPushilina, NataliaDomenjoud, MathieuPushkar, SvetlanaDomingues, Eddy M.Pushkareva, IrinaDomínguez, CarlosPushpalal, DinilDominguez, Rocio B.Pušić, TanjaDominguez-Alfaro, AntonioPustovoytov, DenisDomínguez-Álvarez, EnriquePuszka, AndrzejDomitrović, JosipaPutignano, AngeloDomski, JacekPutra Jaya, RamadhansyahDoñate, Cristina MartínPutra, Teuku EdisahDonati, IvanPutta, AnjaneyuluDong, ChaofangPutz, Ana MariaDong, ChensongPuzniak, RomanDong, HaoPyatina, TatianaDong, HaowenPycia, KarolinaDong, HuaPyka, DariuszDong, KaiPylypchuk, IevgenDong, MengPyo, SukhoonDong, XiangyangPyrzanowski, PawełDonnermeyer, DavidQayyum, FaisalDonnini, JacopoQi, ChangDoquet, VeroniqueQi, DianpengDorado, RubenQi, HongDordevic, DaniQi, JunleiDorin, ThomasQi, LehuaDorner-Reisel, AnnettQian, DongjinDorofeev, Sergey G.Qiang, BinDorosz, DominikQiao, AikeDorozhkin, Sergey V.Qiao, ChunyuDorożyński, PrzemysławQin, GuoliangDorrah, AhmedQin, TianzhuDos Reis, Diogo DuarteQin, ZhaoyeDos Reis, Glaydson SimõesQing HongDos Reis, Paulo Nobre BalbisQing, JingjingDos Santos, GabrielQiu, GangDos Santos-García, Antonio JuanQiu, HaiDostál, IvoQiu, Hua-NingDoucet, MathieuQiu, JianbinDoustkhah Heragh, EsmaeilQiu, LinDoustkhah, EsmailQiu, MuqingDouvas, AntoniosQiu, QiwenDowon, BaeQiuji, YiDoynov, NikolayQu, ChuangDrábiková, JuliánaQu, FeijunDrabowicz, JózefQu, MuchaoDracopoulos, VassiliosQu, XiaozhongDragan, KrzysztofQuadroni, SilviaDragan, MirelaQuan, GaofengDraganová, KatarínaQuatravaux, ThibaultDraganovska, DagmarQuattrocchi, AntoninoDragatogiannis, Dimitrios A.Querales-Flores, JoseDragoman, DanielaQuijada, CésarDragostin, Oana MariaQuílez-Bermejo, JavierDrahos, BohuslavQuintero-Jaime, AndresDramicanin, MiroslavQuochi, FrancescoDrazic, GoranQuondam Antonio, SimoneDrevet, RichardQureshi, Mohamed RafikDrewniok, MichałQureshi, TanvirDrexler, AndreasQuzai, MoinDreyer, Chris H.R. Labrosse, MichelDrochioiu, GabiRabajczyk, AnnaDrozdov, Aleksey D.Rabczuk, TimonDrozdov, Andrey S.Rabiee, NavidDruesne, FredericRached, HabibDrummond, Colin K.Racko, DusanDrygaś, PiotrRacoviceanu (Babuta), RoxanaDryzek, JerzyRacovita, StefaniaDrzazga, MichałRacuciu, MihaelaDţeanu, NarcisRacz, Sever-GabrielDu Roscoat, Sabine Rolland OllandRaczkiewicz, WiolettaDu, Cheng-FengRadacsi, NorbertDu, HongjianRadamson, Henry H.Du, JingshanRadan, MilaDu, LipingRadchenko, Eugene V.Duan, YongxinRadek, NorbertDuan, YuechenRadenkovs, VitalijsDuarte Marafona, JoséRadev, DimitarDuarte, António P.C.Radi, EnricoDuarte, NoéliaRaditoiu, AlinaDub, Alexei V.Raditoiu, ValentinDubashynskaya, Natallia V.Radtke, AleksandraDubey, Bipro N.Radu, DorinDubinskiy, SergeyRadu, NicoletaDubnika, AritaRadwański, KrzysztofDubois, AndréRadziuk, DaryaDuchosal, ArnaudRaeisHosseini, NiloufarDuchoslav, JiriRaffelt, KlausDucobu, FrançoisRagab, AdhamDudás, ZoltánRaganati, FedericaDudek, AgataRagauskas, ArthurDudek, Krzysztof K.Raghavan, SreevatsanDudek, MagdalenaRaghunandan, Mavinakere EshwaraiahDudina, DinaRagula, Udaya Bhaskar ReddyDudina, Dina V.Ragusa, AndreaDudova, NadezhdaRahier, HubertDuenas, José AntonioRahimi, EhsanDukarska, DorotaRahimi, ParvanehDulal, RajendraRahimi, SohrabDulčić, NikšaRahimian Koloor, Seyed SaeidDulińska, Joanna MariaRahman, M. AzizurDuliu, OctavianRahman, Mohammad MizanurDulska, AgnieszkaRahman, MuhibDuma, Virgil-FlorinRahman, Muhommad AzizurDumitrascu, GheorgheRahmani Ahranjani, RaminDumitriu, CristinaRahmani, FarzinDumitru, MihaiRahmani, RaminDumur, FrédéricRahmoune, MiloudDunaj, PawelRahul, Attupurathu VijayanDunlop, TomRaiszadeh, RaminDuöng, Ngoc My HanhRaj, Pulugurtha MarkondeyaDupláková, DarinaRaja, Iruthayapandi SelestinDura, Oscar J.Rajabi, ArminDurante, MassimoRajabi-Abhari, ArazDurán-Valle, Carlos J.Rajak, Dipen KumarDuspara, MiroslavRajan, KalavathyDutour Sikirić, MajaRajesh, John AnthuvanDutournié, PatrickRaji, AtchudanDutta, AnushreeRajska, MariaDutta, BiswanathRajtukova, ViktoriaDutta, GaurabRaju, KatiDutta, Ravi ChandraRakass, SouadDuvigneau, FabianRakhadilov, BauyrzhanDvorský, DrahomírRakhshan, VahidDvorský, TomášRakitin, Oleg A.Dwivedi, SumantRakoczy, RafalDworecka-Wójcik, JulitaRamachandran, Muggundha RaoovDyachkova, LarisaRamachandran, RoshiniDyl, TomaszRamaglia, GiancarloDymáček, PetrRamakrishnan, SayanthanDymkowska, DorotaRamalingam, SundarDymshits, OlgaRamallo, Manuel V.Dzedzickis, AndriusRamamoorthy, Sunil Kumar LindströmDziak, RosemaryRamani, AlwarDzida, MarzenaRamani, SujathaDzięcioł, JustynaRamazanov, ShikhgasanDziedzic, AndrzejRamesh, KalyanDzierwa, AndrzejRamesh, SivalingamDzimitrowicz, AnnaRamirez, ArielDziubak, TadeuszRamirez, CristinaDziubek, KamilRamirez, Sagrario MartínezDzwonkowski, ArielRamirez-Castellanos, JulioE. Golub, IgorRamos, NathaliaEarmme, TaeshikRamos-Garcia, PabloEberlein, RobertRamos-Ortiz, GabrielEbrahimi, AminRana, Abu Ul Hassan SarwarEbrahimi-Mamaghani, AliRanaweera, Aruna KumaraEbrahimnezhad-Khaljiri, HosseinRancan, MarzioEbralidze, Iraklii I.Randall, DyllonEchempati, RaghuRanganathan, NarayanaswamiEchevarria-Bonet, CristinaRanganathan, PalrajEcker, MelanieRangel, AndréEckschlager, TomasRanjan, AlokEconomou, DemetreRanjbar, NavidEdacherian, AbhilashRao, Posinasetti NageswaraEdagawa, KeiichiRapiejko, CezaryEdelhauser, EduardRapone, BiagioEdelmann, JanRaposeiras, Aitor C.Effenberg, FlorianRaposeiro Da Silva, PedroEfremenko, VasilyRaposo, MariaEfremov, AlexanderRashid, Rizwan RahmanEfthimiopoulos, IliasRasing, TheoEfthymiadis, PanosRasoga, OanaEftimie Totu, EugeniaRastin, HadiEftimie, MihaiRatajski, JerzyEgan, Paul FRath, PunyaslokEgedy, AttilaRathaiah, MamillaEgiza, MohamedRathinam, KarthikEglitis, RobertsRathod, Vivek T.Egorin, AndreiRatvik, ArneEguía-Barrio, AitorRauh, AndreasEhrmann, AndreaRault, FrancoisEibl, SebastianRaut, PrasadEid, RamiRavelli, DavideEinkauf, Jeffrey D.Ravichandran, DharneedarEiras Fernández, JesúsRavichandran, KollarigowdaEisenlohr, PhilipRavindra, Nuggehalli M.Eiser, ErikaRavindran, RaviEkanem, EkanemRavula, ThirupathiEl Badawy, SherifRay, ApurbaEl Garah, MohamedRay, SujayEl Khaled, DaliaRaynova, StellaEl Khalloufi, FatimaRazavi, RaziehEl Kharbachi, AbdelRazmi, JafarEl Khatib, MohammadRazmpoosh, M. HadiEl Oualkadi, AhmedRazumovskiy, VsevolodEl Seoud, Omar A.Razzak, TowhidurElahi, HassanRebecca, PiovesanElaissari, AbdelhamidRebollar, EstherElakneswaran, YogarajahReboul, NadègeElambasseril, JoeRećko, KatarzynaElbendary, AshrafReczek, Joseph J.Elchaninov, AndreyReczyńska, KatarzynaEl-Din Hassouna, Mohamed SalahReda, RodolfoEleftheriadis, GeorgiosReddicherla, UmapathiElena, HusanuReddy, Hariprasada R. KannaEleonora, RussoReddy, M. Siva PratapElert, KerstinReddy, Y. Veera ManoharaElgarahy, Ahmed M.Redfern, JamesElhachmia, Ech-chihbiRedhammer, Günther JosefElhadidi, BasmanRegev, MichaelEl-Hassan, HilalRehman, SuriyaElias, SaidReincke, KatrinEligiusz, PostekReis, PauloEliseev, AlexanderReis, Ruham PabloElkady, OmaymaReisinger, AndreasElkaseer, AhmedReis-Machado, AnaEl-Kelany, Khaled E.Reiterman, PavelEllahi, RahmatRekas, MieczyslawEllendt, NilsRelucenti, MichelaEllermann, KatrinRemes, ZdenekEl-Maaddawy, TamerRempel, SergejElmi-Terander, AdrianRen, DonglouElmouwahidi, AbdelhakimRen, MinghuaEl-Newehy, MohamedRen, NaElreedy, AhmedRen, QiangElsafi, MohamedRen, QinlongEl-Sayed, Ahmed Ma A.Ren, ShisongElsheikh, AmmarRen, YongshengElsts, EdgarsRenaudin, GuillaumeEltaggaz, AbdelkremRendenbach, CarstenEltit, FelipeRengo, SandroEl-Wafa, Mahmoud AboRenoud, RaphaëlElwakeel, KhalidRescalvo, FranciscoEl-Zohairy, AymanReshetov, VladimirEmanuel, IonescuRešković, StojaEmelyanenko, KirillRestivo, JoãoEmery, AshleyReukov, VladimirEmery, JohnReuland, YvesEmilio, Martín-GutiérrezReviznikov, Dmitry L.Encina-Zelada, Christian R.Revuelta Crespo, DavidEnesca, AlexandruReyes, CarolinaEngel, NadjaReyes, EncarnaciónEnikeev, NarimanReyes, PedroEnnen, IngaReyes-Gasga, JoséEnrione, JavierReynier, NicolasEranga Wijesinghe, RuchireReza, SumonErcegović Ražić, SanjaRezaei, PejmanErck, RobertRezaei, ShahedErdakov, Ivan NikolaevichReza-E-Rabby, MdEremenko, IgorRezania, ShahabaldinEremeyev, VictorRezayat, HassanEremin, ArtemRezazadeh, HadiErnesto-Casanova, OscarRezek, JiříErofeev, Vladimir I.Rhi, Seok-HoEROVIC, Boban M.Riba, Jordi-RogerErps, JürgenRibakov, YuriErrandonea, DanielRibeiro, Carlos A. SilvaEscamilla, AlfonsoRibeiro, JoãoEscobedo, SalvadorRibeiro, José BarandaEscolano, FélixRibeiro, Paulo A.Escorihuela, JorgeRibeiro-Claro, PauloEscudero, AlbertoRibić, RosanaEsen, CemalRicca, ChiaraEsfahani, Mohammad NasrRiccardi, ChiaraEslami, BabakRiccardi, ClaudiaEslami, HosseinRiccardo, ScalengheEsmaeeli, RojaRicci, Pier CarloEsmizadeh, ElnazRiccio, AnielloEspinach, Francesc XavierRicco, RaffaeleEsposito, AntonellaRichard, HrčkaEsposito, LucaRichert, MariaEsquivel, Rodolfo O.Richter, ClaudiaEstevez, RafaelRichter, KatharinaEstokova, AdrianaRichtera, LukasEstrga-arriaga, EdsonRicotta, VitoEtacheri, VinodkumarRiech, InesEugene, FeldshteinRiedl, ThomasEvangelista, AnaRiegel, HaraldEvangelisti, ClaudioRiekehr, StefanEvans, BenjaminRiess, GisbertEvin, EmilRifai, AaqilEvlashin, Stanislav A.Rigaud, MichelEvtugyn, GennadyRigon, DanieleEwa, SkwarekRiha, Shannon C.Exarchos, Dimitrios A.Riis Porsborg, SimoneExarhopoulos, StylianosRijckaert, HannesExner, Ginka K.Rikmann, ErgoEze, Fredrick NwudeRinaldi, DanieleEze, VincentRíos, José D.Ezenwa, Innocent C.Ríos-Carrasco, BlancaEzugwu, SabastineRipamonti, DarioFabbri, FilippoRipani, MarianelaFabbrocino, FrancescoRiposan, IulianFabbrocino, GiovanniRischka, KlausFaber, KatherineRisitano, GiacomoFabianowski, WojciechRispoli, ConcettaFabijański, MariuszRitacco, TizianaFabio, MarmottiniRiu, Doh-HyungFabisiak, KazimierzRius-Pérez, SergioFabris, NicolaRiveiro Rodriguez, AntonioFacchetti, GiorgioRivera-Diaz-del-Castillo, Pedro E. J.Fadonougbo, Julien O.Rivera-Gómez, CarlosFæster, SørenRivero, Pedro J.Fahem, AliRiviera, Pier PaoloFahmi, KarimRivière-Lorphèvre, EdouardFaia, PedroRiyanto, EdyFakher, SherifRizzarelli, PaolaFakhri, MansourRizzi, Gian AndreaFakih, Mohammad AliRizzolio, FlavioFakin, DarinkaRoatǎ, Ionuţ ClaudiuFakouri Hasanabadi, MasoodRobache, FredericFal, JacekRoberto, RobertiFalabella, EduardoRobinson, SeriFalacho, RuiRocca, RobertoFalcao, CarlosRoccaforte, FabrizioFalcão, Daniela S.Roccuzzo, AndreaFalchetto, Augusto CannoneRocha, DjenisaFaleschini, FloraRocha, FernandoFalini, AntonellaRocha, Iuri B. C. M.Falkowski, PawełRochani, AnkitFalliano, DevidRocha-Rangel, EnriqueFamengo, AlessiaRochfort, KeithFan, ChenleiRodak, KingaFan, JiajieRodchanarowan, AphichartFan, XiaoguangRodic, DraganFan, YiqiangRodič, PeterFan, ZhengjieRodkevich, NikolayFanelli, PierluigiRodrigo-Ilarri, JavierFang, ChangmingRodrigue, DenisFang, Chih YuanRodrigues Menezes, RomualdoFang, GuozhaoRodrigues, Alisson MendesFang, HaixingRodrigues, Patrícia FreitasFang, KegongRodrigues, Pedro M.S.M.Fanijo, Ebenezer O.Rodrigues, Samuel F.Fanizzi, Francesco PaoloRodríguez González, ÁlvaroFanteria, DanieleRodríguez Millán, MarcosFantilli, Alessandro P.Rodríguez Pasandín, Ana MaríaFarabi, EhsanRodríguez Saiz, ÁngelFarago, PaulRodriguez, Aurelio HierroFarahani, Behzad V.Rodriguez, Diana M.Faraldos, MarisolRodriguez, DulceFaran, EilonRodríguez, ErichFaraudo, JordiRodríguez, Juan FranciscoFarhan, RidaRodriguez, MauricioFaried, Ahmed SeragRodriguez, NoelFarina, IleniaRodriguez, PilarFarinha, Catarina Correia De Araújo BrazãoRodríguez, RafaelFarjas, JordiRodríguez-Alabanda, ÓscarFaro, Alessandro LoRodríguez-Alloza, Ana MaríaFarooq, ImranRodríguez-Lozano, Francisco JavierFarooq, Muhammad UmarRodriguez-Martin, ManuelFarooq, SherRodríguez-Tinoco, CristianFarrokh Ghatte, HamidRogachev, ArtemFarronato, GiampietroRogachev, S. O.Farshidi, Mohammad HassanRogala, MichałFaruqi, Mohammed A.Rogale, DubravkoFarzampour, AlirezaRogers, SimonFasano, FabrizioRoggendorf, HansFasolino, InesRogkala, AikateriniFatemeh, JahanmardRogovoy, Anatoly A.Fathalla, EissaRogozhnikov, DenisFathollahzadeh, HomayounRogoziński, TomaszFaur, CatherineRogulj, KatarinaFavier, IsabelleRogulska, MagdalenaFavieres, CristinaRoh, ChanghyunFavre-Réguillon, AlainRoh, Jea-SeungFayemi, Omolola E.Roh, SanghoFederico, ConcettaRoh, YoungsukFedin, MatveyRoh, YulFediuk, RomanRokaya, DineshFedorkova, AndreaRokicka-Konieczna, PaulinaFedorov, SergeyRokos, OndrejFedorov, VladimirRolfe, PeterFedorowicz, JoannaRom, MonikaFedorowicz, Joanna DagmaraRoman, AlexandraFedotov, SergeyRomanczuk, ElizaFei, TongRoman-Flores, ArmandoFeier, AnamariaRomann, TavoFeito, NorbertoRomano, Jean-MichelFeizpour, DarjaRomano, StefaniaFeldshtein, Eugene ÉRomanovski, ValentinFelgueiras, HelenaRománszki, LorándFelipe González, CataldoRomao, CarlFeller, Jean-FrancoisRomero, AlbertoFenerci, AkselRomero, Carlos SantiusteFeng, FengRomero, EsperanzaFeng, HongboRomero, Francisco J.Feng, HuanhuanRomero, PedroFeng, QihongRomero-Piedrahita, Carlos AlbertoFeng, RuiRommozzi, ElenaFeng, ShaochuanRonavari, AndreaFeng, Sheng-WeiRonca, AlfredoFeng, ShuanglongRončević, Ivana ŠkugorFeng, ZhiyuanRong, YouminFerdous, WahidRonkay, FerencFerdov, StanislavRoohi, EhsanFérec, JulienRorrer, GregFerenc, TomaszRosa, PedroFernandes, FábioRosalie, JulianFernandes, GustavoRoscini, FrancescaFernandes, Jucielle VerasRoscow, J.Fernandes, Maria GoretiRosenkranz, AndreasFernandes, Maria HelenaRosenzweig, DerekFernandes, Ricardo M. F.Rosero-Navarro, CarolinaFernandez Fairén, MarianoRoshanghias, AliFernández González, DanielRoshani, SobhanFernández Martínez, FranciscoRoshchina, SvetlanaFernández, AdolfoRoskosz, MaciejFernandez, Angel G.Rossella, FrancescoFernandez, Antonio JesusRossetto, MassimoFernandez, JavierRossi, RiccardoFernandez, JoseRosso, CarloFernandez, Jose MaríaRosso, StefanoFernández, Juan PedroRostami, JavadFernández, PalomaRostas, Arpad MihaiFernández-Calviño, DavidRostov, Vladislav V.Fernandez-Campos, FranciscoRoszek, KatarzynaFernández-Catalá, JavierRotaru, HoratiuFernández-González, DanielRovere, CarlosFernando, AnuraRovere, MassimoFernando, Palamandadige K. S. C.Rovisco, AnaFerone, ClaudioRovnaník, PavelFerrández, DanielRowe, BrianFerrara, GiuseppeRowell, Roger M.Ferraris, SaraRoy Choudhury, ShreyaFerraro, AlbertoRoy, ArunabhaFerraro, DarioRoy, IndranilFerreira Santos, SydneyRoy, JugantaFerreira, Manuel MarquesRoy, KrishanuFerreira, MarioRoy, PraneshFerreira, Nuno MRoy, SougataFerreira, Nuno R.Roy, SwarupFerrer, Ranier Enrique SepúlvedaRoychand, RajeevFerrero Martín, Francisco J.Rožánek, MartinFerretti, ElenaRóżańska, SylwiaFerro, PaoloRozbroj, JiříFerrotti, GildaRozumek, DariuszFeuchter, MichaelRozwadowski, TomaszFey, TobiasRuan, JujunFicek, MateuszRuangrassamee, AnatFidan, IsmailRubčić, MirtaFierascu, IrinaRuben, Rui B.Fierro, VanessaRubino, FeliceFigueira, FlavioRubinowska, KatarzynaFigueira, Rita BacelarRubio Alonso, FaustoFigueiredo, CarlosRubio Alonso, JuanFigueras-Alvarez, OscarRubio, Ramón GonzálezFigueroa, RaulRučigaj, AlešFikar, JanRucińska, TeresaFikus, BartoszRudakova, Natalya V.Fíla, TomášRudnicki, TomaszFilgueira, CarlyRudolf, RebekaFilip, PeterRueggeberg, FrederickFilip, PetrRuffini, AndreaFilipecki, JacekRuffino, BarbaraFilipescu, MihaelaRuggiero, AndrewFilippatos, AngelosRuiz, DavidFilippov, Andrey V.Ruiz, Jesús FernándezFiliz, SeckinRuiz, OscarFilonova, ElenaRuiz-Zepeda, FranciscoFina, IgnasiRumyantseva, MarinaFinkel, PeterRupal, Baltej SinghFinkelstein, ArkadyRuscica, GiuseppeFinocchio, ElisabettaRusen, EdinaFinšgar, MatjažRusnáková, SoňaFiol Oliván, FranciscoRusso Spena, PasqualeFiołek, AleksandraRusso, AngelaFiore, EnricoRusso, FrancescaFiore, SilviaRusso, PietroFiorenza, PatrickRusso, VincenzoFiorenza, RobertoRusu, Bogdan-GeorgeFiori, FabrizioRusu, DanielaFiorio, RudineiRüter, Christian E.Fischer, Franz DieterRutkowska, GabrielaFischer, Hartmut R.Rutkowska, KatarzynaFischer, SarahRutkowska-Zbik, DorotaFischer, SzabolcsRutkowski, PawełFischerauer, GerhardRuwoldt, JostFischetti, Massimo VRuys, AndrewFisher, John G.Ruysschaert, Jean MarieFitzek, HaraldRužiak, IvanFleck, MichaelRuzic, JovanaFlegner, PatrikRůžička, AlešFleischer, JürgenRyan, ShannonFlieger, JolantaRybak, AlexanderFlores Cuautle, Jose De Jesus AgustinRybak, JarosławFlores, EduardoRydosz, ArturFlores-Alés, VicenteRydz, DariuszFlorescu, MonicaRydz, JoannaFogacci, FedericaRydzkowski, TomaszFogarasi, SzabolcsRyjacek, PavelFokter, Samo K.Rykowska, IwonaFoley, MatthewRylski, AdamFolino, PaulaRyms, MichalFolpini, GiuliaRyńska, ElżbietaFonseca, AnaRyou, HeonjuneFonseca, Elza Maria MoraisRyparova, PavlaFontanari, VigilioRys, DawidFontanesi, ClaudioRyszkowska, JoannaFoot, PeterRytlewski, PiotrForaboschi, PaoloRytter, ErlingForay, GenevieveRyu, Gyeong HeeForcellini, DavideRyu, HwaFord, NicoleRyu, Jae-JunFormisano, AntonioRyu, JehwangFornaro, AdalgizaRyzhikov, AndreyFornell, AnnaRyzhkov, SergeiForner, LeopoldoSa, BaishengForouzan, FarnooshSaadati, MohammadFořt, JanSabarirajan, Dinesh C.Fortuna, SergeiSabarou, HoomanFoschi, FedericoSabău, EmiliaFoti, ClaudiaSabbagh, FarzanehFoti, DoraSabik, AgnieszkaFoucher, DanielSaboori, AbdollahFourmousis, IoannisSachenkov, OskarFox, NiamhSackmann, JohannesFracasso, GiulioSadayappan, KumarFracchia, ElisaSadeghi Tabar, RohamFrączek, TadeuszSadeghianmaryan, AliFraddosio, AguinaldoSadeghifar, HasanFraga, DiegoSadovnikov, AlexandrFraga-López, FranciscoSadowska, KamilaFragassa, CristianoSadowska-Buraczewska, BarbaraFrança, RodrigoSadowski, TomaszFrancesca, RosciniSadraei, RaziehFranceschini, GiordanoSaeed, AnwarFranchini, FaustoSaeed, Muhammad AhsanFranco, Camilo AndresSaeedikhani, MohsenFrancolini, IolandaSaenboonruang, KiadtisakFranconetti, AntonioSaenger, Erik H.Frank, Eduardo N.Sáenz De Argandoña, EnekoFrank, JohannesSafaei, Mohammad RezaFrank, LuizaSaffell, JohnFrankovska, JanaSafonov, Alexander AleksandrovichFrankovsky, PeterSafyari, MahdiehFranz, GeraldSagadevan, SureshFranz, GerhardSah, InjinFranzoni, FelipeSaha, BiswajitFrassetto, FabioSaha, DulalFráter, MárkSaha, NabanitaFratini, FabioSaha, RatnajitFrattini, DomenicoSaha, RenataFrazier, MichaelSahoo, Chinmaya KumarFreddi, FrancescoSahoo, Jugal KishoreFrédéric, MuttinSahoo, PathikFredi, GiuliaSahul, MiroslavFreire, Ana CristinaSai, HiroakiFreitas Rodrigues, PatríciaSaid Schicchi, DiegoFreitas, ManuelSaidani, MessaoudFresch, BarbaraSaielli, GiacomoFresco-Cala, Beatriz MaríaSaifutdinov, AlmazFréty, RogerSaifutdinov, Bulat R.Friák, MartinSaikko, VesaFrick, JurgenSaikova, SvetlanaFridric, VincentSail, LatefaFridrihsone-Girone, AndaSaimoto, AkihideFried, MiklosSaini, NaurangFriederich, FabianSaint-Fort, RogerFriedmann, AntonSainz-Aja, Jose A.Friesenbichler, WalterSaito, HiromuFriso, DarioSaito, KazuyaFroelich, WojciechSaito, ShimpeiFröhlich, PeterSaitoh, HiroyukiFrondelius, TeroSaitoh, Ken-ichiFryczkowska, BeataSaitoh, MasatoFrydrych, KarolSajewicz, EugeniuszFryska, SebastianSaji, ViswanathanFu, LeSajjad, ArbazFu, PingfengSajkiewicz, PawełFu, ShuaiSakač, NikolaFu, SuleiSakai, JoeFu, Yin-ChihSakai, KatsuhikoFu, Zhi QiangSakai, MasamichiFu, ZhuojiaSakai, MotomuFuchs-Godec, ReginaSakamoto, HiroakiFucikova, AnnaSakanakura, HirofumiFuente, ElenaSakellis, EliasFugetsu, BunshiSakhabutdinov, Airat Zh.Fujihara, TakashiSakharova, NataliyaFujii, KenjiSakon, TakuoFujima, NobuhisaSakowicz, MaciejFujioka-Kobayashi, MasakoSaksl, KarelFujisawa, KiyoshiSakthivel, KogularasuFujiwara, KozoSakurai, ShinichiFunahashi, MasahiroSala, RobertoFürbeth, WolframSałaciński, TedeuszFurgal, JosephSalami, BabatundeFurko, MonikaSalaoru, IuliaFurlan, Kaline P.Salas, CarlosFurlani, FrancoSałasińska, KamilaFurlani, MaurizioSalauddin, MdFurtado, AndréSalaün, MathieuFurtos, GabrielSalcedo, RobertoFuruse, Adilson YoshioSaleemi, SidraFuruya, MasahiroSaleh, BassiounyFutakawa, MasatoshiSaleh, MonaFutalan, CybelleSalehizadeh, AliFutáš, PeterSalerno-Kochan, RenataFydrych, DariuszSalgarello, StefanoGabbianelli, GiammariaSalim, HaniGabrić, DraganaSalim, Muath BaniGabriele, MagnaSalimian, AliGabrielli, ValeriaSalimi-Kenaria, HamedGackowski, MariuszSalimon, AlexeyGadalińska, ElżbietaSalina Borello, EloisaGadallah, RamySalinas-Torres, DavidGaddam, AnveshSalit, Mohd SapuanGadisa, Abay DinkuSalje, EkhardGadomska-Gajadhur, AgnieszkaŠalkus, TomasGadomski, AdamSallam, Hossam El Din M.Gafurov, MaratSalman, MootazGagarin, AlexanderSalmas, ConstantinosGagieva, Svetlana C.Salopek Čubrić, IvanaGagliardi, FrancescoSalunkhe, RahulGahruie, Hadi HashemiSalur, EminGaida, Nico AlexanderSalvado, VictoriaGaihre, BipinSalvati, EnricoGain, Asit KumarSalvestrini, StefanoGajdoš Kljusurić, JasenkaSalvetr, PavelGajewski, MarcinSalzenstein, FabienGajewski, TomaszSamadi-Dooki, ArefGajtanska, MiladaSamal, SangramGalanakis, IosifSamal, SnehaGalao, OscarSamanta, AvikGalatanu, AndreiSamanta, ShibnathGalazka, MiroslawSamaouali, AbderrahimGalgalikar, RohanSamaras, IoannisGalinetto, PietroSambataro, SergioGalkiewicz, JaroslawSambath, KarthikGall Troselj, KoraljkaSamborski, SylwesterGallardo, Vanessa ParedesSambucci, MatteoGallegos, AlbertoŠamec, DunjaGalli, CarloSamodurova, MarinaGalli, MassimoSamoila, CornelGallo Cordova, AlvaroSamoilova, OlgaGallo, SalvatoreSamołyk, GrzegorzGallo, SantiagoSamoylov, Alexander M.Gallo, SimoneSampath, DhinakaranGalvão, IvanSamperi, FilippoGamage, J.C.P.H.Samsudin, Mohamad Fakhrul RidhwanGambardella, AlessandroSamudrala, GopiGambelli, AlbertoSamuel, Fawzy-HosnyGamboa, Carolina BermudoSamuha, ShmuelGamboa, Mario MartínSanbhal, Noor A.Gamec, JánSánchez Egea, Antonio J.Gamelas, JoséSánchez García-Vacas, DanielGamez Montero, Pedro JavierSanchez, Jon MikelGamez-Perez, JoseSanchez, Jose M.Gammer, ChristophSánchez, María TeresaGan, DanSánchez, Rafael S.Gancarz, TomaszSanchez-de-la-Muela, Alejandro-MiguelGanczarski, Artur WładysławSánchez-Martín, María JesúsGandelli, EmanueleSánchez-Molina, D.Gandini, PaolaSanchez-Perez, ArturoGandji, Navid P.Sanchez-Ruiz, AntonioGaneev, ArturSánchez-Vergara, María ElenaGanesamoorthy, RamasamySanctis, ShawnGanesh, Chandra DhalSanderson, JosephGanesh, R. SankarSandnes, EspenGangadharan, RajanSandu, Andrei VictorGangareddy, JagannathSandu, ViorelGanguly, SayanSăndulescu, MihaiGangwar, KapilSanina, NataliyaGanio, MonicaSanli, AbdulkadirGänser, Hans-PeterSan-Martin, DavidGao, BoSanna, DanieleGao, ChaoSans, Juan AngelGao, HuichangSantamaria, MonicaGao, JieSantamaría-Pérez, DavidGao, JinSantana, Juan A.Gao, JingjingSantangelo, SaveriaGao, LinyueSanter, SvetlanaGao, LizengSanthosha, Aggunda L.Gao, XiaohuiSantiago Junior, Joel FerreiraGao, YunjiŠantić, AnaGarabedian, NikolaySantini, SilviaGarašić, IvicaSantoni, Marie-PierreGaray Reyes, Carlos G.Santos, Emanuel, Jr.Garbiec, DariuszSantos, Francisco Eroni Paz DosGarbowski, TomaszSantos, JeniferGarcez, Estela OliariSantos, João M.García Calvo, José LuisSantos, PauloGarcía Moreno, FranciscoSantos-Coquillat, Ana MariaGarcía Rodríguez, JuanSantovito, GianfrancoGarcía Salvador, RosaSantulli, CarloGarcía Suárez, Víctor ManuelSantus, CiroGarcia, Coralia V.Sanyal, UdishnuGarcía, DanielSaoud, KhaledGarcia-Baños, BeatrizŠapalas, AntanasGarcía-Fogeda, PabloSapienza, VincenzoGarcía-Frutos, Eva MaríaSaputera, Wibawa HendraGarcia-Gallego, SandraSarafidis, Charalampos S.García-Gasca, Silvia AlejandraSaraiva, Gilberto DantasGarcía-Giménez, RosarioSaraswat, VivekGarcía-León, Ricardo AndrésSaratale, GaneshGarcía-López, ErikaSaratale, Rijuta GaneshGarcía-Martínez, Jesús-MaríaSaravanakumar, KandasamyGarcía-Martinez, Joaquín C.Saravanakumar, KarunamoorthyGarcia-Moreno, FranciscaSaravanan, PrabakaranGarcía-Navarro, Francisco JesúsSaravanan, S.García-Peñas, AlbertoSarbu, AndreiGarcia-Salaberri, Pablo A.Sareh, PooyaGarcía-Salinas, María JoséSarikaya, MuratGarcía-Sanz, VerónicaSarikov, AndreyGarcia-Taengua, EmilioSarkar, BiplabGarcía-Valenzuela, AurelioSarkar, Himadri SekharGardelis, SpirosSarkar, SayanGareev, KamilSarker, DyutiGargiulo, ValentinaSarlin, EssiGarino, NadiaSarosi, CodrutaGaripcan, BoraSartorio, CamilloGarmendia, IñakiSaruhan, BilgeGaroli, DenisSarvestani, Hamidreza YazdaniGaroush, SufyanSarychev, Vladimir D.Garriga, RosaSas, WojciechGarroni, SebastianoSasai, YasushiGarstka, TomaszSasaki, SonoGartia, Manas RanjanSasamoto, HiroshiGarus, SebastianSassani, AlirezaGarzon Hernandez, SaraSassu, MauroGarzon, EduardoSastoque Pinilla, LeonardoGashti, Mazeyar ParvinzadehSastry, Shankar M. L.Gasilova, Ekaterina R.Satam, ChinmayGáspár, Gáspár MarcellSatapathy, SitakantaGáspár, LászlóSathish, CIGatin, AndreySato, YukioGatto, AndreaSatyanaga, AlfrendoGauden, PiotrSaumik Panja, SaumikGauden, Piotr A.Sauter, EricGaudry, ÉmilieSava, Bogdan AlexandruGautam, SiddharthSava, IonGauterin, FrankSavage, Daniel J.Gauthier, RémySavaidis, GeorgiosGavalas, EvangelosSaveleva, MariiaGavali, HindaviSavelli, GuillaumeGavras, SarkisSavin, AlexanderGavrila, HoriaSavina, IrinaGawdzik, BarbaraSavio, GianpaoloGaweł, TomaszSavoie, Jean-MichelGawenda, TomaszSavov, ViktorGawlik, JozefSavovic, SvetislavGawri, RahulSavrai, RomanGaxiola-Camacho, José RamónSawik, BartoszGaya, AbinashSawka, AgataGazda, PiotrSaxen, HenrikGazzano, MassimoSaxena, KuldeepGbaguidi, AudreySaxena, PrateekGe, DongdongSayaka, SuzukiGe, Jun CongSayan, Deb DuttaGe, YunfengSayardoust, SharielGębara, PiotrSayed, Ibrahim M.Gebauer, JanSayyadi, NimaGebhardt, AndreasSayyed, Mohammed IbrahimGebremariam, Kidane FantaSbiaai, KhalidGece, GokhanScacco, SalvatoreGedvilas, MindaugasScaffaro, RobertoGeer, Ana M.Scala, FabrizioGehre, PatrickScalet, GiuliaGeisler, RamsiaScamporrino, Andrea AntoninoGelu Ovidiu, TirianScandurra, AntoninoGencel, OsmanScaringi, GianvitoGenerowicz, AgnieszkaScarso, AlessandroGeng, DechaoScatena, RebeccaGennadevich, Galkin NikolayScattina, AlessandroGennari, ClaudioScendo, MieczyslawGennaro, RenatoSchabowicz, KrzysztofGennaro, SylvainSchäfer, EdgarGenova, VirgilioSchäublin, RobinGenovese, AndreaScheau, CristianGentile, DomenicoScheeline, AlexanderGentile, GennaroScheer, Hella-ChristinGeorgantzinos, Stelios K.Scheerer, HerbertGeorgieva, Milena G.Scheerer, SilkeGeorgii, RobertScheffel, Débora Lopes SallesGeorgiou, EmmanuelScheidl, RudolfGeorgopanos, ProkopiosSchell, JulianaGeorgouli, KonstantinaSchenk, ThomasGer, Ming DerSchepler, KennethGerbeth, GunterSchiavi, JessicaGereke, ThomasSchiavi, Pier GiorgioGericke, OliverSchicchi, Diego SaidGerislioglu, BurakSchierz, OliverGesuele, FeliceSchillinger, BurkhardGhabchi, RouzbehSchilm, JochenGhaffar, SeyedSchimmenti, RobertoGhaffarian, Mohammad SaeidSchindler, IvoGhahari, Seyed AliSchintke, SilviaGhalehnovi, M.Schio, LucaGhalkhani, MasoumehSchiraldi, David A.Ghanbari, AbbasSchleifenbaum, Johannes HenrichGhanbari, SiavashSchlick, GeorgGhani, ArfanSchlosser, ViktorGharaei-Moghaddam, NimaSchmelzer, Jürn W.P.Gharehdash, SabaSchmickler, WolfgangGhasemlou, MehranSchmidt, BernhardGhayoor, MiladSchmidt, FranziskaGhazijahani, Tohid GhanbariSchmidt, MichaelGheni, Ahmed A.Schmidt, OlafGherlone, Enrico FeliceSchmidt-Ott, AndreasGhica, Mariana E.Schmitz, AnneGhidelli, MatteoSchmitz, IngeGhinassi, BarbaraSchnabel, ThomasGhisi, AldoSchnabel, UtaGhiuta, IoanaSchneider, ChristofGhoddousi, ParvizSchneider, JensGholamipour-Shirazi, AzarmidokhtSchneider, NathanaelleGholampour, SeifollahSchneider, RaphaelGholizadeh-Vayghan, AsgharSchnutenhaus, SigmarGhorbani, AliSchoeppner, VolkerGhorbani, BehnamSchohn, HervéGhorbani, FarnazSchöllhorn, BerndGhoreishi, Seyede FatemehScholtmeijer, KarinGhosh, AbhishekScholz, Konstantin JohannesGhosh, ArijitSchönemann, LarsGhosh, ArunSchöppner, VolkerGhosh, KalyanSchowalter, MarcoGhosh, MonojSchreiner, CristinaGhosh, SohamSchricker, KlausGhosh, SutapaSchröder, JörgGhoshal, SushantaSchroeder, GrzegorzGhrib, FaouziSchubert, DavidGhyngazov, Sergey AnatolievichSchulz, BernhardGiacheti, Heraldo LuizSchulz, Volker PaulGiacomello, GiovanniSchulz, WolfgangGiannakas, ArisSchulz-Siegmund, MichaelaGiannakopoulou, Panagiota P.Schwaminger, SebastianGiannakoudakis, Dimitrios A.Schwarz, UlrichGiannatsis, JohnSchwarze, MichaelGiannella, VenanzioSchwarzinger, BettinaGiannopoulos, Georgios Ι.Schwarz-Pfeiller, AnneGiannuzzi, GiuseppeSchweiss, RuedigerGianotti, ValentinaSchweizer, ThomasGiapintzakis, John (Ioannis)Schwentenwein, MartinGiardini, ClaudioSchwotzer, MatthiasGiasin, KhaledScialla, StefaniaGibbons, Gregory JohnScigliano, RobertoGibiino, Gian PieroScimemi, Giuseppe FilecciaGibot, M. PierreSciortino, AliceGierlotka, StanislawSciortino, SilvioGieroba, BarbaraSciuto, AntonellaGierszewski, MateuszScott, AlisonGierthmuehlen, Petra C.Scotti, NicolaGigante, Giovanni E.Scuderi, VivianaGil, IgnacioScuracchio, CarlosGil, Maria Luisa AlmoraimaScuro, CarmeloGili, Valerio FlavioScutelnicu, ElenaGiliopoulos, DimitriosSeara-Paz, SindyGill, Richie H.S.Sebakhy, Khaled OmarGillemot, FerencSebastian, SujeeshGilliot, MickaelSebastian, VictorGillot, FrédéricSebastian, WeberGinestra, Paola SerenaSecu, Elisabeta-CorinaGinter-Kramarczyk, DobrochnaSecu, MihailGiorgetti, AlessandroSecuianu, CatincaGiorgia, GhiaraSedelnikova, OlgaGiorgio, IvanSedev, RossenGiosuè, ChiaraSedira, NaimGiovagnoli, StefanoSedlmajer, MartinGiovannetti, RitaSedmak, AleksandarGiraldo-Gomez, DavidSedrakian, ArmenGirjob, ClaudiaSee, Chan HwangGirot, FranckSegersäll, MikaelGisbert-Garzarán, MiguelSegredo-Morales, ElisabetGiudice, FabioSegreto, TizianaGiulbudagian, MichaelSegura-Cárdenas, EmmanuelGiuliani, AlessandraSeichepine, FlorentGjörloff-Wingren, AnetteSeidlmayer, StefanGkantidis, NikolaosSeifan, MostafaGkourmpis, ThomasSeiler, PhilippGläser, RogerSeitz, BjoernGlass, Samuel V.Sejnoha, MichalGlavina, DomagojSekar, SankarGlazar, IrenaSekhar, JainageshGlazov, Alexey L.Sekiguchi, YuGlotov, AleksandrŠekularac, NenadGlovnea, RomeoSelbach, Sverre MagnusGłowacz, AdamSelech, JaroslawGlowinska, EwaSelejdak, JacekGłuchowski, AndrzejSeleznev, MikhailGłuchowski, PawełSelishchev, DmitryGlumac, Nick G.Sellegounder, DuraiGlushko, OleksandrSelles Canto, Miguel AngelGłuszewski, WojciechSellitto, AndreaGlytsis, EliasSelopal, Gurpreet SinghGnade, Bruce E.Selvaraj, Senthil KumaranGnatowski, AdamSelyutina, N. S.Gnilitskyi, IaroslavSemenov, MichaelGoanta, ViorelSemenova, ElizavetaGobis, KatarzynaSemeraro, PaolaGodbert, NicolasSemisalova, AnnaGodec, DamirSemm, ThomasGodiya, ChiragSemrád, KarolGoel, SanketSen Gupta, ArnabGoetten De Lima, GabrielSen, PratikGogolewski, DamianSen, RwikGoh, Guo DongSena-Cruz, JoseGoh, GuodongSenatov, FedorGoh, Kheng-LimSenderowski, CezaryGoh, SherminSengottuvelu, DineshkumarGohari, SoheilSenouci, AhmedGok, OzgulSensi, MatteoGokcekaya, OzkanSensini, AlbertoGokceoglu, CandanSenthil, ChenrayanGolański, GrzegorzSeo, Joon HoGolasiński, KarolSeo, Soo-YeonGołaszewski, JacekSeo, Young-KwonGoldberg, Margarita ASeokpum, KimGoldenberg, EdaSeol, Jae BokGolev, ArtemSepahvand, KheirollahGolewski, Grzegorz LudwikSepasgozar, Samad M. E.Golewski, P.Sepe, RaffaeleGolewski, PrzemysławSepúlveda Ferrer, Ranier EnriqueGolgovici, FlorentinaSepulveda, AbdonGolovina, IrynaSerafin, JarosławGolovko, O.Serani, AndreaGolub, IgorȘerban, Bogdan CătălinGomaa, Mohammed MetwallySerbanoiu, Adrian AlexandruGombar, MiroslavSerdar, UlubeyliGomes, Guilherme FerreiraSéré, GeoffroyGomes, Inês B.Seręga, SzymonGomes, JoaoSerenko, Olga A.Gómez, Clara MSerfa Juan, Ronnie O.Gomez, JulioSergi, ClaudiaGómez-Cámer, Juan LuisSergi, Pier NicolaGómez-Tena, Maria PilarSerikov, ArkadyGomonay, OlenaSério, SusanaGoncalves, AlexandreSerizawa, TakeshiGondro, JoannaSerjouei, AhmadGong, BaopingSerrà, AlbertGong, JinSerrano Rubio, AídaGong, KaiSerrano, Ricardo FernándezGong, NaSerrano-Munoz, ItziarGong, PanSesana, RaffaellaGong, PengSešek, AleksanderGong, ShiqiangSeshadri, Ramachandran ChidambaramGong, YiŠetina Batič, BarbaraGonnella, GiuseppeSetkova, Tatiana V.Gonon, MauriceSeufitelli, GabrielGonzalez Angeles, AlvaroŠevčík, RadekGonzález Nicieza, CelestinoSeverino, PatríciaGonzález Sánchez, José AntonioSevim, OzerGonzález, BegoñaSevostyanov, MikhailGonzález, EdurneSeyed Hooman, GhasemiGonzález, HaizeaSfravara, FeliceGonzález, HernánSgarbossa, PaoloGonzález, J.S.Sgobba, StefanoGonzález, SergioSgouros, Aristotelis P.González-Alonso, DavidSha, JianjunGonzález-Calbet, José M.Shaaban, IbrahimGonzález-Doncel, GasparShabbir, BabarGonzález-Garcinuño, ÁlvaroShaburova, NatalyaGonzalez-Gutierrez, JoaminShadmehri, FarjadGonzalez-Leal, Juan MaríaShafi, BrahimGonzález-Núñez, RubénShafiee, AbbasGonzalez-Pujana, AinhoaShaghayegh, BagheriGonzález-Sálamo, JavierShah, Adnan RaheelGonzalo Orden, HernánShah, Nimisha ChinmayGoodwin, Frank E.Shah, Parag KGook, SergejShah, VineetGopalu, KarunakaranShaha, SugribGopu, GopalakrishnanShahid, Muhammad KashifGóra, JacekShahmansouri, Amir AliGorbachev, IgorShahmohammadi Beni, MehrdadGorbunov, DenisShahraki, MehdiGordano, AmaliaShahriyari, FatemehGordeeva, OlgaShaik, Mohammed RafiGórecka, SylwiaShaikh, RubinaGorewoda, TadeuszShakeel, FaiyazGorgieva, SelestinaShakiba, SheydaGorjanc, Dunja ŠajnShakir, Muhammad FayzanGórny, MarcinShakor, PshtiwanGorseta, KristinaShakun, AlexandraGorshkov, NikolayShalaby, MohamedGórski, FilipShalan, Ahmed EsmailGórski, JarosławShalchi Amirkhiz, BabakGórszczyk, JarosławShamanaev, Ivan V.Goryaeva, AlexandraShamass, RabeeGorzelańczyk, TomaszShamim, NabilahGościańska, JoannaShamonin, MikhailGoswami, AnweshaShamsabadi, Elyas AsadiGoswami, SumitaShamshuddin, Mohammed D.Gottwald, AlexanderShandrikov, MaximGoudarzy, MeisamShang, GuoliangGoulas, AthanasiosShang, ZhaoGoulas, VlasiosShang, ZhongxiaGouveia, José D.Shankar Sharma, GyaniGöv, KürşadShao, JianGovindan, BharathShao, WeiGowans, EricShapovalov, VictorGoyal, ArpitShapter, JoeGoździewska-Harłajczuk, KarolinaSharaky, IbrahimGraba, MarcinSharan, PreethaGrabarczyk, RobertSharkeev, YuriGrabe, JürgenSharker, Shazid Md.Grabiec, Anna M.Sharma, AbhayGrabon, Wieslaw A.Sharma, AmitGrabski, Jakub KrzysztofSharma, AnuragGracin, DavorSharma, ArchanaGraczykowski, BartlomiejSharma, AshutoshGrădinaru, Catalina MihaelaSharma, AtulGradov, Oleg V.Sharma, DivyaGraf, ChristinaSharma, Gyani ShankarGrajek, HenrykSharma, NitinGrakovich, Petr N.Sharma, PriyankaGramlich, AlexanderSharma, Sameer KumarGranata, GiuseppeSharma, SathyashankaraGranata, Michele FabioSharma, ShubhamGrancharov, GeorgySharma, Sunil KumarGrande, AntonioSharmila V, GodvinGrandini, Carlos RobertoSharonova, Olga M.Gräning, TimShash, Ahmed YehiaGrasman, JonathanShavandi, AminGrasso, MarzioShchelokova, ElenaGrasso, SalvatoreShcherban′, Evgenii M.Grause, GuidoShchetinin, IgorGravel, EdmondShearer, CameronGrawish, MohammedShehata, NaderGray, Michael D.Sheidaei, AzadehGraziani, AndreaShekargoftar, MasoudGraziano, Sossio FabioShellaiah, MuthaiahGražulytė, JuditaShelushinina, Nina G.Grazzini, AlessandroShen, ChenGreco, AntonioShen, HuahaiGreco, GabrieleShen, JialongGreen, ItzhakShen, JianyunGreer, Andrew I. M.Shen, JinleiGrega, RobertShen, KelvinGregori, AlbertoShen, TielongGregušová, DagmarShen, TongdeGréta, GergelyShen, YongfengGrgac, PeterShen, Yu-FangGrgić, Dajana KučićShenderovich, Ilya G.Grguraš, DamirShepa, IvanGrifa, CelestinoShepherd, Jennifer H.Griffiths, JoeySher, FarooqGrigalevicius, SauliusSherif, El-Sayed M.Grigoriev, FedorShesterkina, Anastasiya AGrigoriev, PavelShestopalov, Michael A.Grilc, MihaSheu, Jinn-KongGrill, RomanShevate, RahulGrilli, NicolòShevchenko, Vitaliy G.Grillo, AlessandroShevchik, Sergey A.Grimaldi, GaiaShevtsov, SergeyGrimaudo, ValentineShi, JianhangGrimm, Tyler J.Shi, KaiyuanGrimm, Wolf-DieterShi, LeiGrinderslev, Jakob B.Shi, PengpengGRINYS, AudriusShi, Shih-ChenGrishin, MaximShi, WenjuanGrishko, VictoriaShi, YushengGriškevičius, PauliusShibayama, JunGrivel, Jean-ClaudeShichalin, OlegGrizzuti, NinoShiga, TakuyaGrochowicz, MartaShigeishi, MitsuhiroGrodzicki, MiłoszShih, Ming-ChengGromada, MagdalenaShikler, RafiGromov, Alexander AlexandrovichShill, Sukanta KumerGronowski, MarcinShim, Hyung CheoulGross, Andrew JShimada, ToshihiroGrulkowski, IreneuszShimizu, JunGrüll, GerhardShimoga, GaneshGrunwald, TimShimomura, TakeshiGruszka, KonradShimomura, TakumiGruzman, Arie-LevShin, Crystal S.Gryaznov, DenisShin, MimiGryguć, AndrewShin, MoochulGrysa, KrzysztofShin, Su-JungGryshkov, OleksandrShin, SunmiGrza̧dka, ElzbietaShin, Tea HoGrzegory, IzabellaShin, Weon HoGrzejda, RafałShin, YooseokGrzelak, KrzysztofShinde, Surendra KrushnaGrzybek, DariuszShinkai, KoichiGu, FanShinya, AkikazuGu, Grace X.Shinzato, KeitaGu, WeibingShirinbayan, MohammadaliGuan, ChengzhiShirizly, AmnonGuan, Pai-ChenShirokov, Vladimir B.Guan, RenguoShirshin, EvgenyGuan, ZeyiShirvanimoghaddam, KamyarGuarino, StefanoShiryayev, OlegGubitosa, JenniferShirzad, ShararehGudkov, SergeyShishkin, AndreiGuduric, VeraShishkovsky, Igor V.Guedes, Rui MirandaShiue, Ren-KaeGueorguiev, Gueorgui KostovShivachev, BorisGuerrero-Gironés, JuliaShiwei, YuGuerrero-Vaca, GuillermoShlyakhtin, Oleg A.Guerrieri, NicolettaShohaimi, Norshahidatul Akmar MohdGuglielmi, PasqualeShokouhimehr, MohammadrezaGuglielmi, VittoriaShokrani, AlborzGuidotti, MatteoShooshtarian, SalmanGuieu, SamuelShorstkii, IvanGuilemany, Josep MariaShrestha, NamitaGuillen, IgnacioShrivas, KamleshGujar, FarooqShtenberg, GiorgiGulati, KaranShtender, VitaliiGüler, BercesteShubbar, Ali AbdulhusseinGuler, SonerShukla, Sanjeev RamchandraGülgün, Mehmet AliShukla, ShivakantGulino, AntoninoShukrinov, YuryGulino, Emmanuel FortunatoShunmugasamy, Vasanth ChakravarthyGullon, BeatrizShurkin, PavelGülses, AydinShvalya, VasylGultom, Noto SusantoShvartsman, VladimirGumienny, GrzegorzShvidchenko, Aleksandr V.Gümrük, RecepSi, RuizheGunasegaram, Dayalan R.Siafaka, PanoraiaGünen, AliŠiaučiūnas, RaimundasGüner, Sait BarışSičáková, AlenaGunji, TakahiroSicilia, EmilaGuo, BaoSiczek, KrzysztofGuo, DandanSiddika, AyeshaGuo, FeiSiddiq, M. AmirGuo, JingSiddique, SalmanGuo, M.X.Siddiqui, MasoomGuo, QiangSiddiqui, Md. Irfanul HaqueGuo, QilinSidorenko, AlexanderGuo, QiuquanSidorenko, AnatolieGuo, RongxinSiebrecht, TobiasGuo, YannaSielker, SonjaGuo, YaoSieradzki, AdamGuo, Zhao-XiaSifakakis, IosifGupta, MohitSigle, WilfriedGupta, SumitSigmon, GingerGupta, SurajSignore, GiovanniGupta, VarunSignorini, CesareGurau, CarmelaSignorini, RaffaellaGurau, GheorgheSiirde, AndresGuraya, TeresaSik Jeon, EuyGurevich, Leonid MoiseevichSikavitsas, VassiliosGurgen, SelimSikder, PrabahaGurikov, PavelSikora, AdamGurski, VolodymyrSikora, JanuszGurung, AshimSikström, FredrikGurzhiy, Vladislav V.Silani, MohammadGusev, AlexanderSilantyeva, Elena A.Gushchin, Artem L.Sili, AndreaGuskos, NikoSili, Andrea MarianoGussone, JoachimSilikas, NikolaosGuterman, VladimirSiliqi, DritanGüth, Jan FrederikSilkin, VyacheslavGutiérrez Alonso, ÁngelSills, RyanGutiérrez, MarcosSilva, AbilioGutiérrez-Arzaluz, MirellaSilva, Enrico D.Gutiérrez-Fernández, EdgarSilva, FranciscoGutnikov, Sergey ISilva, JoãoGutzov, StoyanSilva, JorgeGuven, OnurSilva, NunoGuzmán, EduardoSilvestre, SamuelGwoździk, MonikaSilvestri, DanieleGyarmati, GáborSilyukov, OlegGyörgy, KaszaSimagina, ValentinaGyulavári, TamásSimanjuntak, Firman MangasaH. P. M. Santos, JoãoSimha Martynkova, GrazynaHa, Jung HongSimić, MitarHa, Ngoc SanSimmons, Kevin L.Habal, AyyannaSimões, F.Habbouche, JhonySimões, Fernando M.F.Haber, RodolfoSimões, PedroHabib, Md AhasanSimões, SóniaHabisreuther, TobiasSimón Marqués, PabloHabrat, WitoldSimon, AndreaHackney, PhilipSimon, VilmosHaddadi, AbbasSimonds, BrianHadermann, JokeSimonescu, Claudia MariaHadidi, MiladSimoska, OljaHadiyanto, HadiyantoSimsek, GulsuHadjadj, AomarSinar, DoganHadzik, JakubSing, Swee LeongHadzima-Nyarko, MarijanaSingh, Ajay VikramHaghbakhsh, RezaSingh, Ajit PalHagio, TakeshiSingh, AmardeepHahm, Wan-GyuSingh, DavidHahn, Jae RyangSingh, GurpreetHaider, Adawiya J.Singh, JitendraHaido, JamesSingh, Jitendra KumarHajj, RamezSingh, NeenuHajkowski, JakubSingh, Pradeep KumarHajžman, MichalSingh, PragyaHakan Ormeci, AlimSingh, S.V. BerwinHakkarainen, TuulaSingh, Sandip KumarHalama, RadimSingh, SurenderHalasi, GyulaSingh, Varun KumarHaldhar, RajeshSingla, SaranshuHale, RobertSinha, JasmineHalilovič, MiroslavSinyavsky, NikolayHall, Carl AlanSiódmiak, JacekHallan, Supandeep SinghSipos, PálHama Aziz, KosarSire, OlivierHamad, EyadSirico, AliceHamada, Atef SaadSirivolu, DushyanthHamada, ShigeruSirotin, Igor S.Hamdani, AriSiska, FilipHamdia, KhaderSiskova, AlenaHamedi, Gholam HosseinSitarz, MaciejHamedi, Hamid RezaSitnikova, ElenaHamid, Norul HishamSivakumar, SushamaHamidi, YoussefSivaramapanicker, SreejithHamilton, B. CarterSivasankaran, SubbarayanHammer, RenéSiwiec, DominikaHamouda M, MousaSiwiński, JarosławHampp, Norbert A.Siwowski, TomaszHamza, OmarŠkamat, JelenaHamzaoui, RabahSkapin, Sreco D.Han, BingSkarka, WojciechHan, BingqiangSkarzynski, Piotr HenrykHan, ChangjunSkejic, DavorHan, ChunleiSkenderidis, ProdromosHan, DaehoonSkinner, JackHan, Dong-PyoSkoczko, IwonaHan, Dong-WookSkoczylas, AgnieszkaHan, GuebumSkogby, HenrikHan, Heung NamŠkoláková, AndreaHan, Hyoung-SuSkórczewska, KatarzynaHan, KeSkordaris, GiorgosHan, WeiSkotniczny, PrzemysławHan, WenboSkouras, AthanasiosHanafi, Mohamed MusaSkouras, Eugene D.Hanafusa, HiroakiSkrabalak, GrzegorzHanafy, Nemany A. N.Skrinar, MatjazHanashima, ShinyaSkripnyak, Vladimir A.Hanc, EmilSkrodzki, PatrickHaneklaus, NilsSkrzekut, TomaszHanfi, Mohamed Y.Skrzypkowski, KrzysztofHang, ChunjinSkubisz, PiotrHang, To Thi XuanSkupov, Kirill M.Hangai, YoshihikoSkvortsov, Ivan Yu.Hanif, AsadSkvortsova, NinaHanif, Md. AbuŠlapáková, MichaelaHans Kurt, TönshoffSlatineanu, LaurentiuHanus, PavelSlávik, RichardHao, PengSlavov, StoyanHaponiuk, Jozef T.Śleboda, TomaszHaq, Mir Irfan UlŚlęczka, LucjanHaque, ArifulSleczkowski, PiotrHaque, AshanulSlepchenkov, Michael M.Hara, MasanoriSlíva, AlešHaraguchi, NaokiSlivac, IgorHaramina, TatjanaŚliwa, RomanaHarb, MohammadSlobodyan, MikhailHardy, John GeorgeSłodki, BogdanHarea, EvgheniiSlokar Benić, LjerkaHargis, Craig W.Slomka-Slupik, BarbaraHarhay, KhrystynaŚlosarczyk, AgnieszkaHarirchian, EhsanSłowik, MieczysławHariri-Ardebili, Mohammad AminŚlusarczyk, ŁukaszHaritonovs, ViktorsSmaga, MarekHarizi, WalidSmailienė, DaliaHarja, MariaSmardzewski, JerzyHarmandaris, Vagelis A.Smejda Krzewicka, AleksandraHarper, GavinŠmíd, MiroslavHarper, Jason B.Smierciew, KamilHarrison, Roger G.Śmiga-Matuszowicz, MonikaHart, Melanie L.Smirnov, Alex I.Hartl, ChristophSmirnov, AlexanderHartley, IanSmirnov, AntonHartmaier, AlexanderSmirnov, Ivan V.Hartmann, ChristophSmith, D. HudsonHartmann, PaulSmith, IanHartmann, RobertSmith, Paul F.Hasanyan, ArmanjSmolin, AlexeyHasanzadehshooiili, HadiSmolin, IgorHasegawa, GeorgeSmolina, IrinaHasegawa, MasakatsuSmolka, PetrHasegawa, NatsukiSmolka-Danielowska, DanutaHashemi, ShervinSmorygo, Oleg L.Hashemian, LeilaSmyk, EmilHashim, NorhashilaSmyrak, BeataHashimoto, MasanoriSmyrnova, KaterynaHasnat, ArifulSnopiński, PrzemysławHassan, AmerSo, ByoungjinHasse, AlexanderSoares, Bluma GuentherHatada, RurikoSoares, Paula IsabelHatakeyama, HirotoSoares, SofiaHathout, RaniaŠob, MojmírHatiegan, CornelSobianowska-Turek, AgnieszkaHattar, KhalidSobola, DinaraHattori, TomokazuSobolev, AlexanderHaupt, MichaelSobolev, ArkadijHaušild, PetrSobolev, KonstantinHavela, LadislavSobotka, PiotrHayashi, FumitakaSocalici, Ana VirginiaHayashi, HidekiSocha, TomaszHayashi, NorikoSochacki, WojciechHayashida, KenjiSoco, EleonoraHayat, FaisalSodano, FedericaHaye, EmileSoffritti, ChiaraHayes, SteveSofia, DanieleHayn, RolandSofiyev, Abdullah H.Hazell, PaulSohail, Muazzam G.He, AihuaSohn, IlHe, ChengSohn, Jeong-SooHe, GuanjieSohn, Seok SuHe, JianzhouSohn, SungWooHe, PengSokhoyan, RuzanHe, QiyuanSokolov, Andrey SergeevichHe, ShaojianSokolova, Maria P.He, ShuangSokolovsky, V. S.He, Wei-DongSokołowska, Joanna JuliaHe, WeilueSokołowski, PawełHe, ZhihaiSolano, FranciscoHeczel, AnitaSoler, DanielHeczko, MilanSoler, JordiHegab, HussienSolhjoo, SoheilHegyi, AndreeaSoliman, EslamHeibati, SeyedmohammadrezaSoliman, Mohamed ARHeiden, BernhardSolin, NiclasHeiland, MaxSolís, CeciliaHeim, FredericSolís, Luis Fernando MagañaHeinrich, BenoîtSolodov, IgorHeinze, ThomasSologubenko, AllaHejna, AleksanderSolovev, Sergei A.Helal, AasifSolovieva, AnastasiyaHelfritch, DennisSol-Sánchez, MiguelHellier, AlanSoltamov, VictorHelmi, GhanmiSoltani, NiloofarHémadi, MiryanaSoma, Arun KumarHemmatian, MasoudSoman, RohanHemprich, AlexanderSoman, SojaHenckell, PhilippSommerseth, CamillaHendrik, BargelSomogyi-Ganss, EszterHens, BartSomr, MichaelHer, Shiuh-ChuanSon, KeunBaDaHeras, FranciscoSon, Seung UkHermawan, HendraSon, Sung-aeHermerschmidt, FelixSong, BoHermoso-Orzáez, Manuel JesúsSong, GuowenHermsdorf, JörgSong, HengxuHernández, Jose AlfredoSong, In-SeokHernandez-Aldave, SandraSong, JianweiHernández-Ávila, JuanSong, JunHernández-Cocoletzi, HeribertoSong, Jung-IlHernández-Cruz, DanielSong, KaikaiHernández-Gámez, José FranciscoSong, PengHernández-Garrido, Juan CarlosSong, ShuangHernández-Montelongo, JacoboSong, Sung HoHernández-Negrete, OfeliaSong, Woo-JinHerrán, Luis FernandoSong, YuHerrero, Maria JSong, YuefengHerrmann, MathiasSongmene, VictorHertelé, StijnSood, ParveenHesebeck, OlafSoon, Jong JeongHesp, Simon A.M.Sopčák, TiborHess-Dunning, AllisonŠorli, AmritHeun, StefanSorrenti, MatteoHeyder, RodrigoSorrentino, LucaHianik, TiborSortino, MarcoHicham, LemsellemSosa, Filipe Hobi BordónHideaki, KatogiSosnowska, KatarzynaHiejima, YusukeSotiriadis, KonstantinosHigashino, TomohiroSotta, PaulHikichi, ShiroSou, KeitaroHilal, Nahla NajiSoukal, FrantisekHildebrand, JoergSoukup, RadekHilloulin, BenoitSousa, AnaHioki, HirofumiSousa, AurelianaHippler, RainerSouto, RicardoHirao, NaohisaSova, DanielaHirashita, TsunehisaSowislok, Andrea AnnaHIRATA, ToshiyukiSpadlo, AnnaHiroaki, MaedaSpagnuolo, GianricoHirschberg, CosimaŠpanić, NikolaHisada, KenjiSparnacci, KatiaHitzler, LeonhardSpasojevic, Pavle M.Hiziroglu, SalimSpassky, DmitryHlaváčová, Irena M.Spassov, TonyHlobil, MichalSpeight, James G.Hloch, SergejSperanza, VitoHo, Chia-CheSperanzini, EmanuelaHo, Duc-DuySpieckermann, FlorianHo, Son LongSpina, RobertoHo, Wen-JengSpinato, SergioHo, Yi-ChengSpinelli, GiovanniHobson, PeterSpirito, DavideHodgkinson, TomSpotorno, RobertoHodulova, ErikaSpowage, AndrewHoehnel, AndreaSprenger, StephanHoffmann, AndreasSprio, SimoneHofmann, AndreasSprynskyy, MyroslavHofmeister, AnneSreckovic, Vladimir A.Hogan, James D.Sridharan, KishoreHohmann, TimSrinivasa Rao, SunkaraHoja, JohannesSrinivasan, Dharun V.Hojná, AnnaSrinuanpan, SirasitHojo, TomohikoSrivastava, Kumar ChandanHokamoto, KazuyukiSrivastava, Rajiv RanjanHoła, JerzySrivastava, VineetHolešová, SylvaSrnec Novak, JelenaHollý, IvanSroka-Bartnicka, AnnaHolmes, RyanStachewicz, UrszulaHolschemacher, KlausStachiv, IvoHoltzer, MariuszStachowicz, FeliksHolynska, MalgorzataStagon, Stephen PeterHomaei, FarshadStaicu, TeodoraHomaeigohar, ShahinStamatelatou, KaterinaHomocianu, MihaelaStan, FeliciaHomolova, VieraStan, MirunaHomonnay, ZoltánStanca, Sarmiza ElenaHoneycutt, Wesley T.Stanciu, Magdalena CristinaHong, BinStanculescu, IoanaHong, Chang KookStander, TinusHong, Chih ChiangStanek-Tarkowska, JadwigaHong, DanfengStanica, EnacheHong, GuangStanisz, BeataHong, Ka L.Stankevic, VoitechHong, LiangStarczewska, AnnaHong, Min-HoStarikov, SergeiHong, QingguoStarke, GerhardHong, Seung-TaeStarkova, OlesjaHong, ShengStarman, BojanHonjo, HiroakiStarostenkov, MikhailHontañón, EstherStarsich, FabianHonzíček, JanStarzyńska, AnnaHoogland, DominiqueStasch, AndreasHoon Kim, SangŠťastniak, PavolHooper, Justin B.Stastny, PremyslHoppe, EdwardStathopoulos, VassilisHöppel, Heinz WernerStatnik, EugeneHorchidan, NadejdaStefan, Daniela SiminaHorkavcová, DianaStefan, KrakowiakHörn, Siegfried R.Stefan, RazvanHornak, JaroslavStefanik, AndrzejHornebecq, VirginieStefano, LettieriHornig, AndreasStefanou, GeorgeHorovistiz, AnaStefanov, BozhidarHorovistiz, Ana LúciaSteffi, ChrisHorowitz, Robert A.Stegmaier, ThomasHorszczaruk, ElżbietaSteinbacher, MatthiasHortigüela, María J.Steiner, DarjaHorwitz, JacobSteinert, PhilippHošek, JanSteinfeldt, NorbertHosen, AkterStel’makh, Sergey A.Hoshiar, Ali KafashStelmach, EmiliaHossain, Md. ShahadatStelmach, SlawomirHossain, MokarramStempkowska, AgataHossain, NaziaStěnička, MartinHossain, RumanaŠtěpánek, FrantišekHossain, ShakhawatStepanov, NikitaHossein Roshani, GholamStepanova, Ekaterina N.Hossein, MonajatiStephan, SimonHossein, PayamStepien, AnnaHosseinnezhad, MozhganStępień, MichałHosseinpoor, MasoudStepinac, MislavHosseinpour, SamanStergioudi, FaniHosseinpour, SepantaStern, AdinHossfeld, MaxStern, RaivoHotza, DachamirStevulova, NadezdaHou, DewenSthal, FabriceHouache, MohamedStielkowski, WadimHoujou, HirohikoStiharu, IonHouska, JiriStirner, ThomasHoyer, Kay-PeterStiubianu, GeorgeHrcek, SlavomirStoch, PawełHristov, IlianStochino, FlavioHristu, RaduStock, StuartHryniewicz, TadeuszStodolak, EwaHsia, Shao-YiStoenescu, DanielHsiang, Hsing-IStogniy, Marina Yu.Hsiao, Hsi LienStoica, AnicutaHsiao, Shih-NanStoilova, OlyaHsiao, Ta-ChihStojek, ZbigniewHsieh, Chien-MingStojmenović, MarijaHsieh, Sung-ChihStoklas, RomanHsini, AbdelghaniStolarczyk, AgnieszkaHsissou, RachidStolarczyk, KrzysztofHsu, Cheng-HsunStolarski, AdamHsu, Chia-JuiStolbov, OlegHsu, Chih-NengStommel, MarkusHsu, Day-ShinStopic, SreckoHsu, Fu-YinStorchak, MichaelHsu, Quang-CherngStorsberg, JoachimHsue, Albert Wen JengStöwe, KlausHu, AnmingStoyan, DietrichHu, ChengliangStradomski, GrzegorzHu, ChengzhiStrasser, VidaHu, ChuanshuangStratil, LudekHu, ChunStraumal, Alexander B.Hu, FuwenStraumal, BorisHu, GuobiaoStrauss Rambo, Dimas AlanHu, Guo-QingStrazdienė, EugenijaHu, HongliŠtrbac, SvetlanaHu, HuanStrečka, JozefHu, JianqiaoStrelec, StjepanHU, JIEStrelets, KseniaHu, Jin-JiaStrianese, MariaHu, JinlianStrielkowski, WadimHu, JunlingStringaro, AnnaritaHu, KuanStrnad, RadekHu, ShanshanStróż, DanutaHu, Wei-PingStrušnik, DušanHu, XiaofeiStruzik, MichalHu, YangStruzikiewicz, GrzegorzHu, Yong-JieStrzałka, DominikHu, ZhongweiStrzałkowski, KarolHuang, ChiayiStrzelecki, PrzemysławHuang, Chien WeiStudziński, RobertHuang, Chih-ChiaStuhr, UweHuang, Chih-FengStular, DanajaHuang, Chun-YingSturm, RomanHuang, DaliStylianakis, Minas M.Huang, DiSu, Chie-ShaanHuang, DongshengSu, Chiung-WuHuang, Haw-MingSu, CongHuang, Heng LiSu, JingHuang, KaiwuSu, MeiniHuang, KevinSu, YishiHuang, MingSu, ZhouchengHuang, Po-JungSuarato, GiuliaHuang, QijinSuarez Bermejo, Juan CarlosHuang, QiuliangSuárez, AlfredoHuang, Ren-YeongSuárez, FernandoHuang, TaizhongSuarez, Maria JesúsHuang, WeiminSuárez-García, SalvioHuang, Wen ChangSuárez-López, María JoséHuang, Wu-JangSuárez-Macías, JorgeHuang, YanliangSubbiahdoss, GuruprakashHuang, ZhaohuiSubbotin, KirillHuang, ZhongjieSubianto, SuryaHubálková, JanaSubramaniyan, PrabhakaranHučko, BranislavSubroto, TungkyHuda, NazmulSuchaneck, GunnarHudec, PavolSucharda, OldrichHuebsch, NathanielSuchea, MirelaHueso, Jose L.Suchkov, A. N.Huettig, FabianSuchkov, Alexei N.Hughes, Anthony E.Suchorzewski, JanHughes-Riley, TheodoreSuess, Ryan J.Huh, Do SungŠugár, PeterHulimka, JacekSugawara-Narutaki, AyaeHulme-Smith, Christopher NeilSugii, HidekiHülsmann, MichaelSugimoto, KohichiHumad, Abeer M.Suh, Jin-YooHumbert, Pedro SilvaSuh, Jung-IlHumelnicu, DoinaSuhito, Intan RosalinaHumphries, TerrySuhonen, TomiHumphry-Baker, SamuelSujak, AgnieszkaHung, Fei-YiSukegawa, ShintaroHung, KuofengSukhikh, TaisiyaHung, Tai-FengSukumaran, VijayHunter, Bryan M.Sulaeva, IrinaHuntosova, VeronikaŠulák, IvoHuo, SiqiSulekar, SoumitraHuo, SuguoSuliga, MaciejHur, Do HaengSulka, Grzegorz DariuszHusár, JozefSułowicz, SławomirHuseien, Ghasan FahimSułowska, JustynaHušek, MartinSultan, Mohamed Thariq Bin Haji HameedHüser, DorotheeSumesh, ArrangotHussain, AtifSun, BaochangHussain, AttifSun, ChuanyuHussain, FayazSun, HaidingHussainova, IrinaSun, Hao-LingHussein, RamySun, HongbinHuynen, IsabelleSun, HongjuanHuynh, Thanh-CanhSun, I-WenHuzlik, RostislavSun, JialinHvizdoš, PavolSun, JingyaoHwang, ByungilSun, JinhuaHwang, David J.Sun, JunboHwang, Hyeon-JongSun, MengtaoHwang, JoongkiSun, QingchaoHwang, Jung-hwanSun, ShaolongHyer, HoldenSun, ShuqingIacoviello, FrancescoSun, Wei-QiangIaculli, FlaviaSun, WenjuanIan, StroudSun, YuanyuanIandolo, AlfredoSun, YuqingIannace, GinoSun, ZengqingIannazzo, DanielaSun, ZhuangzhiIannitti, GianlucaSundar, V. S.Iannuzzo, AntoninoSundaram, PaulIatrou, HermisSundarrajan, SubramanianIatsunskyi, IgorSung, HyokyungIbáñez-Fonseca, ArturoSunil, PathakIbáñez-Forés, ValeriaSuñol, Joan-JosepIbarra, RicardoSunyol, JoanIbarra-Castanedo, ClementeSuo, JinpingIbragimov, RuslanSurace, CeciliaIbrahim, Maria SalemSurace, RossellaIchimura, MasayaSuraneni, PrannoyIdir, RachidaSurdu, Vasile AdrianIezzi, GiovannaSurdu-Bob, CristinaIgarashi, GoSuresh, KalidassIglesias, EmiliaSurmenev, RomanIglesias-Martínez, EmiliaSurmenev, Roman A.Iglev, HristoSurowka, PiotrIglic, AlesSurowska, BarbaraIgnjatović, IvanSurženkov, AndreiIguchi, FumitadaSushkova, SvetlanaIkeda, MasahikoSušić, AleksandarIkeda, MasaomiSuszanowicz, DariuszIkeda, TakayukiSutlović, AnaIkegami, MasashiSutton, Adam T.Ikenoue, TakumiSuvorov, Dmitriy V.Ikram, AwaisSuwardi, AdyIkumapayi, OmolayoSuyama, Daniel IwaoIl’yushin, Mikhail A.Suzuki, SeiyaIlankoon, SamanSuzuki, TohruIldikó, TarSuzuki, TomoIlg, PatrickSuzurikawa, JunIlić, TanjaŠvábenská, EvaIliescu, MihaielaŠvajlenka, JozefIliopoulos, IoannisSvavarsson, Halldor GudfinnurIljkić, DarioSvec, PeterIllés, BalázsSveda, MariaIllia, DobrydenŠvedienė, JurgitaIlluzzi, GaetanoSvensson, Ann MariIlnicka, AnnaSvintsov, Alexander P.Iluţiu-Varvara, Dana-AdrianaSvoboda, JiriIlyas, SadiaSvoboda, KurtImamura, GakuSwails, NahidImhof, WolfgangSwami Vetha, Berwin SinghImperatore, StefaniaSwearer, DayneImran, JamalSweat, RebekahImre, BalázsŚwiderski, WaldemarInagaki, NorihiroŚwięch, DominikaInazumi, ShinyaSwiegers, GerhardInbraj, StephenŚwiercz, RafałIndrajit, RayŚwierczek, JanInfante-García, DiegoŚwierczyńska, AleksandraInoishi, AtsushiŚwietlik, RomanInoue, Shin-IchiSwinarew, AndrzejInoue, TadanobuSwindle, AndrewInukai, JunjiSwirad, SlawomirInvernizzi, StefanoŚwit, GrzegorzIoannides, TheophilosSydor, MaciejIoannidou, Melina P.Sykora, DavidIoelovich, Michael JacobSyngouna, VasilikiIon, Rodica-MarianaSynoradzki, KarolIonete, Eusebiu IlarianSypniewska-Kaminska, GrażynaIonita, DanielaSysel, PetrIordache, Ana-MariaSysoev, Vitalii I.Iovkov, IvanSyta, ArkadiuszIpus, Jhon J.Szabados, MártonIqbal Faruque, Mohammad RashedSzabó, Bence TamásIqbal, FaizSzabó, Peter J.Iqbal, MuhammadSzabó, TamásIqbal, NaveedSzabó-Bárdos, ErzsébetIrani, MissamSzachogluchowicz, IreneuszIrico, SaraSzadkowski, BolesławIrie, MasaoSzadkowski, JanIrina, CarlescuSzafraniak-Wiza, IzabelaIrshad, AhamedSzajnar, JanIrshad, KashifSzaller, ZsuzsannaIrvin, JenniferSzałowski, KarolIsaev, AbdulgalimSzamałek, KrzysztofIsalgue, AntonioSzarek, ArkadiuszIsasti, NereaSzatyłowicz, EwaIshfaq, KashifSzczepanik, BeataIshida, TamaoSzczes, AlexandraIshige, RyoheiSzczesniak, KatarzynaIshihara, RyoSzczucka-Lasota, BożenaIshihara, SotomiSzekely, GyorgyIshii, DaisukeSzekeres, Anna MariaIshikawa, TadahikoSzer, JacekIshimoto, TakuyaSzewczuk-Karpisz, KatarzynaIshitani, HaruroSziebig, GaborIshutov, SergeySzielasko, KlausIskander, MaguedSzilagyi, HenrietteIslam, Md RashadulSzkarowski, AlexanderIslam, MohammadSzklarzewicz, JanuszIslam, Mohammad NurulSzkliniarz, AgnieszkaIslam, SaifulSzkodo, MarekIsland, JoshuaSzlancsik, AttilaIsmadji, SuryadiSzłyk, EdwardIsmagilov, Rinat R.Szmytkowski, JȩdrzejIsmaiel, AdnanSzota, MichałIsola, GaetanoSzota, PiotrIsopescu, Dorina NicolinaSzubert, KarolIsotahdon, ElisaSzukalski, AdamIspas, AdrianaSzuki, MichioIstrate, BogdanSzukiewicz, RafałIstván, HornyákSzumiata, TadeuszIsufi, BrisidSzurgacz, DawidIsvoranu, DragosSzwagierczak, DorotaIten, MurielSzybowicz, MiroslawItina, Tatiana E.Szymańska, Iwona B.Ito, AkitakaSzymańska, JolantaIto, KazuhiroSzymborski, TomaszIto, KiyohiroSzymczyk, AnnaIto, MikioTa, Hang ThuIto, SeigoTabaković, AmirIto, ShunichiroTaboada-Serrano, PatriciaIto, TsutomuTacias-Pascacio, Veymar GuadalupeIván, BélaTadesse, Melkie GetnetIvančević, DarkoTadin, AntonijaIvanić, IvanaTadyszak, KrzysztofIvannikov, A. Yu.Tafarojnoruz, AliIvannikov, AlexanderTagantsev, DmitryIvanov, MaximTaghizadeh, MohsenIvanov, OlegTaglietti, Angelo MariaIvanov, VictorTaguet, AurélieIvanov, VitaliiTahami, Seyed AmidIvanov, Vladimir K.Taha-Tijerina, José JaimeIvanova, AllaTahir, Asif AliIvanova, BojidarkaTahmoorian, FarzanehIvanova, Dimka I.Tailhades, JulienIvanovich, Tyurin YuryTait, NiallIvask, AngelaTajiri, NaokiIvetic, TamaraTakabatake, KiyofumiIvošević, ŠpiroTakacs, GaborIvšinović, JosipTakada, KenjiIwamoto, TakeshiTakahashi, HidenoriIwaniszyn, MarzenaTakahashi, KojiIwański, MarekTakahiro, KatsumiIwański, MateuszTakahiro, KawamuraIwaoka, MichioTakaki, KoichiIwasa, TakashiTakali, FaridIwaszko, JozefTakamura, MasatoIwata, TatsuyaTakano, CyroIzak, PiotrTakashima, MaiIždinský, JánTakashima, ToshihiroIzquierdo, BorjaTakayama, OsamuIzu, NoriyaTakeda, TomotakaJabłońska, KrystynaTakenaka, MikihitoJackiewicz-Rek, WiolettaTakeshita, TatsuyaJackson, MarkTakesue, NaohisaJacobs, Jordan H.Taki, KentaroJadhav, Suraj DinkarTaki, MakotoJadidi, KazemTakizawa, ShinobuJadidi, MehdiTaktak, MohamedJadwicienczak, WojciechTaleb, AbdelhafedJafarinejad, ShahryarTalebizadeh Sardari, PouyanJafarzadeh, SiavashTalimian, AliJaffe, MichaelTalneau, AnneJagadesh, P.Talreja, NeetuJahanbakhsh, HamidȚălu, ȘtefanJahandideh, ForoughTalukder, Mamunur RashidJahangiri, FazelTalwar, SteffiJain, AbhaTamagawa, HirohisaJain, DharamdeepTamarin, OllivierJain, HemantTamarit, Josep LluísJain, VarshaTamas, OllarJaiswal, DevinaTamas-Benyei, PeterJaiswal, SwarnaTamayo, AitanaJakes, Joseph E.Tamburini, DiegoJakić, MićeTamm, TarmoJakovac, MarkoTammana, Sree Rama ChandraJakubas, AdamTampa, MirceaJakubczak, PatrykTamuliene, JelenaJakubovskis, RonaldasTamura, ShoichiJakubowicz, JarosławTamus, Zoltán ÁdámJakubowski, WitoldTan, Chee-KeongJalali, KamalTan, Noel PeterJalil, AamirTan, QiyangJałochowski, MieczysławTan, YuyeJamaludin, Khairur RijalTanadchangsaeng, NuttapolJambor, MichalTanaka, HiroJamil, Muhammad AhmadTanaka, Hiroyoshi Y.Jammalamadaka, UdayabhanuTanaka, KoichiJamon, DamienTanaka, MasatoJamroziak, KrzysztofTanaka, RayJamrozik, WojciechTanaka, RyouJan, SuchorzewskiTanase, ConstantinJanagam, Dileep R.Tandecka, KatarzynaJanakiram, SaravananTandon, VivekJanal, Malvin N.Tandzi, Liliane N.Janas, DawidTanemossu Fobofou, Serge AlainJančić Heinemann, RadmilaTang, Chao-WeiJancikova, SimonaTang, Cheng-MingJańczewski, DominikTang, ChongchongJanda, TomasTang, ShiJandaghi, Mohammad RezaTang, YongbingJandrlić, IvanTang, YumingJaneliukstis, RimasTangl, StefanJang, ChangheuiTangoulis, VassilisJang, Keon-SooTania Prontera, CarmelaJang, Sung-HwanTanikawa, WataruJaníček, PetrTanks, JonathonJanik, MiroslawTao, ChengchengJanikowska, GrażynaTao, PengJankauskaite, VirginijaTao, ShunyanJankauskas, VytenisTapoglou, NikolaosJankowiak, TomaszTarantino, GabriellaJankowski, NicholasTarasiuk, WojciechJannesari, ReyhanehTarasov, AndreiJanovák, LászlóTarasov, Anton S.Janovec, JozefTarasov, IvanJansen, InekeTarasov, Sergei YuJansto, Steven G.Tarditi, Ana MariaJanulionis, RemigijusTarrés, QuimJanuševičius, KarolisTashiro, ShinichiJanusz, KozubalTashiro, ShoheiJanuszewicz, BartłomiejTashlykov, Oleg LeonidovichJanuszewski, RafałTaskinen, PekkaJanutka, AndrzejTata, Maria ElisaJaqueline Kaschuk, JoiceTataranni, PiergiorgioJaras, ArūnasTatarko, PeterJaroń, TomaszTateishi, IkkiJarosz, TomaszTavares, Carlos Jose MacedoJarzabek, DariuszTavares, S.M.O.Jasenka, Gajdoš KljusurićTavkhelidze, AvtoJasińska, IzabelaTawade, Bhausaheb V.Jasinski, RadoslawTawani, ArpitaJaśkiewicz, KarolTawfik, TaherJaskulski, RomanTayal, ShubhamJastrzębska, IlonaTaydakov, IlyaJavadian, HamedrezaTayeb, Ali H.Javed, HassanTayeh, Bassam A.Javed, MuhammadTaylor, AdamJaved, Muhammad FaisalTayyab Noman, MuhammadJavidani, MousaTchetina, ElenaJavier Baeza, FranciscoTchewonpi, Sorel SaguJaworska, EwaTchuindjang, Jérôme TchoufangJayababu, NagabandiTealdi, CristinaJayakumar, AnjaliTeasdale, IanJayakumar, JayachandranTęcza, GrzegorzJean Philippe, PérezTeghil, RobertoJedlovszky-Hajdu, AngelaTeixeira Coelho, ReginaldoJędrysiak, JarosławTeixeira, Elisabete R.Jędrzejewska, AgnieszkaTeixeira, Guilhermina FerreiraJedynak, KatarzynaTeixeira, Paulo I. C.Jeeravipoolvarn, SilawatTeixeira-Santos, RitaJeffs, SpencerTejani, GhanshyamJelagin, DenisTekin, Huseyin OzanJeleń, PiotrTekle, BirukJena, Himanshu SekharTekle, Biruk HailuJena, Sandeep KumarTekumalla, SravyaJencova, VeraTelegdi, JuditJendrzejewska, IzabelaTelegin, Andrey V.Jeng, Jiiang-HueiTeleki, AlexandraJeng, Ming-JerTeleszewski, TomaszJeng, Sheng-LongTelfah, AhmadJeng, Yeau-RenTenchov, BorisJenkins, DavidTeng, Kah HouJenko, MonikaTenkumo, TaichiJenny, Titus A.Tenorio-Alfonso, AdriánJensen, MikaelŢenu, IoanJenuš, PetraTeodor, VirgilJeon, HojeongTeodoriu, CatalinJeon, Ie-RangTeodosiu, CarmenJeon, Jae-DeokTeotia, Arun KumarJeon, Ju-WonTera, MelaniaJeon, Seog-JinTercjak, AgnieszkaJeong, JaeminTerek, PalJeremic, DejanTerenzi, AndreaJevnikar, PeterTereygeol, FlorianJezierski, JanTer-Ovanessian, BenoîtJezowski, PawelTerpiłowski, KonradJha, RajveerTerrones-Saeta, Juan MaríaJhatial, Ashfaque AhmedTerzan, JanvitJi, GuozhaoTerzopoulou, ZoiJi, ShaofeiTesar, JiriJi, VincentTesfaye, FisehaJia, XiaolongTeslyuk, VasylJia, YanminTESSIER, Pierre-YvesJian, Bo-LinTesta, GabrielJian, ShunyiTestani, ClaudioJiang, ChengminTeterycz, HelenaJiang, Cho-PeiTetsushi, MatsudaJiang, GuoTetuko, Anggito P.Jiang, JiantangTexier, DamienJiang, JiekeTezok, FatihJiang, MengThabet, AhmedJiang, MingThackray, RichardJiang, PengThakkar, HarshulJiang, RuinianThakur, Vijay KumarJiang, ShaohuaThalmaier, GyörgyJiang, WenmingThamaraiselvan, ChidambaramJiang, ZaixingThangavel, GurunathanJiang, ZiwenThangavel, RanjithJianyu, ZhouThanikodi, SathishJimenez Ballesta, Ana EvaTheocharidou, AnnaJiménez Millán, JuanTheodorakis, PanagiotisJiménez, AmaiaThiebault, ThomasJiménez, AmaliaThiele, KlausJimenez, CatalinaThierry, ToupanceJiménez, JoséThirumalai, DinakaranJin, Chang-HyunThiruppathi, Antony R.Jin, CongruiThiyagarajan, Jothi SaravananJin, HuanyuThoden Van Velzen, UlphardJin, WenchengThomas, BramlJindo, KeijiThomas, Selvin P.Jing, XiaThomas, SibyJinoop, A.N.Thuault, AnthonyJiri, OravaThumsorn, SupaphornJirka, IvanThuvander, MattiasJirková, HanaTiago, GonçaloJirsák, OldřichTian, GuocaiJmerik, ValentinTian, YebingJo, ChangshinTian, ZhenhuaJo, IlgukTibbetts, Katharine MooreJo, SumiTiberiu Petrescu, Florian IonJoanna, Borowiecka-JamrozekTiburcio, CitlalliJoannès, SébastienTie, YingJoão Ramalhosa, MariaTietze, RainerJoão, SilvaTiferet, EitanJohnson, Casey P.Tiggelaar, Roald M.Johnson, MichaelTihkonov, VladimirJohnson, ShaneTiliakos, AthanasiosJohnston, BrianTimmins, PeterJohnston, Michael J. W.Timon, RabczukJokić, Nataša IvančićTimoumi, AbdelmajidJones, James F. X.Tinga, TiedoJones, RhysTinoco, JoaquimJoo, HwajooTirth, VineetJoo, Sung-MinTishkevich, DaryaJoos, JonasTita, OvidiuJopek, HubertTito, AnnalisaJorcano, Jose LuisTitov, DmitriyJordan, HristovTiuc, Ancuţa ElenaJosé, Vidal-GancedoTiwari, Anand P.Joseph, JohnTiwari, Arjun PrasadJoseph, OlufunmilayoTiwari, PankajJoseph, PaulTkach, ItshakJoshi, BhupendraTkach, OleksandrJoshi, GargiTkacheva, OlgaJoshi, NiravTkalec, UrošJoshi, RakeshTkavc, RokJoshi, SameerTobaldi, David MariaJoshi, ToyanathTobola, DanielJoshi-Imre, AlexandraTobrman, TomášJotautiene, EgleTocci, ElenaJoubaneh, Eshagh FarzanehTodorov, RossenJouiad, MustaphaTodorov, TodorJoun, MansooTofangchi, AlirezaJovanović, SvetlanaTofanica, Bogdan MarianJovičević-Klug, MaticToker, Guher P.Jovicevic-Klug, PatriciaToko, KiyoshiJoyklad, PanuwatTolidis, KosmasJozić, DražanTolosana, EduardoJozic, SonjaTolstoguzov, AlexanderJóźwiak, TomaszToma, Ionut OvidiuJozwiak-Niedzwiedzka, DariaToma, Stefan-LucianJuárez-Hernández, ArturoTomala, Agnieszka MariaJucius, DaliusTomašegović, TamaraJug, JasminTomasiunas, RolandasJugessur, AjuTomašovičová, NatáliaJukić, SilvanaTomastik, JanJuknelevičius, RomualdasTomaszewicz, ElżbietaJulian, FernandoTomaszewska, JolantaJulien, Christian M.Tomaszewski, DanielJung, ChristianeTomaszewski, TomaszJung, Doo-HwanTombros, Stylianos F.Jung, Gang SeobTomchuk, OleksandrJung, Hyun WookTomków, JacekJung, HyunsookTomokiyo, YoshitsuguJung, Ki JinTomšič, BrigitaJung, Min-CherlToncheva-Moncheva, NataliaJung, MinsuTondi, GianlucaJung, OleTonelli, DanielJungnickel, ChristianTonelli, MonicaJuodkazis, SauliusTonezzer, MatteoJuodkazytė, JurgaTong, LigeJuoksukangas, JanneTong, MingmingJuradin, SandraTong, Van-CanhJurašin, Darija DomazetTong, YexiangJurczak, RobertTönjes, AnastasiyaJurczyszyn, KamilTonti, AndreaJurdana, VedranTopličić Ćurčić, GordanaJurj, Ancuţa MariaTopolski, AdrianJuskenas, RemigijusToribio, JesúsJuskowiak, BernardTornabene, FrancescoJust, AlarTornari, ViviJůza, JosefToro, NormanJůzl, MiroslavToro, Roberta G.Jyoti Bora, PritomTörök, JozefK. Sharma, SunilTörök, TamásKa Ming, TamTorop, JannoKabanos, ThemistoklisTorre, RobertoKabashi, NaserTorrens-Serra, JoanKabatcc, JaninaTorres Vaamonde, José EnriqueKabir, S.M. FijulTorres, CristianKaca, WiesławTorres, HectorKacalak, WojciechTorres, Javier MartínezKacianauskas, RimantasTorresani, ElisaKącka-Zych, AgnieszkaTorres-Lagares, DanielKaczmarek, SławomirTorres-Luque, MagdaKaczmarek, WojciechTorres-Mendieta, R.Kaczyński, PawełTorres-Rodríguez, MiguelKada, SitaramaTorres-Torres, CarlosKadam, AvinashTorzewski, JanuszKadam, VinodToscano, ManuelKader, SafaaTošner, ZdeněkKadhim, Majid M.A.Tosoni, SergioKadi, NawarTotan, Alexandra RipszkyKadivar, EbrahimToth, Andras JozsefKadokawa, Jun-ichiToth, BalazsKaduk, JamesTóth, Tivadar M.Kadumudi, Firoz BabuToth-Katona, TiborKaewunruen, SakdiratTotu, EugeniaKafexhiu, FevziToudert, JohannKageshima, YosukeToufigh, VahabKagkoura, AntoniaTräbert, ElmarKaichev, V. V.Traini, ToninoKairytė, AgnėTrajkovic, JelenaKaiser, Mohammad SalimTran, Anh VyKajitani, TakashiTran, Lam Thi NgocKakati, NitulTran, Quang AnhKakimoto, KoichiTran, Van-ThaiKaklauskas, GintarisTrante, OctavianKakur, NareshTrawiński, BartoszKalácska, GáborTrejbal, JanKálai, TamasTreutler, KaiKalappan, BalachanderTrevlakis, Stylianos E.Kalashnikov, KirillTrhlíková, OlgaKalashnikova, TatianaTri Phung, QuocKalatehjari, RoohollahTriantafyllou, SavvasKaliappan, SenthilTriantis, Dimos A.Kalinowska Lis, UrszulaTribelsky, MichaelKalligeros, Stamatis S.Tribst, João Paulo MendesKalliokoski, MattiTridello, AndreaKalluru, PolirajuTrif, MonicaKalmár, JózsefTrindade, AnaKalogeropoulos, GeorgeTrindade, BrunoKalogirou, AndreasTrindle, CarlKalosakas, GeorgeTrinh, Minh-ChienKalsar, RajibTrinh, ThuatKalska-Szostko, BeataTripathi, NishantKaltzoglou, AndreasTrník, AntonKalyana Sundaram, Ramalingam VenkatTrofimov, EvgenyKamaraj, Abishek B.Troiani, EnricoKamebuchi, HajimeTronci, GiuseppeKamedulski, PiotrTrovalusci, PatriziaKamel, KarolTrpčevska, JarmilaKamenev, KonstantinTrubia, SalvatoreKameyama, AtsushiTrucillo, PaoloKaminari, ArchontiaTrujillano, RaquelKaminskas, RimvydasTruong, Gia ToaiKaminski, KamilTruong, Nghia P.Kaminski, MeghanTrusek, AnnaKaminski, PiotrTrushnikov, DmitriyKamiya, OsamuTruster, TimothyKamkar, MiladTrybala, AnnaKämmerer, Peer W.Trybek, GrzegorzKamperidou, VasilikiTrytek, AndrzejKamysz, ElżbietaTrzcinski, JerzyKan, WenhaoTrzebicka, BarbaraKanakubo, ToshiyukiTrzepiecinski, TomaszKanasaki, MasatoTsai, HsushengKanaujiya, JitendraTsai, Meng-HsiuKanazawa, KenTsai, Meng-TingKandadai, NirmalaTsai, Ming-HsuKandan, K.Tsai, Yun-TingKandelbauer, AndreasTsakmakidis, Kosmas L.Kandemir, AliTsangouri, EleniKandhasamy, SathiyarajTsantzalis, StavrosKaneko, KenjiTsao, Chung ChenKanematsu, HideyukiTsavdaridis, Konstantinos D.Kanerva, MikkoTsave, OlgaKang, ChanKyuTsay, CHien-YieKang, ChaoTsay, Leu-WenKang, ChengweiTscheliessing, RupertKang, ChoonghyunTseghai, Granch BerheKang, HyeonggonTselev, AlexanderKang, Hyun-wooTseluikin, VitalyKang, JidongTseng, Shi-ChangKang, MinjungTsepelev, VladimirKang, Moon-sungTsikourkitoudi, VasilikiKang, Norbert VenantiusTsilipakos, OdysseasKang, Sung-HoonTsioulou, OuraniaKang, SungwookTsirka, KyriakiKangal, Murat OlgaçTsitsilianis, ConstantinosKangas, HeliTsivintzelis, IoannisKania, HenrykTsoi, James Kit-honKania, TomaszTsongas, KonstantinosKaniewska, KlaudiaTsouknidas, AlexanderKanjilal, BaishaliTsuchida, HidetsuguKannan, A. RajeshTsui, TingKannan, RangasayeeTsuji, HidetoKanno, TakahiroTsukamoto, ShoKano, NaokiTsukegi, TakayukiKanzow, PhilippTsutsui, SatoshiKao, Chai-LinTsvetkov, MartinKao, Yung-ChouTsvetkov, Nikolai V.Kapcia, Konrad JerzyTsygankova, LiudmilaKapelyushin, YuryTsyntsarski, BoykoKaplan, MichaelTu, Ming-GeneKaplan-Ashiri, IfatTubaro, CristinaKaplar, RobertTubío, Carmen R.Kar, Rajiv KumarTucci, FaustoKar, SabyasachiTuccitto, NunzioKara De Maeijer, PatriciaTuci, GiuliaKarahaliou, PanagiotaTucker, NickKarahan, MehmetTufail, Rana FaisalKarakalas, AnargyrosTulliani, Jean-MarcKarakoç, AlpTuma, DirkKarakouzian, MosesTumkin, Ilya I.Karaliūnas, MindaugasTumurugoti, PriyathamKaratasios, IoannisTun, Khin SandarKarathanasopoulos, NikolaosTunakova, VeronikaKaravai, OlgaTunes, Matheus A.Karavasili, ChristinaTung, Nguyen ThanhKarayannis, Chris G.Tuovinen, TeroKarczemska, AnnaTurco, RosaKardas, EdytaTurek, ArturKareiva, AivarasTurek, PawełKarfaridis, DimitriosTurk, GoranKargozar, SaeidTuttle, BlairKarim, NazmulTuttobene, Marisel RominaKarimi-maleh, HassanTuzimski, TomaszKarimipour, ArashTverdokhlebov, SergeiKarimov, ArturTwardowska, AgnieszkaKarimov, DenisTwardowski, PawełKariya, ShotaTwu, Ruey-ChingKariz, MirkoTyflopoulos, EvangelosKarková, MonikaTyrologou, PavlosKarl, Christian WolfgangTyschenko, Ida E.Karlík, MiroslavTyurin, Anton P.Karmakar, AnwesaTyutin, MaratKarmiris-Obratański, PanagiotisTzani, AndromachiKarnati, Konda ReddyTzetzis, DimitriosKärnfelt, CamillaUbaidullah, MohdKarolczuk, AleksanderUbong, EtimKaroutsos, VagelisUbysz, AndrzejKarparvarfard, AliUceda, Antonio LopezKarpenkov, DmitriyUceda-Rodríguez, ManuelKarri, Rama RaoUchaikin, SergeyKarvounis, ArtemiosUddin, Md. NizamKaseem, MosabUde, AlbertKaselouris, EvaggelosUdroiu, RazvanKasemer, MatthewUdvardy, AntalKasemsiri, PornnapaUeji, RintaroKashani, HamzehUekusa, HidehiroKashif, MuhammadUeng, Shyh-KuangKashkarov, Egor B.Ueno, TomonagaKashyzadeh, Kazem RezaUeshima, NobufumiKasikov, Aleksandr G.Uetani, KojiroKasoju, NareshUhlemann, JörgKasperski, JacekUhrlandt, DickKasprzak, ArturUjah, Chika OliverKasprzhitskii, AntonUkrainczyk, NevenKasprzyk, PaulinaUl Ahad, InamKastrinaki, GeorgiaUlasov, IlyaKastyl, JaroslavUlatowska-Jarża, AgnieszkaKasymov, DenisUlbin, MiranKaszyca, KamilUlewicz, RobertKatančić, ZvonimirUline, Mark J.Kathirvel, PreethiUlucan Karnak, FuldenKatic, VisnjaUm, Doo-SeungKato, ShinyaUm, Nam IlKato, TakamitsuUmar, KhalidKats, Victor M.Umerska, AnitaKatunin, AndrzejUmetsu, Rie Y.Katz-Demyanetz, AlexanderUngureanu, CameliaKatzer, JacekUnno, MasafumiKaufhold, StephanUnzueta, IraultzaKaul, PeterUpare, Pravin P.Kaur, GagandeepUporov, Sergey AlexandrovichKaushal, VinayakUppu, RavitejKaushik, Nagendra KumarUr, Soon-ChulKavadiya, ShalineeUra, SharifuKavaz, EsraUrase, TaroKawaai, KeiyuUrbanek, PavelKawai, ToshiyukiUrbanowicz, MarcinKawalec, JacekUrbańska, Weronika MartynaKawalek, AnnaUrbas, RašaKawałek, Anna MałgorzataUrbikain Pelayo, GorkaKaya Özdemir, DeryaUrbikain, GorkaKazakov, Alexander A.Ureña, JuliaKazama, MotokiUrieta, JoséKazanskiy, NikolayUrriolabeitia, EstebanKazantseva, NataliyaUrsic, HanaKaze, Cyriaque RodrigueUrsu, LauraKazek-Kęsik, AlicjaUsanova, KseniiaKazimierczak, PaulinaUsca, Üsame AliKazior, JanUseche, J.Kazmi, SyedUshakov, Arseni V.Kazmi, Syed Minhaj SaleemUshida, AkiomiKazukauskas, VaidotasUskoković, VukKazuma, HigashisakaUspal, William E.Kchaou, MohamedUstunel, SenayKe, HuaUsui, HidetomoKeawsawasvong, SuraparbUsui, HiroyukiKechagias, JohnUtkin, AndreiKeita, EmmanuelUtsunomiya, ToruKelarakis, AntoniosUTU, Ion-DragosKelley, StevenUva, GiuseppinaKelly, CatherineUvarov, NikolayKemmegne-Mbouguen, Justin ClaudeUzunov, IvanKenanakis, GeorgeVaclav, KotlanKenichi, SatoVaclavik, VojtechKenny, AmitVadahanambi, SridharKenry, K.Vahabi, HenriKeppert, MartinVaiana, RosolinoKerch, GarryVajragupta, NapatKeresztes, ZsófiaVakros, JohnKern, FrankVala, JiriKern, MatthiasValadez, AlexKern, WolfgangValange, SabineKertmen, AhmetValdez-Nava, ZarelKeshtgar, AzadehValeev, DmitryKessler, SylviaValente, Manuel De AlmeidaKeyvani, AhmadValente, Nicola A.Khabas, Tamara AndreevnaValentin, JanKhac Hoang Bui, VuValerga Puerta, Ana PilarKhachatryan, KarenValérie, MaravalKhadem, Sayyed AhmadValiev, DamirKhadka, Dhruba B.Valiev, RuslanKhadtare, ShubhangiValikhani, AlirezaKhahro, Shabir HussainValinger, DavorKhaimovich, AlexanderValkov, S.Khakalo, SergeiVallejo-Giraldo, CatalinaKhalaj, GholamrezaVallellano, CarpoforoKhalaj, OmidValvo, Paolo S.Khaled, AhmedVamanu, EmanuelKhalfallah, AliVan Den Boogaard, A.H.Khalid, Hammad RazaVan Hemelrijck, DannyKhalighi, SanazVan Herk, AlexKhaloo, AlirezaVan Horn, Ryan M.Khan, AbbasVan Petegem, StevenKhan, AbdulVan Rooyen, Algurnon S.Khan, AdamVan Tittelboom, KimKhan, AdnanVancsó, PéterKhan, AfrasayabVandeginste, VeerleKhan, AjmirVandoren, BramKhan, AnzarVanEpps, J. ScottKhan, Asad UllahVanetsev, AlexanderKhan, HilalVapnik, YevgenyKhan, KifayatVaradwaj, Pradeep R.Khan, M. IqbalVarbai, BalázsKhan, Md. ShakhaoathVardhan, HarshKhan, MehranVarevac, DamirKhan, Mohammad EhtishamVarga, GáborKhan, Mohd ParvezVarga, GyulaKhan, Muhammad FarooqVarga, MariánKhan, Muhammad SaadVargas Salgado, Carlos AfranioKhan, Muhammad UmarVarnagirs, ŠarūnasKhan, Noor SaeedVarone, AlessandraKhan, Omar F.Varveri, Aikaterini (Katerina)Khan, Rabia SannamVasconcelos, Helena CristinaKhan, Shakeel AhmadVasconcelos, Mário RamalhoKhan, Sher AfghanVaseashta, AshokKhan, SovannVashist, SandeepKhan, Taj MuhammadVashisth, AniruddhKhandaker, MorshedVasic, MilicaKhanna, Aniruddh JagdishVasicek, OndrejKhannanov, Arthur A.Vasilev, EvgeniiKhanolkar, AmeyVasilevskaya, ValentinaKhanra, NandishVasilić, RastkoKharaghani, DavoodVasilievici, GabrielKharin, ViktorVasilko, KarolKharitonov, DmitryVasilyev, Aleksander V.Kharmanda, GhaisVasilyeva, MariaKharouf, NajiVaško, MilanKhatib, JamalVassalini, IreneKhatir, SamirVassiliadis, SavvasKhayyat, MahaVasylkiv, Oleg O.Khimich, Margarita A.Vatandoost, HosseinKhitab, AnwarVatin, NikolaiKhodadadian, AmirrezaVatin, Nikolay IvanovichKhomenko, Maxim. D.Vaughan, MelKhomenkova, LarysaVaz, Pedro D.Khomutov, MaximVedyagin, Aleksey A.Khon, Yu. A.Veeran Ponnuvelu, DineshKhorasani, Amir MahyarVega, AlejandroKhorkov, KirillVeiga, FernandoKhoshnood, AbbasVeiga-Suárez, FernandoKhotib, JunaidiVejpravová, Jana KalbacovaKhovaylo, Vladimir V.Vekinis, GeorgeKhubezhov, Soslan A.Velasco Lopez, Francisco JavierKhudyakov, Igor VVelasco Ortega, EugenioKhushnood, ArsalanVelazquez-Perez, Jesus E.Kiani, AmirkianooshVeleva, Lucien P.Kibria, Md. GolamVelgosova, OksanaKichanov, SergeyVelhinho, Alexandre José Da CostaKiefer, RudolfVelicu, RaduKierzek, KrzysztofVelkavrh, IgorKiesiewicz, GrzegorzVeloso, AntonioKihara, TakumiVelpula, Ravi TejaKijanka, PiotrVelu, RajkumarKijenska, EwaVen Den Bergh, WimKijo-Kleczkowska, AgnieszkaVener, Mikhail V.Kikhtyanin, OlegVenetis, JohnKikionis, StefanosVenezuela, JeffreyKilikevičius, SigitasVeniali, FrancescoKim, Ae-rhanVenkatachalapathy, VishnukanthanKim, Birm-JuneVenkatesan, Jagadeesh K.Kim, Bong HoonVenkatraman, Prabhuraj D.Kim, BongjuVenketeswaran, AbhishekKim, BoyongVenugopal, DhamodharanKim, BumjooVera, TeresaKim, Byung-hoonVerbruggen, StefaanKim, Chan-JungVercelli, BarbaraKim, Choong-unVerchenko, ValeriyKim, DaeyoungVerdi, CarlaKim, DonghyunVeremchuk, Igor V.Kim, Dong-JooVereshchaka, Alexey A.Kim, Dong-YeopVergara, DiegoKim, DoyoonVergara, JoséKim, Eui SooVerleysen, PatriciaKim, Gue-HyunVermant, JanKim, GyuyongVermeşan, HoraţiuKim, HaegyeomVernardou, DimitraKim, Hae-WonVernick, SefiKim, Han-JunVeronesi, FedericoKim, Heung-SikVeropalumbo, RosaKim, Hoe JoonVerre, SalvatoreKim, Hye-JinVerros, George D.Kim, HyeminVértesy, GáborKim, HyeokVerucchi, RobertoKim, Hyeong JoonVeselý, MartinKim, HyeongjooVessby, JohanKim, Hyeong-JunVeverka, JakubKim, Hyeong-YeolVezin, HervéKim, Hyo JungVeziroglu, SalihKim, HyosimVia, BrianKim, HyungjinVicente-Manzanares, MiguelKim, HyunsungVicini, SilviaKim, InhwanViczek, SandraKim, In-RyoungVidakis, NectariosKim, Jae YongVidal, CatarinaKim, JedoVidal, JorgeKim, JeonggukViegas, CarlosKim, JeonghunVieira, ManuelKim, JihongVigdorowitsch, MichaelKim, Ji-HwanVignali, AdrianoKim, Jin ManVignali, ValeriaKim, JingyunVij, HiteshKim, JinseokVijayakumar, SekarKim, JinwooVijayan, Vineeth M.Kim, Jong MinVijayaraghavan, RajaniKim, Jong WonVijayavenkataraman, SanjairajKim, JongbeomVikraman, DhanasekaranKim, Jong-HyunVikrant, KumarKim, Jong-SungViktor Ivanovich, SokolovKim, JoohanVikulova, MariaKim, Jung GiVilà, AnnaKim, Keun-SooVila, Maria CristinaKim, Ki SuVilarinho, FernandaKim, Ki-YoungVilhena, AntónioKim, KookhyunVilla, ElenaKim, Kyung-MinVilla, IreneKim, Min SukVilla, SaraKim, MinkookVillalba Sanchis, IgnacioKim, MinseokVillani, VincenzoKim, MiyoungVillanueva-Llauradó, PaulaKim, Moon-JoVillarino, AlbertoKim, Nam-HoonVimmrová, AlenaKim, SangmoVimonsatit, VanissornKim, SangwonViña, JaimeKIM, Seon TaeVinagre, AlexandraKim, Seon-ChilViňáš, JánKim, Seong-GonVincent, DidierKim, Seong-KyunVincent, RobinKim, SojungVinju Vasudevan, SrinivasanKim, Soo MinVinnik, DenisKim, Soon HeeVinodh, RajangamKim, So-YoungVinokurov, SergeyKim, Su-HyeonVirgala, IvanKim, SuminVirgili, TersillaKim, Sung WonVirkkunen, IikkaKim, SunghanVirt, Ihor S.Kim, SungjunVisa, AureliaKim, TaewanViscusi, AntonioKim, YunjaeViseu, Teresa MariaKindrat, IhorVishnyakov, AlekseyKinuthia, JohnVismara, ElenaKioupis, DimitrisVispo, Nelson SantiagoKirker, Grant T.Visser, RickKirnbauer, AlexanderVitale, EnzaKirpļuks, MiķelisVitiello, ElisaKirsanov, DmitryVitry, VéroniqueKirsimäe, KalleViviani, MassimoKirste, RonnyVizureanu, PetricaKirubaharan, KamalanVizzari, DomenicoKiryukhantsev-Korneev, Ph.V.Vlad, Cătălin IoanKisala, JoannaVlădăreanu, VictorKishimoto, SatoshiVladimir, KodnyankoKishor, KamalVlǎdoiu, RodicaKišiček, TomislavVlaicu, Ioana DorinaKisielewicz, TomaszVlase, SorinKisilowski, JerzyVocanson, FrancisKislov, Evgeniy VladimirovichVogt, Jean-BernardKiss, ImreVogt, JochenKita, AlbanVohra, FahimKitagawa, JiroVoicu, RodicaKitagawa, YasutakaVoit, KlausKitai, AdrianVojtěch, DaliborKitazawa, TakafumiVojtko, MarekKizek, JánVojvodikova, BarbaraKlačková, IvanaVokoun, DavidKlapötke, Thomas M.Volk, WolframKlar, AgnesVolkova, Elena G.Klaudio, BariVollbrecht, JoachimKlebert, SzilviaVollmer, MalteKlein, ThomasVollprecht, DanielKlekiel, TomaszVolmer, MariusKlemenc, JernejVolodin, Alexander M.Klementeva, SvetlanaVolodin, VladimirKlemes, JiriVolosova, Marina A.Kleszczynski, StefanVolotka, AndreyKlimecka-Tatar, DorotaVolpe, AnnalisaKlimov, AleksandrVolpp, JoergKlimowicz, AdamVon Dreele, RobertKlinge, SandraVon Gratowski, SvetlanaKlini, ArgyroVona, DaniloKliukas, RomualdasVopálka, DušanKlobcar, DamjanVora, AnkitKlöcker, HelmutVora, Jay J.Klontzas, MichailVordos, NickKlose, AndrewVorhauer, NicoleKlötzli, UrsVorndran, ElkeKluczynski, JanuszVoronin, Denis V.Klusak, JanVorotilo, StepanKlusemann, BenjaminVorotylo, StepanKłysz, SylwesterVorozhtsov, Alexander B.Klyui, Nickolai I.Voshavar, ChandrashekharKminiak, RichardVoshkin, AndreyKmita, AngelikaVostrikova, Kira E.Knight, ChristopherVourlias, GeorgeKnite, MārisVoutetaki, Maristella E.Knitschke, MichaelVoyles, Andrew S.Knitter-Piątkowska, AnnaVoznesenskii, Aleksandr S.Knyazeva, Anna GeorgievnaVrabec, MirijamKnych, TadeuszVranceanu, Diana M.Knyziak, PiotrVrestal, JanKo, Chun-HanVrinceanu, NarcisaKo, Dae-CheolVrsaljko, DomagojKobayashi, JunVu, Danh C.Kobayashi, MikiVuchkov, TodorKobayashi, SatoruVujkovic, MilicaKoberová Ivančaková, RomanaVukelic, DjordjeKobetičová, KláraVutova, KatiaKoblischka-Veneva, AnjelaVyas, VijayKobtsev, SergeyVymazal, TomášKobyłka, RafałW. Nicholson, JohnKochmańska, AgnieszkaWaag, FriedrichKochmański, PawełWadati, HirokiKočiško, RóbertWaddell, NeilKoczorowski, TomaszWadham, CarolKoda, EugeniuszWager, JohnKoduru, JanardhanWagh, Priyesh A.Koenders, EduardusWagner, StefanKoenig, AndreasWainstein, DmitryKogut, IuriiWajima, TakaakiKohno, Yoshiumi I.Wakai, EiichiKoibuchi, HiroshiWald, FrantisekKoike, MariWales, DominicKoilraj, PaulmanickamWałęsa, KrzysztofKoivisto, JanneWałęsa-Chorab, MonikaKoizumi, HiroyasuWalock, MichaelKojic, MilosWalowski, GrzegorzKojima, KazutoshiWalsh, Frank C.Kokkinos, Nikolaos C.Walters, David J.Koklu, UgurWalther, ThomasKoksharova, Olga A.Wan, DiKolahchi, RezaWan, LongKolar, PraveenWandelt, KlausKolaric, BrankoWang, A-ChengKolasińska, EwaWang, AiqinKölbl, NathalieWang, AiyingKolcu, FeyzaWang, AnyangKolenak, RomanWang, BaominKolesnikov, GennadiyWang, BaotianKolesov, Boris A.Wang, BinKoleva, Iskra Z.Wang, BoKolhatkar, GitanjaliWang, ChanghongKollmannsberger, StefanWang, ChaoKolmakov, Alexey G.Wang, Chih-MinKolmas, JoannaWang, Ching-HsinKolmychek, Irina A.Wang, ChungeKolokolov, DaniilWang, ChunhuiKolota, AleksandraWang, ChunjinKolovos, Konstantinos G.Wang, Chun-KaiKolpakov, A.G.Wang, DanqingKoltsov, IwonaWang, DaweiKolya, HaradhanWang, DayongKomarek, AlexanderWang, DengjunKomarov, Pavel V.Wang, DiKomarov, VictorWang, DongKomarova, EkaterinaWang, FaxingKomasa, SatoshiWang, FeiKombaiah, BoopathyWang, GuiKomelj, MatejWang, GuofengKomizo, Yu-ichiWang, GuoqingKomogortsev, SergeiWang, HaidongKomova, OxanaWang, HaiouKoňáková, DanaWang, HaitaoKonar, ArkaprabhaWang, HaiyangKonarev, Dmitri V.Wang, HaoyuKończyk, JoannaWang, HuanKonda Gokuldoss, PrashanthWang, HuapingKondekar, NehaWang, Huei-SenKondinski, AleksandarWang, HuiKondo, TakahiroWang, Jia-ChangKonecny, PetrWang, JialaiKong, Choon YenWang, JianKong, Sih YingWang, JianguoKonieczny, MarekWang, JianjunKoniorczyk, PiotrWang, JianleiKonist, AlarWang, Jian-qiangKonkol, JanuszWang, Jian-WenKonofaos, NikolaosWang, JianxuKonopacka-Łyskawa, DonataWang, JiaqingKonopka, KatarzynaWang, JingyuKonovalov, SergeyWang, JinyanKonovalova, ViktoriyaWang, JunKonował, EmiliaWang, JunjieKonrad, MalickiWang, JunrenKonrád, PetrWang, KeKonstanciak, AnnaWang, LeiKonstantakopoulou, FotiniWang, LeimingKonstantinos, KatakalosWang, LingKontargyri, VassilikiWang, LipingKontonasaki, EleanaWang, LiqiangKontoni, Denise-PenelopeWang, LuKontziampasis, DimitriosWang, MingqingKoo, Bon HeunWang, MingtaoKoo, Bon-YongWang, PanKoo, JahyunWang, PengchengKoos, Antal A.Wang, PengfeiKopáčik, AlojzWang, PingKopeček, JaromírWang, Ping-liKopernik, MagdalenaWang, QiKoplík, JanWang, QianKoplin, ChristofWang, QianqianKoral, CanWang, QiliangKordijazi, AmirWang, QingKoric, SeidWang, QinghuaKorjakins, AleksandrsWang, QingsongKormaníková, EvaWang, QinjunKormos, AtillaWang, QinyiKorneev, DenisWang, RenjieKorniejenko, KingaWang, RuoxingKorobko, Dmitry A.Wang, Shang-TaKorolev, Evgenij ValerjevichWang, ShaomengKorolkov, Ilya V.Wang, ShaoqingKorolovych, VolodymyrWang, ShibingKorosteleva, Elena N.Wang, ShidongKorsunsky, Alexander M.Wang, ShiyanKortaberria, GalderWang, ShuangxiKorupalli, ChiranjeeviWang, SimonKorzekwa, JoannaWang, TianhaoKorzeniowski, WaldemarWang, TianshiKorzhikova-Vlakh, EvgeniaWang, WeiKorznikova, ElenaWang, WeiliangKos, BorWang, WeiqiangKos, ŠimonWang, WenshengKosakowska, OlgaWang, XiaodiKosar, NaveenWang, XinyuKöse, CeyhunWang, XinyunKostadinov, GeorgiWang, XunchangKostopoulos, GeorgiosWang, Yi ShengKostrzanowska-Siedlarz, AleksandraWang, YifeiKoszelewski, DominikWang, YongliangKótai, LászlóWang, ZhenlongKotek, JanWang, ZhihuanKoteš, PeterWang, ZhileiKothapalli, Chandrasekhar R.Wang, ZhongpengKotova, Olga BorisovnaWangenheim, MatthiasKotsanos, NikolaosWan-Wendner, RomanKotsou, MariaWaqas, SaleemKoubiti, MohammedWardeh, GeorgeKoudelka, PetrWardhono, Endarto Y.Kouhia, ReijoWark, MichaelKouril, MilanWashio, AyakoKoutas, LamprosWasige, EdwardKoutavarapu, RavindranadhWasilewski, TomaszKoutselas, IoannisWasim, MuhammadKoutsos, VasileiosWasserman, RinaKoutsovitis, PetrosWatanabe, MotonoriKováč, JaroslavWatarai, HitoshiKováč, MatúšWatari, HisakiKovačević, AndjelkaWatauchi, SatoshiKovačević, DavorWatkins, Anthony NealKovacheva, DanielaWątróbski, JarosławKovacic, MarinWätzig, KatjaKováčik, JaroslavWcisło, AnnaKovács, LászlóWebb, CathleenKovács, Norbert KrisztiánWeber, DanielKovács, RóbertWeber, FranzKovács, ZsoltWeber, MatthieuKovalchenko, Andrii M.Wehrle, ErichKovalchuk, OleksandrWei, Chin-ChungKovalchuk, VolodjaWei, Da-HuaKovalcikova, AlexandraWei, GangKovalev, AnatoliyWei, HaoyanKovaleva, MarinaWei, JianqiangKovalevsky, Andrei V.Wei, QiangKovalov, DanyilWei, QingyangKovářík, OndřejWei, Wang YangKowalczuk, AgnieszkaWei, Wei-ChyouKowalczuk, MarekWei, ZhishunKowalczyk, AgnieszkaWeihe, StefanKowalczyk-Juśko, AlinaWeinert, MichaelKowalewska, AnnaWeise, JörgKowalonek, JolantaWeißensteiner, IrmgardKowalska, EwaWeiwei, ShiKowalski, AleksanderWejrzanowski, TomaszKowalski, BogdanWeller, Michael G.Kowerdziej, RafałWellmann, PeterKoya, Alemayehu NanaWeltsch, ZoltánKoyama, ToshiyukiWen, Dong-XuKoyanagi, JunWen, JialongKozai, NaofumiWen, PengKozakiewicz, MarcinWen, ShihuiKozakiewicz, PawełWeres, JerzyKožar, IvicaWezgowiec, JoannaKozhukhova, Natalia IvanovnaWheeler, D.W.Koziol, MateuszWhite, GregKozior, TomaszWibowo, HartantoKozlova, EkaterinaWickramasinghe, SameeraKozlova, Ekaterina AleksandrovnaWickström, UlfKozłowska, AleksandraWidmer, Karl-HeinzKozłowski, AndrzejWieczorowski, MichalKozub, BarbaraWieleba, WojciechKozyatnyk, IvanWierzbicka, EwaKraft, Thomas M.Wiewiórowska, SylwiaKrajewski, PiotrWiglusz, KatarzynaKrajewski, WitoldWilińska, IwonaKrakowiak, StefanWillert, EmanuelKrál, PetrWilliams, JohnKraljevic, SonjaWilliams, Nicholas X.Kralj-Iglic, VeronikaWilson, Christopher J.Kralov, IvanWilson, Gregory J.Kralovec, ChristophWilson, PeterKramar, AnaWimbush, StuartKrampikowska, AleksandraWinczek, JerzyKrankenhagen, RainerWiniarski, Michał J.Kranz, StefanWiniczenko, RadosławKrasilin, AndreiWiniecki, MariuszKrasowska, MonikaWinkler, AnjaKrastev, RumenWirth, Hans JürgenKratochvilova, IrenaWise, WillKrauklis, AndreyWiskel, J. BarryKraus, IvanWisniewski, AdamKräusel, VerenaWiśniewski, KrzysztofKravanja, GregorWisniewski, PiotrKrawczak, PatriciaWissman, James P.Krawczyk, JacekWiszumirska, KarolinaKrawczyk, JanuszWittmann, FolkerKrawiec, HalinaWłodarczyk, RenataKrawiec, PiotrWlokas, IrenaeusKrbaťa, MichalWnorowski, AndrzejKrebsz, MelindaWodo, OlgaKrejčí, TomášWojasiński, MichałKrella, AlicjaWojciech, GilewskiKremieniewski, MarcinWojciechowski, SzymonKrenicky, TiborWojcieszak, DamianKreuzpaintner, WolfgangWójcik, Natalia A.Krezhov, KirilWójcik-Bania, MonikaKrinitcyn, MaksimWojnarowicz, JacekKrishnan, SitaramanWojtaszek, MarekKrishnaprasad, RajanWołczyński, WaldemarKristak, LubosWolfram, VolkKristiansen, HelgeWolińska, EwaKriston, AkosWollweber, JuergenKrivenko, PavelWolniak, RadosławKrivovichev, Sergey V.Wolny, StefanKrivovichev, VladimirWon, JongsungKrmela, JanWondraczek, KatrinKroeker, S.Wong, BryanKröger, NadjaWong, Ka KanKrólczyk, JolantaWong, Leong SingKrólicka, AgnieszkaWoodhead, IanKrólicka, AleksandraWoojae, ChoiKromka, AlexanderWorrall, StephenKrontiras, ChristoforosWosatko, AdamKrsjak, VladimirWośko, MateuszKruis, JaroslavWoszuk, AgnieszkaKruk, AdamWoyciechowski, Piotr PawełKruk, AndrzejWozny, JanuszKrukowski, StanislawWragg, David S.Kruml, TomášWrobel, KamilKrupa, Kristen A.Wróbel, Paweł S.Krupińska, BeataWróblewska, AnnaKrupińska, IzabelaWróblewski, RomanKrupinski, MichalWu, ChaoKruszewski, MirosławWu, Chia-WenKrylov, Alexander S.Wu, Chin-SanKryschi, CarolaWu, HuiKrystofiak, TomaszWu, JiaminKrzan, MarcelWu, JieyunKrzanowski, James E.Wu, Jing-YunKrzeminski, MarekWu, JudyKrzywinski, KamilWu, KaigeKrzywoń, RafałWu, Ming-ChungKrzywoszynska, KarolinaWu, PengKrzyżak, AnetaWu, Pu-WeiKshirsagar, AnurajWu, ShaohuaKsiążek, MarzannaWu, ShenghuaKsibi, MohamedWu, WeiKtena, AphroditeWulfman, ClaudineKuang, PengWunderlich, WilfriedKuang, TairongWurster, StefanKübarsepp, JakobWychowaniec, JacekKubasiewicz-Ross, PawelWychowański, PiotrKubiak, AdamWysmulski, PawełKubiak, KrzysztofWysocki, BartłomiejKubiak, TomaszXanthopoulou, VayiaKubica, JanXavier, JoséKubík, MichalXavier, José Manuel CardosoKubissa, WojciechXia, ChangleiKubit, AndrzejXia, QiKubo, Anna-LiisaXiao, BolvKubo, AtsushiXiao, JianqiuKubo, MasatakaXiao, LijunKubo, TakanoriXiao, RuiKuca, KamilXiao, ZhongminKučerka, DanielXie, DongyueKucharczyk, BarbaraXie, NingKucharczyková, BarbaraXie, TianyuKucharska, BarbaraXie, WanfengKucharska, KarolinaXin, HaiKuchi, RambabuXiong, BijinKuchler-Bopp, SabineXu, ChengyiKucinska, MalgorzataXu, GangKuckling, DirkXu, JohnKuddannaya, ShreyasXu, JunKudelski, RafałXu, JuncaiKudryashova, OlgaXu, MinKuehne, Alexander J. C.Xu, QinKuehne, IrinaXu, ShiKuhn, RamonaXu, WenhanKühne, Irina A.Xu, WenwuKühnisch, JanXu, ZhigangKujawa, JoannaXuan, TongtongKujawska, JustynaXue, DongfengKujawski, DanielXue, JiaxiangKukla, ChristianXuejuan, CaoKukli, KaupoYadav, Dharmendra K.Kukovec, Boris-MarkoYadav, Hemraj MKukushkin, Sergey A.Yagami, KimitoshiKul, EsraYagati, Ajay KumarKula, KrzysztofYaghoubi, EhsanKula, SławomirYahya, EsamKulagin, RomanYaici, WahibaKulczycki, AndrzejYakout, MostafaKulda, VlastimilYakovlev, GrigoryKuliczkowski, KazimierzYakovleva, ValentinaKulik, Leonid V.Yakuhina, AnastasiaKulikova, Natalya A.Yakushina, EvgeniaKulinich, SergeiYalavarthi, RambabuKulka, MichalYalcin, ErkutKulovits, AndreasYamada, ShigeyukiKulyk, VolodymyrYamaguchi, SeijiKumar Kamboj, NikhilYamamoto, GoKumar, AbhishekYaman, UlasKumar, AnujYan, FuyaoKumar, ArvindYan, JiaqiKumar, AshutoshYan, JinglinKumar, AvneeshYan, ShoukeKumar, BijenderYan, WeiKumar, DeepakYanagida, TakayukiKumar, DharmeshYang, BinKumar, JayanthiYang, ChenKumar, M. VijayYang, Cheng-FuKumar, ManishYang, Chen-IKumar, MukeshYang, Chih-ChengKumar, PawanYang, ChunguangKumar, RajeevYang, Chung-WeiKumar, SandeepYang, ChunleiKumar, SanjayYang, FeipengKumar, ShailabhYang, FuqianKumar, VibhorYang, HailinKumar, VineetYang, HuajieKumari, PreetiYang, JianjunKumavath, RanjithYang, JieKumchai, HattanasYang, Kai MinKümmel, FrankYang, KejiaKumpikaitė, EglėYang, MijiaKundu, JoyjitYang, MingKunowsky, MirkoYang, NiKuntoğlu, MustafaYang, PengKuntsevich, Alexander YuYang, QuanlongKuntz, MatthiasYang, ShuyangKünzel, HartwigYang, SungchulKuo, Che NanYang, Teng-ChunKuo, Dong-HauYang, ThomasKuo, Jui-ChaoYang, ZhiboKuo, Wen-TenYao, BinKupai, JozsefYao, WeiKupriyanov, Igor N.Yap, Pei LayKurakula, MalleshYaparla, AmulyaKurc, BeataYaqub, TalhaKurda, RawazYaroslavtsev, Andrey B.Kureha, TakumaYasin, SohailKurek, AndrzejYasuda, HiroshiKurganskaya, InnaYasuo, TakahashiKurlyandskaya, Galina V.Yazdani Asrami, MohammadKurmus, HalenurYe, BingKurniawan, RendiYe, HailongKurniawan, Tonni AgustionoYe, Shu-fengKuropka, PiotrYeboah, DavidKurpinska, MarzenaYee, Daryl W.Kurushina, VictoriaYeh, Chun-LiangKurylo, PiotrYeih, Wei-ChungKurzydłowski, Krzysztof J.Yellavajjala, Ravi KiranKusior, AnnaYeo, Joo ChuanKusmartseva, AnnaYeo, Sang-youngKusmic, DavidYeon, JaeheumKušnerová, MilenaYepes, VíctorKuşoǧlu, Ihsan MuratYerramilli, Aditya S.Kusterle, WolfgangYesudhas, SorbKustov, SergeyYeum, JeongKusuma, HeriYeung, Andy Wai KanKusuma, Victor A.Yi, HwangKusumi, RyosukeYick, Kit-LunKutty, MuralithranYidris, NoorfaizalKuz′min, Mikhail P.Yilmaz, MehmetKuzhir, PolinaYin, ChaoKuzhir, Polina P.Yin, GuoweiKuziak, JustynaYin, JianboKuzmichev, VictorYin, JieKuzmin, AlexeiYin, KaiyangKuznetsov, Alexey N.Yin, RongKuznetsov, Pavel VictorovichYip, JoanneKuznetsova, YuliaYokose, SatoshiKuźnicka, BogumiłaYokota, HiroshiKvačkaj, TiborYong, Kar WeyKvande, ToreYong, ShengKvítek, OndřejYoo, ByungseokKwak, SeonyeongYoo, Chung-YulKwame, James S.Yoo, Dong JinKwan, CharlesYoo, HocheonKwiecień, IwonaYoo, HosikKwiecień, MarcinYoon, Do YeungKwolek, PrzemysławYoon, Hyung-InKwon, Ji EonYoon, JinhoKwon, KyungjungYoon, JonghunKwon, Oh SeokYoon, MinyoungKwon, Soon-HongYoon, Seog-YoungKwon, Sung-NamYoshida, KentaroKwon, Tae-YubYoshida, SanichiroKyratsis, PanagiotisYoshimatsu, KoheiKytinou, ViolettaYoshioka, TomohikoL, NatrayanYou, Hye JinLa Magna, AntoninoYou, IlhwanLa Mantia, Francesco PaoloYou, Tae-SooLa Plante, ErikaYounes, HammadLa Rosa, GuidoYounes, HusamLa Verde, GiuseppeYoung, J. D.Łabanowski, JerzyYoung, JamieLabchir, NabilYoung, StephenLaber, KonradYounus, HusseinLabianca, ClaudiaYousefi, AliLaccone, FrancescoYoussry, MohamedLachowicz, MarzenaYu, HaiboLacivita, ValentinaYu, HaiyangLadavos, Athanasios K.Yu, Ing-SongLafont, UgoYu, Jung-DoungLaforgia, AlessandraYu, NingLaGrow, Alec P.Yu, Ollie YiruLähde, AnnaYu, Ruei-SungLahmer, TomYu, TiantangLai, Ping-ShanYu, WenbinLai, QinghuaYu, ZiniuLai, Wei-ChihYuan, BinLai, Wing-FuYuan, Chi-TsuLaín, SantiagoYuan, Chiun-JyeLaity, Peter RYuan, DianLam, Dra. ElizabethYuan, GuanghuiLam, Walter Yu HangYuan, RuiLamanna, GiuseppeYucel Yardimci, MertLambert, PaulYue, ShengyingLamberti, FrancescoYue, ShiyuLamberti, PatriziaYugami, HirooLamberti, Vincent E.Yun, Hyun DoLambin, PhilippeYun, JoosunLampakis, DimitriosYung, Tung-YuanLampre, IsabelleYurkov, AndreyLampropoulou, ParaskeviYurukcu, MesutLamura, AntonioYusenko, Kirill V.Lan, Ting-HsunYushina, IrinaLan, Wen-HowYusuf, ShahirLanba, AsheeshZaba, KrzysztofLancea, CamilZabala, AlaitzLandi, FilippoZabierowski, PiotrLandi, GianlucaZablotsky, DmitryLandi, SalmonZacchei, EnricoLang, GregorZaccone, AlessioLangdon, GenevieveZach, RyszardLanger, Jerzy J.Zacharis, Constantinos K.Langford, StevenZaczyńska, MonikaLanjewar, HarishchandraZafar, MuhammadLanotte, MicheleZafar, Muhammad SohailLanzafame, Raymond JZaffora, AndreaŁapińska, AnnaZagklis, DimitrisŁapińska, BarbaraZaharescu, TraianLapkowski, MieczyslawZaharia, CatalinLapushkina, TatianaZahedi, AbolfazlLarciprete, RosannaZahedi, AliLarrañaga, AitorZahir, Md. HasanLarsson, ChristelZahoranová, AnnaLarsson, Per-LennartZaidi, AliLarsson, SimonZaidi, Shabi AbbasLaskowska, AgnieszkaZairi, FahmiLasri, TuamiZairov, RustemLassandro, PaolaZając, WojciechLászló, KrisztinaŻak, AndrzejLatko-Durałek, PaulinaŻak, KrzysztofLau, AlanŻak, Paweł LeszekLaubenstein, MatthiasZakharov, Alex V.Laukkanen, AnssiZakowski, KrzysztofLaurano, RossellaZaky, MohamedLaureti, StefanoZalameda, Joseph NLaventure, AudreyŽalga, ArtūrasLaw, David W.Zambon, AlfonsoLaw, Jia YanZambon, DanielLazar, Adriana VulpoiZamboulis, AlexandraLazarides, NikosZambrano, OscarLazarova, KaterinaZambrano-Robledo, Patricia C.Łaźniewska-Piekarczyk, BeataZamparini, FaustoLazorenko, GeorgyZampetti, EmilianoLazzara, GiuseppeZamudio-Flores, Paul BarukLazzari, MassimoZangena, Shaban AliLe Cann, SophieZangeneh-Nejad, FarzadLe Febvrier, ArnaudZanger, FrederikLe Floch, SylvieZani, NicolaLe Gac, SéverineZanini, FrancoLe Normand, FrancoisZanjanijam, Ali RezaLe Tellier, RomainZanoletti, AlessandraLe, Kim QuyZanuttini, RobertoLe, Phuong MaiZanuy, CarlosLe, Thanh HaiZaragoza Contreras, Erasto ArmandoLe, Tien-ThinhZardán Gómez De La Torre, TeresaLe, Van PhucZare, ArezooLe, Viet-DucZare, Ehsan NazarzadehLe, Vinh TungZareie, ShahinLebedeva, Olga E.Zarejousheghani, MashaalahLebyodkin, Mikhaïl A.Zarepour, AtefehLech, SebastianZargar, OmidLechner, PhilippZarkadoulas, AthanasiosLeCloirec, PierreZarkov, AleksejLecomte, PhilippeZarrelli, MauroLedwon, PrzemyslawZarrintaj, PayamLee, AlbertZarzycki, ArkadiuszLee, Bang YeonZatsepin, Anatoly F.Lee, Bong-KeeZavašnik, JanezLee, ByeongchanZavoianu, RodicaLee, Byoung-SunZaytsev, AlexeyLee, Byung JaeZbiciak, ArturLee, ChangguZdorovets, MaximLee, Chia-HungZdraveva, EmilijaLee, Chih-HaoZdunek, JoannaLee, Chi-ShenZecevic, MiroslavLee, Chuan-PeiZednik, RicardoLee, ChulminZehnder, MatthiasLee, Chung-SungZekker, IvarLee, Czang-HoŻelaziński, TomaszLee, DaehoZeleniakiene, DaivaLee, Dai-SooZelepugin, Sergey A.Lee, Dong HyunZelinka, SamuelLee, DongkyuZeman, PavelLee, DongyunZemanová, MatildaLee, Eui-SeokZemková, ErikaLee, EunkyungZemskov, AndreiLee, EunsongyiZeng, CongyuanLee, Gee-SooZervaki, Anna D.Lee, Gyo-wooZetterling, Carl-MikaelLee, Han EolZeyad, Abdullah M.Lee, HeeyoungZgardzińska, B.Lee, Hong-KiZgodavová, KristínaLee, Hosin (David)Zgură, IrinaLee, Hyun HoZha, JunLee, IlkeunZhai, Wei-dongLee, In-SeopZhanal, PavelLee, I-TaZhang, BinLee, JaegeunZhang, BinshengLee, JaehyunZhang, ChaominLee, Jae-SeungZhang, ChaoqunLee, JaewooZhang, ErshuaiLee, Jang-HyunZhang, FanLee, JeonghoonZhang, GuowuLee, Jin-KyunZhang, HuLee, Jin-YoungZhang, HuiLee, JiseokZhang, JiangguoLee, Jong BongZhang, JianliangLee, JongbokZhang, JinbaoLee, Jong-HoZhang, JohnsonLee, JoonhoZhang, KaiLee, Ju-HyuckZhang, MengLee, Jung GuZhang, MiaoLee, Jung SeungZhang, MingliangLee, Jung-KulZhang, QianLee, Kee HagZhang, QichunLee, Kee SungZhang, RenwuLee, Ki-sunZhang, RuiLee, Kwang JoZhang, RuimengLee, Kyung JinZhang, RuiyongLee, Nam KonZhang, ShenliLee, Rong-HoZhang, ShuyeLee, Sang-SupZhang, TaoLee, Seok-JaeZhang, WeidongLEE, SoonJaeZhang, WeizhaoLee, SujeongZhang, WenbinLee, Sung-MinZhang, WengangLee, SungyulZhang, YongLee, TaegyuZhao, ChunLee, TaekyungZhao, DonglinLee, Wei-HaoZhao, HaiyanLee, Wen-JenZhao, JingyiLee, Won ChulZhao, MeipingLee, Wook JinZhao, MingruiLee, WookjinZhao, WeiLee, Yong-JooZhao, YongqingLEE, YulinZheng, ErjinLeese, HannahZheng, HuaiLegutko, StanisławZheng, KaiLehmkühler, FelixZheng, ShuanghaoLehner, PetrZheng, SunxiangLehr IvanaZherebker, AlexanderLei, MeiZhidkov, Ivan S.Leineweber, AndreasZhigachev, Andrey O.Leiske, Meike NicoleZholobko, OksanaLeitner, MichaelZhong, WeibingLeiva-Padilla, PaulinaZhou, AoŁękawa-Raus, AgnieszkaZhou, ChangyuLeklou, NordineZhou, HaiboLemaitre, GerardZhou, HongLemma, EnricoZhou, JieLemos, AgostinhoZhou, RenwuLengvarský, PavolZhou, WeiweiLenrick, FilipZhou, WenbinLenser, ChristianZhu, HuiLenzi, Giane G.Zhu, JiangongLeong Sing, SweeZhu, JianxiongLeonida, AlessandroZhu, JuanjuanLepadatu, DanielZhu, WenliangLepareur, NicolasZhu, XuanLeporatti, StefanoZhukov, IlyaLeporini, DinoZhuo, WeihaiLeriche, AnneŽibret, GorazdLesar, BoštjanZidarič, TanjaLesiuk, GrzegorzZidek, JanLeśniak, DariuszZielecka, MariaLesovik, Valery StanislavovichZieliński, AdamLester, EdwardZieliński, AndrzejLeszczyńska-Madej, BeataZielinski, PiotrLeszczyńska-Sejda, KatarzynaZienert, TiloLeszczyński, JacekŽigon, JureLetardi, PaolaZima, AnetaLettieri, StefanoZima, BeataLeung, KenZimmerman, WilliamLevei, Erika AndreaZinigrad, MichaelLevesque, PierreZinno, RaffaeleLewandowska, KatarzynaZinovi, DashevskyLewis, David A.Ziółkowski, PatrykLewis, GladiusZivaljic, NikolinaLežaić, Aleksandra JanoševićZivkovic, VladimirLeżańska, MariaZlotnik, SebastianLezov, AlexeyZmarzły, PawełLi Voti, RobertoŽmindák, MilanLi, BinŻmudzki, JarosławLi, ChengmingZolotukhin, Denis B.Li, Chia-LinZoltán, PásztiLi, ChunZona, AlessandroLi, DayongŻórawski, WojciechLi, FangsenZornoza, EmilioLi, GuangliZorpas, AntonisLi, HaoZotti, AldobenedettoLi, HongdongZotti, FrancescaLi, HongguangZou, HaiyangLi, HuafangZuback, JamesLi, JianZubar, Tatiana I.Li, JiantongZubrzycki, JarosławLi, JiaqiZuccarello, BernardoLi, JiehuaŽukowski, Paweł V.Li, Ji-QinZukowski, WitoldLi, JunŽunda, AudriusLI, Jun-haoZuo, PeiyuanLi, JunruiZuorro, AntonioLi, KunZupanič, FrancLi, MengfangZurauskiene, RamuneLi, MingxingZurek, JoannaLi, Ming-YuZuzolo, DanielaLi, PeipeiZverev, VladimirLi, PenghaoZwirner, JohannLi, PingZych, DawidLi, QingbinZych, TeresaLi, Rita Yi ManZyryanov, Grigory V.Li, RuiZyska, AndrzejLi, ShuoZywica, GrzegorzLi, Sipei


